# Reactive oxygen species, toxicity, oxidative stress, and antioxidants: chronic diseases and aging

**DOI:** 10.1007/s00204-023-03562-9

**Published:** 2023-08-19

**Authors:** Klaudia Jomova, Renata Raptova, Suliman Y. Alomar, Saleh H. Alwasel, Eugenie Nepovimova, Kamil Kuca, Marian Valko

**Affiliations:** 1 Department of Chemistry, Faculty of Natural Sciences, Constantine the Philosopher University in Nitra, Nitra, 949 74 Slovakia; 2 Faculty of Chemical and Food Technology, Slovak University of Technology, Bratislava, 812 37 Slovakia; 3 Zoology Department, College of Science, King Saud University, 11451 Riyadh, Saudi Arabia; 4 Department of Chemistry, Faculty of Sciences, University of Hradec Kralove, 50005 Hradec Kralove, Czech Republic

**Keywords:** Oxidative stress, Metals, ROS, Antioxidants, Signaling pathways, Aging, Toxicity

## Abstract

A physiological level of oxygen/nitrogen free radicals and non-radical reactive species (collectively known as ROS/RNS) is termed oxidative eustress or “good stress” and is characterized by low to mild levels of oxidants involved in the regulation of various biochemical transformations such as carboxylation, hydroxylation, peroxidation, or modulation of signal transduction pathways such as Nuclear factor-κB (NF-κB), Mitogen-activated protein kinase (MAPK) cascade, phosphoinositide-3-kinase, nuclear factor erythroid 2–related factor 2 (Nrf2) and other processes. Increased levels of ROS/RNS, generated from both endogenous (mitochondria, NADPH oxidases) and/or exogenous sources (radiation, certain drugs, foods, cigarette smoking, pollution) result in a harmful condition termed oxidative stress (“bad stress”). Although it is widely accepted, that many chronic diseases are multifactorial in origin, they share oxidative stress as a common denominator. Here we review the importance of oxidative stress and the mechanisms through which oxidative stress contributes to the pathological states of an organism. Attention is focused on the chemistry of ROS and RNS (e.g. superoxide radical, hydrogen peroxide, hydroxyl radicals, peroxyl radicals, nitric oxide, peroxynitrite), and their role in oxidative damage of DNA, proteins, and membrane lipids. Quantitative and qualitative assessment of oxidative stress biomarkers is also discussed. Oxidative stress contributes to the pathology of cancer, cardiovascular diseases, diabetes, neurological disorders (Alzheimer’s and Parkinson’s diseases, Down syndrome), psychiatric diseases (depression, schizophrenia, bipolar disorder), renal disease, lung disease (chronic pulmonary obstruction, lung cancer), and aging. The concerted action of antioxidants to ameliorate the harmful effect of oxidative stress is achieved by antioxidant enzymes (Superoxide dismutases-SODs, catalase, glutathione peroxidase-GPx), and small molecular weight antioxidants (vitamins C and E, flavonoids, carotenoids, melatonin, ergothioneine, and others). Perhaps one of the most effective low molecular weight antioxidants is vitamin E, the first line of defense against the peroxidation of lipids. A promising approach appears to be the use of certain antioxidants (e.g. flavonoids), showing weak prooxidant properties that may boost cellular antioxidant systems and thus act as preventive anticancer agents. Redox metal-based enzyme mimetic compounds as potential pharmaceutical interventions and sirtuins as promising therapeutic targets for age-related diseases and anti-aging strategies are discussed.

## Introduction

Free radicals are chemical entities containing at least one unpaired electron in the outer shell which usually gives them high reactivity. The most frequently occurring free radicals and reactive molecules in biological systems are derived from oxygen (reactive oxygen species, ROS) and nitrogen (Reactive Nitrogen Species, RNS). ROS or RNS are formed during electron transfer reactions, by losing or accepting electron(s) (Halliwell and Gutteridge 2015).

ROS are important players in cellular proliferation, differentiation, migration, apoptosis, and necrosis. Low to intermediate levels of ROS and RNS are necessary for the maintenance of many important physiological functions, redox homeostasis, and the regulation of key transcription factors. In contrast, excessive formation of ROS is responsible for disrupted redox homeostasis which in turn leads to oxidative stress and ROS-mediated damage to all important biomolecules including DNA, proteins, and membranes (Liguori et al. 2018). Oxidative stress, characterized by a shifted equilibrium between the formation and elimination of free radicals toward the formation, is a common denominator of the pathogenesis of chronic diseases and aging (Harman 1956; Valko et al. 2007).

The level of oxidative stress, quantified by the oxidative stress markers, has been found elevated in aging individuals or people with bad lifestyles, taking unhealthy food, smoking, drinking alcohol, lack of physical exercise, and genetic predisposition. Cells have evolved a complex antioxidant system, formed by enzymatic antioxidants, such as superoxide dismutases (SODs), catalase (CAT) and glutathione peroxidases (GPXs), thioredoxin (Trx) as well as low molecular antioxidants, whose concerted action is balancing the level of oxidative stress (Halliwell 2022). A complex antioxidant defense system, including both endogenous and exogenous antioxidants, protect cells from ROS-induced toxicity (Finkel and Holbrook 2000).

The aim of this paper is to review the role of ROS in normal physiological functions, ROS-mediated cellular signaling, and the role of oxidative stress in various chronic diseases and aging. Attention is also paid to the effect of antioxidants to alleviate the level of oxidative stress, which is a common denominator of various chronic diseases such as neurological disorders, cancer, cardiovascular, renal, lung, metabolic, neurological, psychiatric, and other diseases.

## Chemistry and sources of ROS and RNS in biological systems

Molecular oxygen in a triplet state contains two unpaired electrons with parallel spin states (↑↑) in antibonding π^*^ orbitals and therefore is a biradical. Since chemical bonds of biomolecules are formed by two antiparallel electrons (↑↓), their interaction (oxidation) with molecular dioxygen (containing two parallel electrons) is spin restricted and therefore rather slow. Due to the parallel orientation of both electrons (electron spin *S* = 1), molecular oxygen is sometimes referred to as triplet oxygen (multiplicity 2 × *S* + 1 = 2 × 1 + 1 = 3) and is denoted as ^3^O_2_ (Halliwell and Gutteridge 2015).

**Fig. 1:**
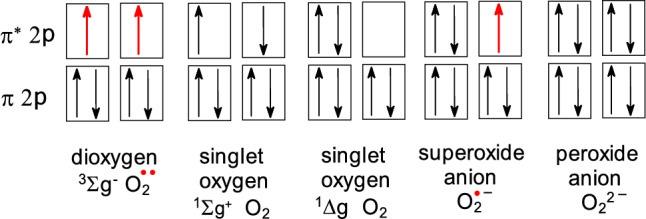
Arrangement of electrons on antibonding π* orbitals of the various forms of molecular oxygen. Radical species containing unpaired electrons are dioxygen and superoxide radical anions. Unpaired electrons are marked as red dots

In addition to triplet oxygen, singlet oxygen can also be formed in biological systems. Singlet oxygen contains the same number of electrons as triplet oxygen; the only difference is that both electrons are paired on antibonding π* orbitals of singlet oxygen. Singlet oxygen is a very efficient oxidizing agent in biological systems and can cause damage to all biomolecules, including DNA. Damaging properties of singlet oxygen, ^1^O_2_ (both electrons paired, *S* = 0, multiplicity 2.*S* + 1 = 2.0 + 1 = 1) can be effectively used in anticancer therapy, involving photodynamic therapy (PDT) (Maharjan and Bhattarai 2022). Addition of one or two electron(s) to molecular oxygen results in the formation of superoxide radical anion (O_2_^·−^) or peroxide (O_2_^2−^), respectively. In contrast to superoxide radical, peroxide is a diamagnetic molecule (does not contain unpaired electron(s), however, due to its oxidizing properties is classified as ROS). Electronic structures of oxygen and oxygen-derived radicals are shown in Fig. 1.

### Superoxide radical anion, hydroperoxyl, and thiyl radicals

The superoxide radical is considered a primary ROS produced in mitochondria by electron leakage from the electron transport chain to molecular oxygen in the course of oxidative phosphorylation (Valko et al. 2004). Recent results using various cell types confirmed the “stochastic and transient” formation of superoxide radicals, termed “mitochondrial” flash from either a single or restricted clusters of interconnected mitochondria (O-Uchi et al. 2018).

The superoxide radical is formed during the infection by activated phagocytes to kill the microorganisms. In in-vitro experiments, superoxide radicals can be generated either enzymatically or non-enzymatically. The superoxide radical is relatively unreactive with biological molecules. Under physiological conditions, a vast majority of superoxide radical is present in the form of the radical anion (O_2_^·−^), and approximately only 0.6% exists in the protonated form as hydroperoxyl radical HOO^·^ (pK_a_ = 4.8); O_2_^·−^ is the conjugate base of a weak acid HOO^·^ (de Grey 2002)

1 $${\text{ HOO}}^{ \cdot } { } \leftrightarrow {\text{ O}}_{2}^{ \cdot - } + {\text{ H}}^{ + }\;\left( {{\text{pK}}_{a} = 4.8} \right)$$

Since superoxide does not react with DNA directly, in the context of DNA damage is of important dismutation reaction of superoxide radical

2 $${\text{H}}^{ + } + {\text{ HOO}}^{ \cdot } { } + {\text{O}}_{2}^{ \cdot - } { } \to {\text{ H}}_{2} {\text{O}}_{2} { } + {\text{ O}}_{2}\;\left( {{\text{k}} = 7 \times 10^{9} {\text{ M}}^{ - 1} {\text{s}}^{ - 1} } \right)$$

Although the concentration of hydroperoxyl radical (HOO^·^) is low, this reaction is significantly faster than the uncatalyzed reaction involving two molecules of superoxide radical

3 $$2{\text{H}}^{ + } + 2{\text{O}}_{2}^{{ \cdot - }} \to {\text{ H}}_{2} {\text{O}}_{2} + {\text{ O}}_{2} \left( {{\text{uncatalyzed}} - {\text{slow}};{\text{ k}} < 0.3{\text{ M}}^{{ - 1}} {\text{s}}^{{ - 1}} } \right)$$

The superoxide anion radicals can be formed in biological systems as a consequence of thiol-mediated repair of radicals (R^·^) by thiols (RSH), resulting in the formation of thiyl radicals (RS^·^) (Valko et al. 2006)

4 $${\text{R}}^{ \cdot } { } + {\text{RSH }} \to {\text{ RH }} + {\text{ RS}}^{ \cdot }\; \left( {\text{thiyl radical}} \right)$$

Thiyl radicals and nucleophilic charged thiolate anion (RS^−^) form disulfide radical anions, (RSSR)^·−^

5 $${\text{RS}}^{ \cdot } { } + {\text{RS}}^{ - } { }\left( {\text{thiolate anion}} \right){ } \leftrightarrow { }({\text{RSSR}})^{ \cdot - }$$

which in turn can reduce molecular oxygen to superoxide anion radical according to the reaction

6 $$({\text{RSSR}})^{ \cdot - } { } + {\text{ O}}_{2} { } \to {\text{ RSSR }} + {\text{ O}}_{2}^{ \cdot - }$$

The above set of reactions, resulting in the formation of superoxide radicals, has been shown to be omnipresent and a critical component in biological systems (Poole 2015; Adams et al. 1969; Davies et al. 2022).

### Hydrogen peroxide

Hydrogen peroxide is an unstable and slowly decomposing peroxide molecule containing an oxygen–oxygen bond. The main source of hydrogen peroxide is formed by SOD-catalyzed superoxide radical dismutation reaction, but it also can be formed by two-electron reduction of oxygen in the reactions catalyzed by oxidases such as xanthine oxidase, glucose oxidase, and others.

Hydrogen peroxide has no charge and therefore, can cross biological membranes. Hydrogen peroxide is a strong oxidant but the kinetics of its reaction with biomolecules is rather slow and therefore it can be accumulated in cells in relatively high concentrations (Andres et al. 2022).

Hydrogen peroxide plays an important role in growth factor-induced signal transduction, maintenance of thiol redox homeostasis, and mitochondrial functioning.

Under the conditions of disturbed redox metal homeostasis (e.g. Alzheimer’s diseases and some cancers), free (unbound) metal ions can catalyze heterolytic cleavage of hydrogen peroxide (formed by the SOD-catalyzed dismutation reaction), resulting in the formation of hydroxyl radicals (^·^OH) and OH^−^. This reaction is termed the Fenton reaction (Valko et al. 2005; Gates 1999)

7 $${\text{H}}_{2} {\text{O}}_{2} + {\text{M}}^{{{\text{n}} + }} \to ^{ \cdot } {\text{OH}} + {\text{OH}}^{ - } {\text{ + M}}^{{\left( {{\text{n}} + 1} \right) + }} \left( {{\text{M}} = {\text{Fe}};{\text{ k}} = 7.6 \times 10^{1} {\text{ M}}^{{ - 1}} {\text{s}}^{{ - 1}} } \right)$$

The efficient formation of hydroxyl radicals is achieved when stoichiometric quantities of redox metals are present and when a reducing agent (O_2_^·−^) that can recycle metal ions back to their active reduced form is present (Valko et al. 2005)

8 $${\text{M}}^{{\left( {{\text{n}} + 1} \right) + }} { } + {\text{ O}}_{2}^{ \cdot - } { } \to {\text{ M}}^{{{\text{n}} + }} + {\text{ O}}_{2} { } \quad \left( {{\text{M}} = {\text{Fe}};{\text{ k}} = 3.1 \times 10^{5} {\text{ M}}^{ - 1} {\text{s}}^{ - 1} } \right)$$

The addition of a hydrogen peroxide-converting enzyme catalase resulted in the suppressed damage to DNA (see also below).

### Hydroxyl and carbonate radicals

**Fig. 2:**
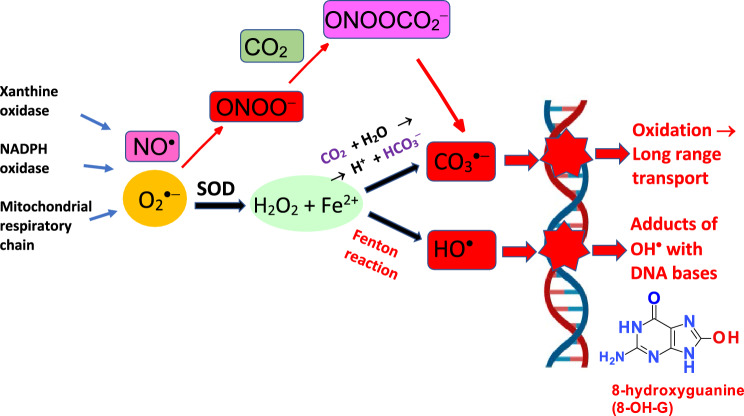
Redox metal-catalyzed Fenton reaction and interaction of redox metals, (copper or iron) with carbonate resulting in the formation of carbonate radical anion (CO_3_^·−^). CO_3_^·−^ can also be formed alternatively starting from the interaction of superoxide radical (O_2_^·−^) and nitric oxide (NO^·^) forming peroxynitrite (ONOO^−^) which in turn reacts with CO_2_ finally forming CO_3_^·−^. Both CO_3_^·−^ and ^·^OH can cause DNA damage

Hydroxyl radical is one of the most reactive radicals occurring in biological systems and causes damage to all important biomolecules involving DNA, proteins, and membranes (Valko et al. 2007). Hydroxyl radicals can be rarely formed as a byproduct of the immune system. When exposed to pathogens, microglia, and macrophages may also form this radical. Hydroxyl radical is one of the most reactive radicals with a short lifetime, about 10^–8^–10^–9^ s. In biological systems, hydroxyl radicals can be formed by homolytic cleavage of water using high-energy irradiation or more probably, by redox metal-catalyzed decomposition of hydrogen peroxide (reaction 7, Fenton reaction). The damaging effect of hydroxyl radicals on all important biomolecules has been implicated in various chronic diseases. In the past 2–3 decades, a detailed qualitative and quantitative identification of various adducts of hydroxyl radical with DNA bases has been reported. The most representative example of such adducts is represented by the reaction of hydroxyl radicals with the DNA base Guanine, resulting in the formation of the 8-OH Guanine adduct (see Fig. 2).

Interestingly, it has recently been reported that the hydroxyl radicals originating from the Fenton reaction might not be the main product when a physiological concentration of bicarbonate is present in the system (Illes et al. 2019). In the course of this reaction, in neutral pH, metals (Fe or Cu) interact with carbonate and hydroperoxides resulting in the formation of carbonate radical anion CO_3_^·−^, which may dominate over the hydroxyl radicals when the concentration of bicarbonate is higher than 100 µM. Alternatively, CO_3_^·−^ can be formed via the reaction between nitric oxide, NO^·^, and superoxide radical anion, O_2_^·−^, forming peroxynitrite reacting further with CO_2_ (see Fig. 2) (Fleming and Burrows 2020; Samuni et al. 2022).

Various experiments have been conducted to study DNA cleavage in the course of the above reactions 7 and 8, involving superoxide anion radicals, traces of transition metals, hydrogen peroxide, and hydroxyl radicals. The addition of catalase, an enzyme converting hydrogen peroxide, into the reaction system, has resulted in a significant decrease in DNA strand scission. It is known, that chelated metals by suitable ligands show due to the decreased number of free metal-binding positions suppressed catalytic activity.

Desferrioxamine (or Desferal) has been shown to decrease DNA cleavage by the Fenton reaction. SOD is another agent which, when present in the reaction mixture, diminished DNA cleavage (Nandi et al. 2019). This can be explained by the fact, that superoxide radical is necessary to achieve redox cycling of metal ions (Halliwell and Gutteridge 1990). The presence of SOD may have no effect, when reducing agents, such as thiols or ascorbic acid, are added to the system. In these cases, the efficiency of DNA cleavage may even increase. When experiments are performed in “hydroxyl radical scavenging” organic solvents such as DMSO or ethanol, diminished DNA cleavage may be expected.

### Peroxyl radicals

Peroxyl radicals (ROO^·^) are formed in the biological systems by the reaction of molecular oxygen with radicals R^·^

9 $${\text{R}}^{ \cdot } { } + {\text{O}}_{2} { } \to {\text{ ROO}}^{ \cdot }$$

Peroxyl radicals and also alkoxyl radicals (RO^·^) are also formed by the decomposition of alkyl peroxides (ROOH)

10 $$2{\text{ROOH }} \to {\text{ ROO}}^{ \cdot } { } + {\text{ RO}}^{ \cdot } { } + {\text{H}}_{2} {\text{O}}$$

**Fig. 3:**
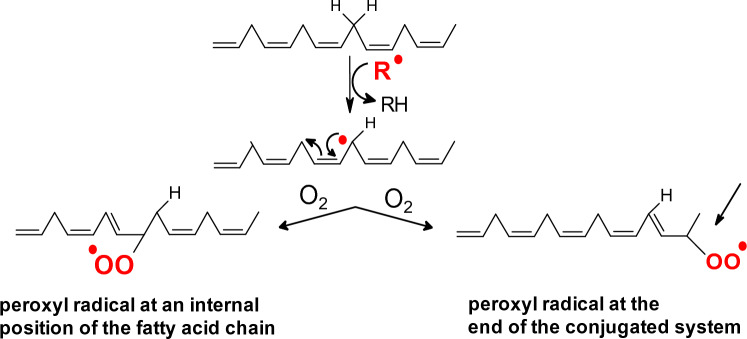
The first steps of the lipid peroxidation pathway

Peroxyl and alkoxyl radicals are strong oxidizing agents and can be formed by the decomposition of peroxides by the traces of redox metals or UV radiation. In biological systems, peroxyl or alkoxyl radicals are formed by the abstraction of bis-allylic hydrogens from polyunsaturated fatty acids (PUFA). This is the first initiation step in the process of peroxidation of lipids (Fig. 3). Peroxyl radicals are relatively long-lived with diffusion ability.

### Singlet oxygen

Singlet radical does not contain unpaired electron(s) and therefore is a diamagnetic ROS. Singlet oxygen has a short half-life, about 10^–6^ s, depending on the media (Valko et al. 2004). Singlet oxygen may react with various cellular components, however, preferentially interacts with conjugated double bonds of polyunsaturated fatty acids. Singlet oxygen may interact with substrates by various mechanisms, the most important being energy transfer or chemical modification.

The damaging properties of singlet oxygen can be effectively used in photo-dynamic therapy (PDT), used for example in cancer treatment, in which singlet oxygen is formed by the light-induced excitation of a suitable photosensitizer (Gunaydin et al. 2021). Formed singlet oxygen along with other ROS may contribute to the damage of cancer cells. PDT therapy is especially suitable for solid tumors.

### Reactive nitrogen species (nitric oxide and peroxynitrite)

Reactive nitrogen species (RNS) are nitrogen-containing species of which the most significant representative example is nitric oxide (NO^·^) (Ignarro et al. 1987). Nitric oxide, the molecule of the Year 1992 by Science Journal, is a small, paramagnetic, and lipid-permeable molecule. Nitric oxide acts as a signaling molecule in the central nervous system. NO^·^ is synthesized by NO synthase (NOS) and forms both covalent and noncovalent linkages with target molecules such as proteins. Covalent attachment of nitric oxide to protein Cysteine results in the formation of S-nitrosothiol (SNO). Nitric oxide reacts very efficiently with superoxide radicals to form peroxynitrite anion ONOO^−^

11 $${\text{NO}}^{ \cdot } { } + {\text{ O}}_{2}^{ \cdot - } {\text{ ONOO}}^{ - }\; \left( {{\text{k}} = \left[ {4 - 16} \right] \times 10^{9} {\text{ M}}^{ - 1} {\text{s}}^{ - 1} } \right)$$

Peroxynitrite is a very powerful oxidant capable of causing damage to biomolecules including proteins and DNA. Peroxynitrite is interfering with signaling pathways by nitrating protein amino acid residues. In addition, peroxynitrite can modify proteins by nitration or oxidation of amino acids, involving cysteine, tyrosine, and methionine (Bartesaghi and Radi 2018). These chemical modifications are responsible for altered chemical and physical properties of proteins. High levels of peroxynitrite anion have been linked with tumor progression. Dysregulation of peroxynitrite has been linked with cardiovascular, neurological, and cancer diseases (Gulcin 2020). Various types of free radicals in biological systems are in Table 1.

**Table 1:** Examples of free radicals in biological systems

Free radicals and non-radical oxidants	Representative examples
Reactive oxygen species (ROS)	Hydroxyl radical (^·^OH), Superoxide radical anion (O_2_^·−^), Peroxyl radical (ROO^·^), Alkoxyl radical (RO^·^), hydrogen peroxide (H_2_O_2_), perhydroxyl radical (HOO^·^), Singlet oxygen (^1^O_2_)
Reactive nitrogen species (RNS)	Nitric oxide (NO^·^), peroxynitrite anion (ONOO^−^), nitrous acid (HNO_2_), nitrogen dioxyde (NO_2_), nitrosyl anion (NO^−^)
Reactive chlorine species (RCS)	Hypochlorous acid (HClO), nitryl chloride (ClNO_2_)
Reactive sulfur species (RSS)	Thiyl radical (RS^·^), hydrogen sulfide (H_2_S), persulfides (i.e., GSSH, CSSH)

### Endogenous sources of ROS

Major sites of ROS production are mitochondria, membrane-bound NADPH oxidases (NOXs), peroxisomes, and chloroplasts in the course of the process of respiration and photosynthesis. Oxidative phosphorylation produces ATP in the course of the electron transfer reactions in the mitochondrial electron transport chain. Production of ATP is tightly linked with the formation of ROS which may be involved in the pathological states of an organism or physiological cell signaling (Valko et al. 2007).

In addition to mitochondria, other cytosol sources of ROS involve cytochrome P450 (CYP) superfamily of heme-thiolate enzymes, that functions as monooxygenases. This system of enzymes is involved in many physiological functions and the detoxification of xenobiotics. P450 uses molecular dioxygen of which one oxygen atom interacts with the substrate and a second oxygen atom, with the use of two electrons provided by NAD(P)H, is reduced to a water molecule. If the transfer of one oxygen to a substrate molecule escapes control mechanisms, uncoupling in the ROS formation and subsequent damage to biomolecules may occur. (Mortezaee 2018).

Another very important source of ROS, including hydrogen peroxide and superoxide anion radicals are dynamic and metabolically active subcellular compartments, peroxisomes (Phaniendra et al. 2015). They play an important role in various metabolic processes such as oxidation of fatty acids, metabolism of ROS, catabolism of purines, prostaglandins, and probably biosynthesis of isoprenoids.

**Fig. 4:**
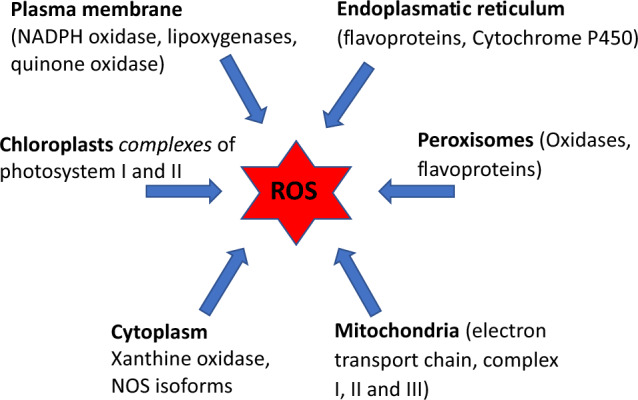
Main cellular sources of ROS formation

The endoplasmic reticulum (ER) is a complex dynamic structure possessing many functions in the cell, the most important ones include calcium storage, lipid metabolism, and protein synthesis (Cao et al. 2022). Different ER domains consisting of tubules, sheets, and nuclear envelopes are responsible for various functions. It is believed that the redox homeostasis in the ER may result in ER-associated stress, which may trigger redox signaling mediators playing roles in ROS formation and consequently in the pathogenesis of various human disorders. Figure 4 shows the main cellular sources of ROS.

### Exogenous sources of ROS

Exogenous sources of ROS involve radiation, anticancer therapy, smoking, consumption of alcohol, and certain drugs. Exogenous sources of ROS also involve the consumption of smoked meat and intake of metal-containing drugs (cyclosporine, bleomycin, gentamycin, and others) (Bhattacharyya et al. 2014).

Both anticancer and radiation therapies are responsible for the formation of ROS; especially certain ROS-induced cardiovascular complications may result as a consequence of such therapies. It has been well established that the X-rays, α, β, and γ rays, and bursts of free neutrons may cause the formation of ROS and consequently oxidative stress. Ionizing radiation may cause radiolysis of water, accompanied by the formation of hydroxyl radicals (Nuszkiewicz et al. 2020).

Chemotherapeutic drugs are known to have toxic side effects accompanied by ROS formation, documented by the increased peroxidation of lipids and suppressed levels of antioxidants such as glutathione and low molecular weight antioxidants. Drugs inducing high levels of ROS involve camptothecins, anthracyclines (doxorubicin, daunorubicin), Pt-based drugs, and others (Brezova et al. 2003).

Smoking is a significant source of ROS and has been documented to affect the gastrointestinal tract. It has been estimated that one puff of cigarette smoke contains approximately 10^15^ free radicals of various origins (Yang et al. 2008). Smoke substances involve aldehydes, quinones, epoxides, nitric oxides, and others. GI problems involve Crohn’s disease, reflux, esophagus cancer, and other disorders (Bhattacharyya et al. 2014).

A positive correlation between the high consumption of red meat and the incidence of colon cancer has been proposed (Valko et al. 2001). Red meat contains high content of iron which may catalyze ROS formation in the colon in the process of digestion (Valko et al. 2001). Trans fatty acids are also a source of ROS due to the presence of acrylamide, occurring in snacks, cereals, and crackers. Acrylamide can interact with hemoglobin and increase ROS formation (Bergmark et al. 1993).

Ethanol, especially spirits, in high amounts can damage the mucosal layer of the gastrointestinal tract. Liver diseases related to alcohol consumption are partly due to the ROS formation from ethanol (Wu and Cederbaum 2009).

## ROS-mediated cell signaling

As outlined above, ROS have origin from two main sources: mitochondrial oxidative metabolism and processes of cellular response to xenobiotics, bacteria, and cytokines during which ROS are formed as a part of either signal transduction pathways or a cellular defense mechanism (Finkel 2011). Superoxide radical anion (O_2_^·−^) is considered the initial ROS formed by complexes I and III of the mitochondrial respiratory chain. The superoxide radical is dismutated to hydrogen peroxide by SOD and then to water and oxygen by catalase or glutathione peroxidase. NADPH oxidases (NOX) are another source of cellular ROS.

### Nuclear factor-κB

The nuclear factor-κB (NF-κB) is an important inducible transcription factor playing a key role in processes of the immune and inflammatory responses, cellular adhesion, differentiation, proliferation, and apoptosis (Bonizzi and Karin 2004). Deregulated NF-κB activation has been associated with cancer, neurodegenerative disorders, inflammatory diseases, and arthritis. The family of NF-κB consists of five structurally similar members, involving p50/p105 (NF-κB1), p52/p100 (NF-κB2), RelA (p65), RelB, and c-Rel (Liu et al. 2017).

NF-κB can be activated by two major canonical and noncanonical pathways. The canonical activation pathway is triggered through the site-specific phosphorylation by an IκB-kinase (IKK) complex in response to microbes, and proinflammatory cytokines. NF-κB translocated into the nucleus activates the transcription of target genes (Basak and Hoffmann 2008). In contrast to the canonical pathway, the non-canonical NF-κB activation does not involve IκBα degradation but is dependent on the p100 (NF-κB2) precursor protein. The non-canonical NF-κB pathway is activated by a specific group of stimuli, such as CD40 ligand, CD27 ligand, BAFF, human T-cell leukemia virus, and other stimuli.

ROS activates NF-κB by inhibiting the phosphorylation of IκBα. In addition, I-κB-Kinase (IKK) complex is the core component of NF-κB and is a target of ROS, affecting thus NF-κB and S-glutathionylation and inhibited IKKβ activity (Reynaert et al. 2006).

Ubiquitination plays a key role in the regulation of the NF-κB pathway (Chen and Chen 2013). NF-κB pathway can enhance the expression of antioxidant enzymes such as superoxide dismutases (SODs) glutathione peroxidase (GPx) and other enzymes.

### Mitogen-activated protein kinase

The mitogen-activated protein kinase (MAPK) cascades are major intracellular signaling pathways that regulate various cellular processes, such as proliferation, differentiation, and stress response. Four main MAPK cascades have been identified: ERK1/2 (extracellular signal-regulated kinases), c-Jun N-terminal kinases (JNK), p38 (p38 kinase), and the BMK1/ERK5 (big MAP kinase 1) (Schaeffer and Weber 1999).

ERK plays important roles in the processes involving cell growth, cell proliferation, and cell survival (Lavoie et al. 2020). ERK activation is achieved by the stimulation of tyrosine kinase receptors and pathways activated by the various growth factors and cytokines. ROS have been documented to activate receptors of two main growth factors, EGF (epidermal growth factor) and PDGF (platelet-derived growth factor). JNK can also be activated by ROS, through their interaction with redox-active proteins such as thioredoxin (TRX) and glutaredoxin.

Apoptosis signal-regulating kinase 1 (ASK1) is a serine/threonine protein kinase and activates JNK and p38 pathways. The thioredoxin-ASK1 complex plays also a role in the signaling of tumor necrosis factor α (TNFα). It has been reported that ROS stimulated the oxidation of thioredoxin and its dissociation from ASK1 (Matsukawa et al. 2004). TNFα-mediated JNK activation is believed to be mediated by ROS (superoxide radical anion and peroxyl radicals) because their specific scavengers have been shown to inhibit the activation of JNK.

P38 is a major pathway influencing cell proliferation, cell cycle, and apoptosis, and is activated in response to various environmental stresses, involving inflammatory cytokines, oxidative and nitrosative stress, and DNA damage (Shahab and Jamesdaniel 2022; Zhang et al. 2016).

The BMK1/ERK5 is responsible for cell survival, differentiation and proliferation, angiogenesis, and antiapoptotic signaling. It has been confirmed that hydrogen peroxide may influence the BMK1 pathway through the direct activation of MEKK2 and MEKK3 (Zhang et al. 2016).

### Phosphoinositide-3-kinase

The phosphatidylinositol 3-kinase (PI3K)/protein kinase B (AKT) is an intracellular pathway involved in the regulation of a variety of critical cellular physiological processes involved in the cell cycle growth and proliferation and apoptosis, protein synthesis, and drug resistance in response to stimulation by growth factors, cytokines and hormones (Leslie and Downes 2002). ROS are known to activate PI3K directly to enhance downstream signaling. However, ROS can also inhibit the activation of Akt by oxidizing Cys-residues in the active center.

Protein phosphatase 2 (PP2A) is a heterotrimeric protein with serine/threonine phosphatase activity targeted to oncogenic signaling cascades, involving also Akt, where PP2A acts as a tumor suppressor. PP2A can be deactivated by ROS which may in turn inhibit Akt/PKB. Low levels of ROS may oxidize the –S–S– bridges of Akt/PKB, promoting the interaction of Akt/PKB with PP2A. (Lee et al. 2002).

### Calcium

Divalent calcium ions (Ca^2+^) are involved in many aspects of cellular life and death. Ca^2+^ signaling is important in secretion, metabolism, cell survival, and death (Tarasov et al. 2012). A very large gradient (≈ 10,000) of calcium ions exists across the plasma membrane (Jomova et al. 2022). Intracellular stores of Ca^2+^ regulate cellular redox equilibrium by ROS formation and elimination, allowing cells to shift the redox environment towards either a more oxidizing or reducing state. The key role of Ca^2+^ is ATP synthesis and mitochondrial ROS formation through oxidative phosphorylation and activation of enzymes (Lewis et al. 2014). Mitochondrial Ca^2+^ activates three dehydrogenases of the citric acid cycle resulting in increased ROS formation (Hatano et al. 2013). Ca^2+^-regulated extramitochondrial enzymes, such as NADPH oxidases and nitric oxide synthase (NOS) under both physiological and pathological conditions, stimulate ROS formation (Roe et al. 2013).

Ca^2+^ can directly activate antioxidant enzymes such as GSH reductase and catalase and SOD through which ROS clearance is mediated (Thompson et al. 2014). In addition, ROS mediates the oxidation of Cysteine thiols of a family of Ca^2+^ transporters. Generally, the oxidation of thiols by ROS enhances channel activity and promotes the efflux of Ca^2+^. ROS are also known to modulate the activity of voltage-dependent Ca^2+^ channels, the most affected being the activity of L-type Calcium channels. L-type channels are linked with the oxidation of thiol groups resulting in changed entry of calcium in the cytoplasm (Nishida et al. 2013).

### Protein kinases

The protein kinase family is a very large group of kinases (in men more than 500) catalyzing the phosphorylation of proteins. Their role in the control of various cellular functions, involving proliferation, apoptosis, metabolism, and others is of great importance (Elkoshi 2021). Disturbed regulation of protein kinases is linked with the appearance of many cancers, such as breast, pancreatic, lung, and other cancers as well as autoimmune diseases such as rheumatoid arthritis and psoriasis.

ROS oxidize -SH groups of cysteine residues in protein kinases A, C, and D, receptor tyrosine kinase (RTK), and Ca/calmodulin independent protein kinase II, which in turn phosphorylate target proteins participating in various signaling mechanisms (Kruk et al. 2013). ROS are known to affect phosphorylation of protein kinase A which in turn influences ROS equilibrium. While low levels of ROS can stimulate the activity of protein kinase C, high ROS levels may oxidize critical cysteine residues which are manifested by the suppressed activity. For example, hydrogen peroxide was able to regulate the phosphorylation of 512 and 523 tyrosine residues of protein kinase C (Aicart-Ramos et al. 2014). Similarly to protein kinase C, also kinase D is activated by ROS through the phosphorylation of tyrosine 463 residue. Low to intermediate levels of ROS have been shown to activate both, receptor tyrosine kinase and Ca/calmodulin, the latter by the oxidation of Methionine 281 and 282 residues.

## Antioxidant systems

Approximately 4–5% of oxygen in the human body is converted to ROS by biological reductants. Cells under a higher partial pressure of oxygen are permanently exposed to attack by ROS which is balanced by the concerted action of a sophisticated antioxidant defense system (Bandyopadhyay et al. 1999). Antioxidants are generally divided into antioxidant enzymes and low molecular weight antioxidants. The most important antioxidant enzymes involve Superoxide dismutases (SODs), Catalase (CAT), Glutathione peroxidase (GPx), and others. The most important low molecular weight antioxidants involve vitamin C, vitamin E, carotenoids, flavonoids, glutathione, and other antioxidants.

### Antioxidant enzymes

Antioxidant enzymes are a class of proteins/metalloproteins, that catalyze the transformation of ROS and/or their byproducts into more stable, usually less harmful species. Antioxidant enzymes represent an important defense mechanism against ROS-induced oxidative stress, causing damage to all cellular components (Mates et al. 1999).

Some enzymes, such as superoxide dismutase, glutathione peroxidase and catalase act in a mutually supporting defense action against ROS (Krishnamurthy and Wadhwani 2012).

#### Superoxide dismutase (SOD)

SOD was discovered in 1967 by Irwin Fridovich (McCord and Fridovich 1969). There are three main forms of superoxide dismutase present in humans, Cu, Zn-SOD (SOD1), Mn-SOD (SOD2), and EC-SOD (SOD3) (Zelko et al. 2002).

Cu, Zn-SOD (SOD1) is a homodimer and contains copper and zinc ions. SOD is located in the cytoplasm and is considered one of the most effective enzymes. This enzyme catalyzes the dismutation of superoxide anion radical very effectively by a “Ping Pong” mechanism with remarkably high reaction rates according to the reaction (Zelko et al. 2002)

12 $$2{\text{H}}^{ + } + 2{\text{O}}_{2} ^{{ \cdot - }} \xrightarrow{{{\text{SOD}}}}{\text{H}}_{2} {\text{O}}_{2} + {\text{O}}_{2} \;\left( {{\text{fast}};\;k \sim 2 \times 10^{9} {\text{M}}^{{ - 1}} {\text{s}}^{{ - 1}} } \right)$$

All SODs bind negatively charged anions, such as fluorides, and azides, however, with different affinities. SODs can be inhibited also by copper chelators such as EDTA and cuprizone (a copper‐specific chelator) (Zhu et al. 2014).

Mn-SOD (SOD2) is a homotetrameric enzyme containing Mn in its active center. It is localized in the mitochondria and constitutes cca 15% of the total superoxide dismutases. In the course of the dismutation reaction, the Mn ion is cycling from a more stable Mn(III) oxidation state to a less stable Mn(II). Mn-SOD gene expression is induced by various stimuli, including inflammatory cytokines, fluctuations in redox equilibrium, ionizing radiation, and even cigarette smoke.

Extracellular superoxide dismutase (EC-SOD, SOD3) is the major extracellular SOD found predominantly in the extracellular matrix of tissues. SOD3 is expressed mainly in the lungs. Similarly to SOD1 and SOD2, also SOD3 is involved in the detoxification of ROS by the catalytic dismutation of superoxide radical anion. EC-SOD has an affinity towards heparin and regulates levels of NO^·^. EC-SOD and glutathione peroxidase represent an important first line of defense against oxidants (Rahman and Biswas 2006).

#### Catalase

Catalase is one of the most important antioxidant enzymes occurring in aerobic organisms. It has been isolated from both eukaryotic and prokaryotic organisms. Catalase breaks down two molecules of hydrogen peroxide into two water molecules and one molecule of oxygen (Nandi et al. 2019)

13 $${\text{2H}}_{{2}} {\text{O}}_{2} \mathop{\longrightarrow}\limits^{{{\text{Catalase}}}}2{\text{H}}_{{2}} {\text{O + O}}_{{2}}$$

The reaction proceeds in two steps; the first step involves the reduction of the first molecule of H_2_O_2_ accompanied by the formation of oxyferryl species (Fe^IV^O) and in the course of the second step, the Fe^IV^O complex is reduced by the second molecule of H_2_O_2_ to O_2_, and H_2_O molecules and free enzyme (Lardinois 1995; Nandi et al. 2019)

14 $$\mathrm{Catalase}\left[\mathrm{Fe}\left(\mathrm{III}\right)\left(\mathrm{porphyrin}\right)\right] + {\mathrm{H}}_{2}{\mathrm{O}}_{2} \to \mathrm{ Catalase}\left[\mathrm{Fe}\left(\mathrm{IV}\right)-\mathrm{O}\left(\mathrm{porphyrin}\right)\right]+ {\mathrm{H}}_{2}\mathrm{O}$$

and

15 $$\mathrm{Catalase}\left[\mathrm{Fe}\left(\mathrm{IV}\right)-\mathrm{O}\left(\mathrm{porphyrin}\right)\right] + {\mathrm{H}}_{2}{\mathrm{O}}_{2} \to \mathrm{ Catalase}\left[\mathrm{Fe}\left(\mathrm{III}\right)\left(\mathrm{porphyrin}\right)\right]+ {\mathrm{H}}_{2}\mathrm{O }+ {\mathrm{O}}_{2}$$

Catalase plays an important role in oxidative stress-related conditions, involving inflammation, mutagenesis, and apoptosis suppression (Sandstrom and Buttke 1993). Catalase deficiency is linked with many diseases, such as neurological disorders (Alzheimer’s disease, Parkinson’s disease, schizophrenia, and bipolar disorder), metabolic diseases (diabetes I and II, hypertension, insulin resistance), and cancer, anemia, and asthma. Catalase has potential in the treatment of oxidative stress-related diseases; this medical approach needs further research.

#### Glutathione peroxidase

Glutathione peroxidase (GPx) is a family of enzymes containing a selenocysteine residue at the active site and possessing peroxidase activity. GPx reduces lipid hydroperoxides to alcohols and hydrogen peroxide to water (Lubos et al. 2011). GPx family consists of several isoenzymes of which Glutathione peroxidase-1 (GPx-1) is the most abundant and whose substrate is hydrogen peroxide. GPx-1 acts in the intracellular environment and maintains the physiological level of hydrogen peroxide, which is important in mitochondrial function, thiol redox homeostasis, and signal transduction (Brigelius-Flohé and Maiorino 2013).

The net reaction of the inactivation of peroxides (hydrogen peroxide) is described by the reaction

16 $${\text{ROOH }} + { }2{\text{GSH }}\mathop{\longrightarrow}\limits^{{{\text{GPx}}}}{\text{ ROH }} + {\text{ GSSG }} + {\text{ H}}_{2} {\text{O}}$$

where GSH is reduced and GSSG is oxidized glutathione, respectively.

**Fig. 5:**
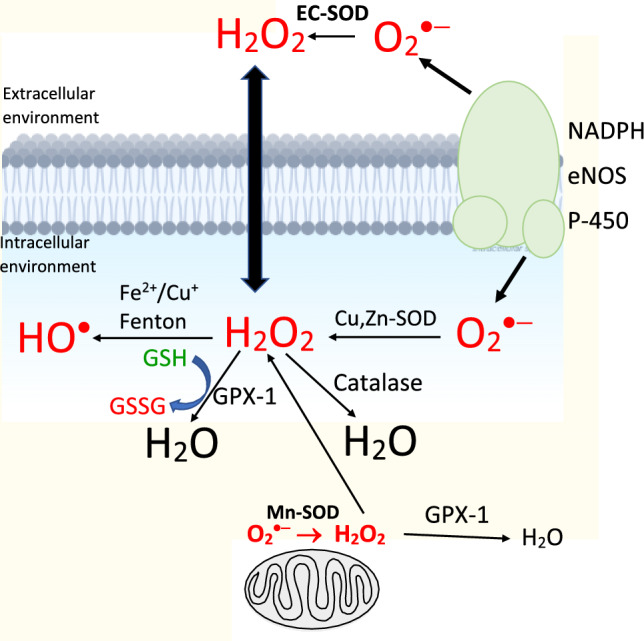
Concerted action of Glutathione peroxidase-1, Catalase, and Superoxide dismutase

Various ROS and RNS can inactivate the functioning of GPx-1, involving superoxide radicals, peroxynitrite, and nitric oxide, formed for example by inducible NO synthase. However, the degree of inactivation depends on the intracellular redox state, the most critical is the concentration of reduced glutathione GSH, and the GSH/GSSG ratio. GPX-1 prevents the formation of DNA mutations and suppresses carcinogenic potential. Excessive depletion of oxidants may result in the induction of reductive stress, characterized by declined physiological functions, such as suppressed mitochondrial function, cell proliferation, the occurrence of cardiomyopathies, and other pathologies (Rajasekaran et al. 2007). The mutual interplay between Glutathione peroxidase-1, Catalase, and Superoxide dismutase is shown in Fig. 5.

#### Antioxidant enzyme-mimetics

Superoxide dismutases (SODs), catalase (CAT), and glutathione peroxidases (GPxs) are the most important antioxidant enzymes playing key roles in redox homeostasis. These properties make them attractive biomolecules for the design of low molecular weight metallo-complexes with ROS-scavenging properties under pathological conditions.

Perhaps the most frequently studied were metallo-complexes possessing SOD mimetic (superoxide radical scavenging) properties. Manganese is a transition metal capable of oscillating between several oxidation states which makes it suitable for the accommodation of superoxide radical anion and the design of SOD mimetic compounds (Vincent et al. 2021). To date, a great number of Mn-based complexes have been synthesized and studied for their superoxide-scavenging properties. The major advantage of Mn-complexes over Cu-complexes is their limited catalytic activity in the Fenton reaction which would otherwise increase the level of ROS (hydroxyl radical) and consequently oxidative stress. The frequently studied ligand environments around the Mn center were salen derivatives, nitrogen-centered ligands, cyclic polyamines, carboxylate/ amino-carboxylate ligands, porphyrins, peptides, phthalocyanines and other ligands (Forman and Zhang 2021). Several Mn(II)-based cyclic polyamine and Mn(III) porphyrins have entered several clinical trials (Batinic-Haberle and Tome 2019).

Several SOD mimetic compounds exhibit simultaneously SOD and catalase mimetic properties. For example, the Mn-salen complex (EUK-8) exhibits both SOD and catalase mimetic properties and has been found to ameliorate sepsis- or lipopolysaccharide-induced pulmonary dysfunction (Gonzales et al. 1995). SOD and catalase mimetic compounds naturally exhibit several orders of magnitude lower rate constants than their corresponding enzymes, however, their use in extracellular environments, where concentrations of enzymes are rather low, is of significant importance. It has been demonstrated that some mimetic metallo-complexes may be effective in the mitochondrial matrix, however, due to the relatively easy change of the oxidation state of a metal center, their potential prooxidant properties have to be considered.

Various glutathione peroxidase mimetic compounds have been prepared and tested. These involve among others, the Se-based organic compound ebselen, possessing a wide substrate specificity (Sies 1993a). This compound and its modified analog BXT-51072 were able to delay neurological deficits, and acute ischemic stroke, inhibit the inflammatory response, suppress oxidative damage, and other beneficial oxidative stress-reducing properties (Forman and Zhang 2021).

### Chemistry of low molecular weight antioxidants

Low molecular weight antioxidants represent an important line of cellular defense mechanisms against oxidants (Pinchuk et al. 2021). As discussed above, enzymatic antioxidants effectively convert by a multistep mechanism oxidized metabolic products to hydrogen peroxide and water. Low molecular weight antioxidants terminate radical chain reactions. Some of the low molecular weight antioxidants are water soluble and operate in the cytosol or cytoplasmic matrix. Another group of antioxidants, soluble in lipids, operates in the membranes. The most representative examples of small molecular antioxidants involve vitamins C, E, and A, polyphenols of which about half are flavonoids, carotenoids, glutathione, curcumin, melatonin, and others (Niki 2016).

#### Vitamin C

**Fig. 6:**
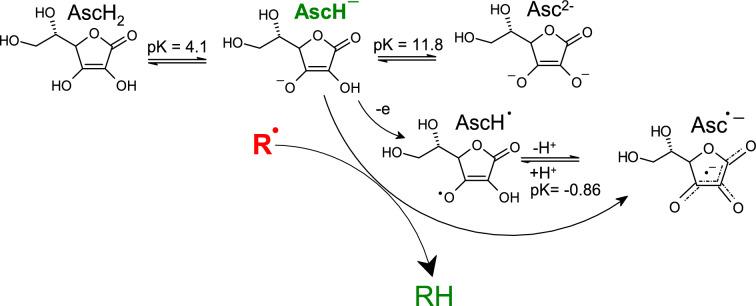
Radical scavenging activity of vitamin C (ascorbic acid). Vitamin C is present under physiological conditions predominantly in the form of an ascorbate anion, AscH^−^. Following its reaction with radical R^·^, the hydrogen atom is abstracted from AscH^−^, and radical AscH^·^ is formed. AscH^·^ is afterward transformed to a more stable ascorbyl radical Asc^·−^

Vitamin C (ascorbic acid) is a water-soluble antioxidant present in the diet, supplements, and preservatives (Kazmierczak-Baranska et al. 2020). Antioxidant (radical scavenging) properties of vitamin C under in vitro conditions can be described by the following reaction scheme (Fig. 6).

The chemistry of vitamin C under physiological conditions is the chemistry of ascorbate anion AscH^−^. The reaction of radical R^·^ with AscH^−^ results in the formation of ascorbyl radical (Asc^·−^), considered a terminal ROS in biological systems with a very low damaging effect. This radical can be detected in biological fluids/tissues using DMPO spin trapping EPR spectroscopy; the EPR signal is recorded as a doublet. Vitamin C predominantly acts as an antioxidant. However, under certain conditions, in the presence of free (unbound) redox metals, such as iron or copper, resulting from metal dyshomeostasis, vitamin C may exhibit prooxidant properties

17 $${\text{AscH}}^{ - } { } + {\text{ Fe}}^{3 + } { } \to {\text{ Asc}}^{ \cdot - } { } + {\text{ Fe}}^{2 + } { } + {\text{ H}}^{ + } { }$$

18 $${\text{ Fe}}^{2 + } + {\text{ H}}_{2} {\text{O}}_{2} { } \to {\text{ Fe}}^{3 + } + { }^{ \cdot } {\text{OH }} + {\text{ OH}}^{ - }\;\left( {\text{Fenton reaction}} \right)$$

19 $${\text{Fe}}^{2 + } + {\text{ O}}_{2} { } \to {\text{ Fe}}^{3 + } + {\text{ O}}_{2}^{ \cdot - }$$

Prooxidant properties of vitamin C in the presence of redox-active metal ions and oxygen have been confirmed predominantly under in vitro conditions, occurrence under in vivo conditions has not been convincingly documented (Halliwell 1996). This may be due to the fact, that homeostasis of redox metals is tightly controlled and free metals (e.g. labile iron pool) are present at very low concentrations due to the metal sequestration. Ascorbate has the ability, under in vitro conditions, to regenerate Vitamin E from its radical, tocopheryl form (see below).

#### Vitamin E

**Fig. 7:**
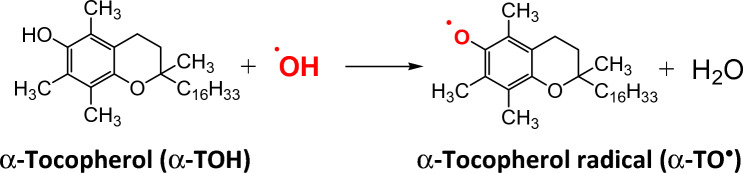
Reaction of vitamin E (α-TOH) with hydroxyl radical resulting in the formation of radical of vitamin E, tocopheryl radical (α-TO^·^)

Vitamin E (α-Tocopherol) is a fat-soluble vitamin protecting biological membranes against ROS-induced peroxidation of lipids. Deficiency of Vitamin E is related to neurological problems (National Institutes of Health 2021). Vitamin E is anchored in a lipid membrane with the hydroxyl group on its six-membered ring located outside of the membrane towards the water environment scavenging ROS. The reaction of α-Tocopherol (α-TOH) with radicals (e.g. ^·^OH) results in the formation of α-Tocopheryl radical (α-TO^·^) (Fig. 7).

Since the standard one-electron reduction potential of (α-TO^·^/α-TOH) couple is about 500 mV and (Asc^·−^/AscH^−^) couple is 280 mV, vitamin C can regenerate vitamin E (α-TOH) from its radical form (α-TO^·^) according to the reaction

20 $${ }\alpha {\text{TO}}^{ \cdot } { } + {\text{ AscH}}^{ - } { } \to { }\alpha {\text{TOH }} + {\text{ Asc}}^{ \cdot - }$$

21 $${\text{ E}}^{\Theta } \left( {\frac{{{\text{Asc}}^{ \cdot - } }}{{{\text{AscH}}^{ - } }}} \right) = 280{\text{ mV}};{\text{ E}}^{\Theta } \left( {\frac{{\alpha {\text{TO}}^{ \cdot } }}{{\alpha {\text{TOH}}}}} \right) = 500{\text{ mV}}$$

#### Glutathione

**Fig. 8:**
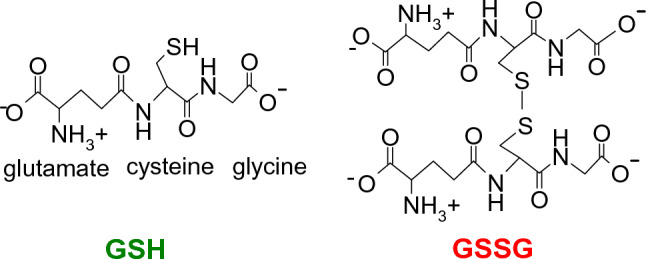
Structures of reduced (GSH) and oxidized (GSSG) glutathione

Tripeptide Glutathione (GSH) is a major multifunctional cellular thiol antioxidant and redox buffer. The abundance of GSH is unusually high, the cytosol concentration is ca 1–11 mM, mitochondria contain 6–10 mM, and nuclei 3–15 mM of GSH. The oxidized form of glutathione is glutathione disulfide, GSSG (Fig. 8) (Lushchak 2012; Valko et al. 2006).

**Fig. 9:**
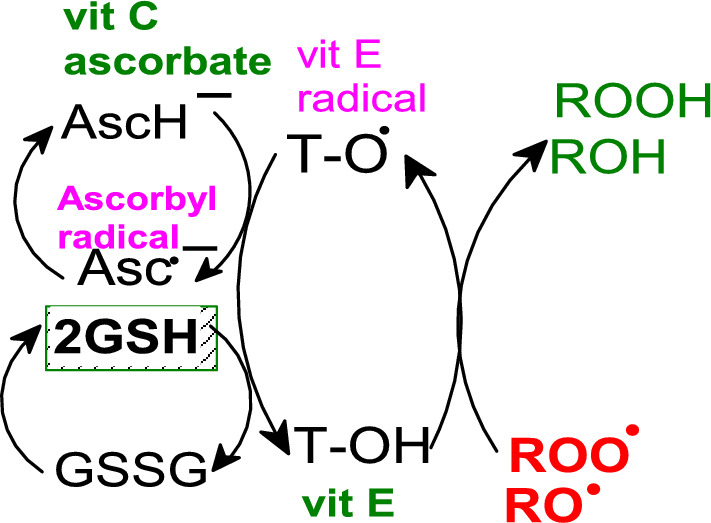
Antioxidant network of the regeneration of vitamin E by vitamin C and glutathione (GSH). Standard one-electron reduction potentials increase in the order: E^Θ^ (GSSG/GSH) =  − 264 mV, *E*^Θ^ (Asc^·−^, H^+^/AscH^−^) = 282 mV; *E*^Θ^ (α-TO^·^, H^+^/α-TOH) = 500 mV at pH = 7.4. Antioxidants with lower reduction potentials can regenerate antioxidants with higher reduction potentials

GSH scavenges a wide range of ROS (^·^OH, H_2_O_2_, ^1^O_2_), and due to the very low value of electron reduction potential of the GSSG/GSH redox couple (*E*^Θ^ = − 264 mV at pH = 7.4) is GSH able to regenerate several important antioxidants, such as radicals of vitamins E and C back to their active non-radical forms (Jones 2002) (Fig. 9).

**Fig. 10:**
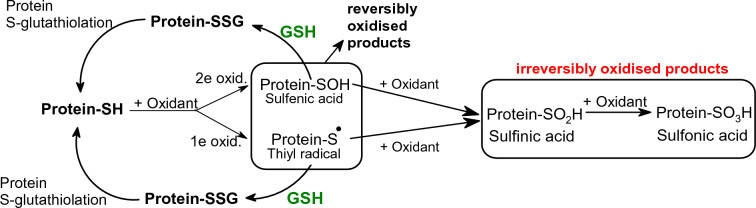
Protective role of GSH in the oxidation of protein -SH groups (Adapted from Valko et al. 2006)

Protein sulfhydryl groups (-SH) are important cellular redox buffers necessary for the expression and repair of DNA. An effect of one electron oxidation by oxidants may result in the modification of protein-SH groups and consequently the formation of thiyl radicals (Protein-S^·^) and protein sulfenic acid (Protein-SOH) (Valko et al. 2006). These two oxidized products can be reduced back to Protein-SH by the effect of GSH via the intermediate formation of S-glutathiolated protein (Protein-SSG). If the process of oxidation is not reversed at this stage, further oxidation results in the formation of irreversibly oxidized products such as sulfinic and sulfonic acids (Fig. 10).

Compounds containing sulfur are usually good antioxidants able to accommodate unpaired electron(s), which in turn result in the formation of thiyl radicals (GS^·^), whose lifetime is rather high and therefore damage to biological molecules is very mild (Karoui et al. 1996)

22 $${\text{GSH }} + {\text{ R}}^{ \cdot } { } \to {\text{ GS}}^{ \cdot } { } + {\text{ RH}}$$

Two thiyl radicals can combine and form a non-radical oxidized glutathione (GSSG)

23 $${\text{GS}}^{ \cdot } { } + {\text{ GS}}^{ \cdot } { } \to {\text{ GSSG}}$$

The GSH/GSSG ratio is a good marker of oxidative stress of an organism (Hwang et al. 1992).

#### Carotenoids

**Fig. 11:**
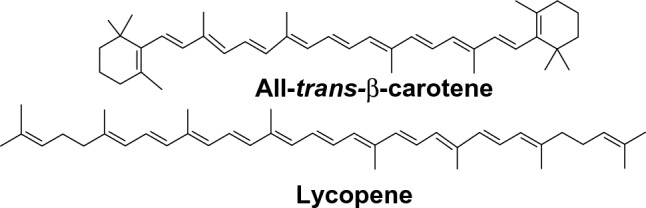
Examples of carotenoid structures

Carotenoids (Car) are red, orange, and yellow pigments that are found in plants, bacteria, and algae (Fig. 11). Over 1000 carotenoids have been identified in nature. Many of them exhibit health-beneficial properties in the prevention of cancer, macular degeneration, atherosclerosis, age-related muscular degeneration, and other diseases.

The antioxidant properties of carotenoids are related to conjugated double bonds which can accommodate unpaired electrons and extensively delocalize them over the conjugated system of double bonds (Mortensen et al. 2001). Carotenoids are effective in scavenging peroxyl (ROO^·^), alkoxyl (RO^·^), hydroxyl (^·^OH), and superoxide anion radicals (O_2_^·–^). The main protective antioxidant activity of carotenoids is their protection of membranes against ROS-induced damage.

There are several mechanisms by which carotenoids can terminate ROS. The most frequent involve (a) hydrogen abstraction from the carotenoid molecule, (b) electron transfer reaction, and (c) radical addition to carotenoid structure (El-Agamey et al. 2004).

Like many other antioxidants, also carotenoids may under certain conditions behave as prooxidants. The first who proposed prooxidant properties of carotenoids were Burton and Ingold (Burton and Ingold 1984). This hypothesis has been confirmed later by the Alpha-Tocopherol/Beta-Carotene (ATBC) trial in Finland organized by the National Cancer Institute NIH (ATBC Trial 1996). This trial aimed to evaluate the antioxidant vs prooxidant properties of β-carotene. In this trial, a large cohort was supplemented with β-carotene (20 mg/day) for a period of 5–8 years. The results of the trial were very surprising. Concerning to the control group, there was an 18% increased incidence of lung cancer among those taking β-carotene supplements.

The key factor affecting the switch from antioxidant to prooxidant properties of carotenoids has been attributed to the partial pressure of oxygen, typically high in the lungs. Under conditions of high partial pressure of oxygen, radicals of carotenoids, Car^·^, originating from the reaction of ROS with a carotenoid molecule by hydrogen abstraction may further react with dioxygen according to the reaction (Young and Lowe 2001; Shin et al. 2020)

24 $${\text{Car}}^{ \cdot } { } + {\text{ O}}_{2} { } \to {\text{ CarOO}}^{ \cdot }$$

Car-OO^·^ may behave as a prooxidant by further reaction with unsaturated lipids

25 $${\text{CarOO}}^{ \cdot } { } + {\text{ RH }} \to {\text{ CarOOH }} + {\text{R}}^{ \cdot }$$

Thus carotenoids behave under normal conditions as antioxidants, however, under a high partial pressure of oxygen (and high concentration) they may lose their antioxidant properties and behave as prooxidants.

#### Flavonoids

**Fig. 12:**
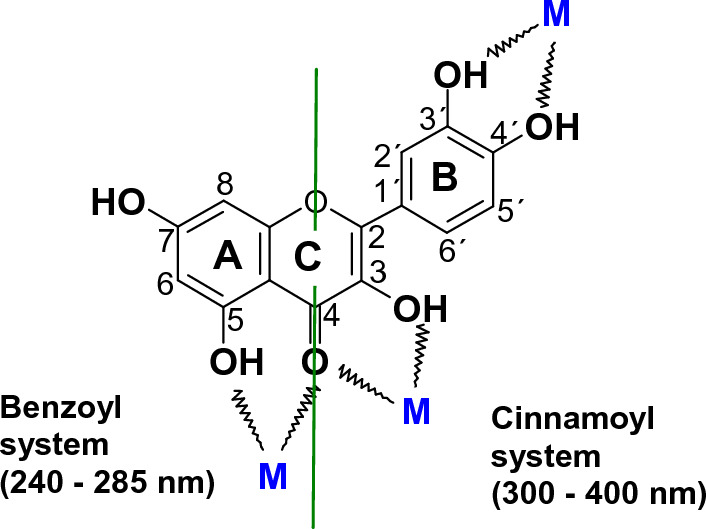
Structure of a flavonoid quercetin and absorption bands of the benzoyl and cinnamoyl systems. (M = chelated metal ion)

The health-beneficial effects of flavonoids in the prevention of chronic diseases are predominantly related to their antioxidant properties manifested by the ability to terminate free radicals and effectively chelate redox metal ions. In addition, flavonoids can activate the synthesis of antioxidant enzymes and regenerate vitamins with higher electron reduction potential of the corresponding couples, such as vitamin E (Fig. 12) (Simunkova et al. 2019a, b).

UV–vis spectra of flavonoids exhibit two major absorption maxima: band II, corresponding to the benzoyl system of ring A (240–285 nm), and the band I, corresponding to the cinnamoyl system of the B ring (300–400 nm) (Fig. 12). Flavanones have a saturated C ring, lacking conjugation between both A and B rings. This structural characteristic is reflected by an intense maximum spectral Band II and only a weak shoulder for Band I. Due to the lack of conjugation, flavanones usually have lower antioxidant activity (Jomova et al. 2019).

**Fig. 13:**
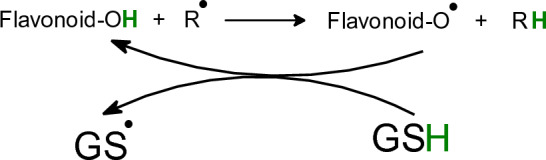
Interaction of flavonoids with radical R^·^ and regeneration of flavonoid radicals by glutathione (GSH)

Antioxidant properties of flavonoids predominantly depend on the number and position of hydroxyl groups and their metal-chelating ability (Fig. 12) (Lomozova et al. 2022; Simunkova et al. 2021). Hydroxyl groups of flavonoids are directly involved in the scavenging activity of radicals, forming flavonoid radicals that can be regenerated by Glutathione (GSH) (Fig. 13). B ring of flavonoids is very important in scavenging radicals, most frequently via hydrogen-atom and/or electron transfer to hydroxyl, peroxyl, and other radical species. A linear correlation between the reduction potentials of flavonoids and their antioxidant potential has been reported (Lindgerg Madsen et al. 2000). The antioxidant activity of flavonoids depends on the availability of the donation of phenolic hydrogens (Rice-Evans et al. 1996). To be an efficient antioxidant, flavonoid molecules should fulfill two criteria: (i) flavonoid molecules, present at low concentration with respect to the substrate to be oxidized, should prevent or delay free radical-mediated oxidation; (ii) the terminal radical formed, should exhibit a lower degree of reactivity. An important structural feature of flavonoids is the presence of a 2,3-unsaturated double bond in the C ring, which allows electron delocalization within the molecule and stabilizes aryloxyl radicals. However, it has been reported that the aryloxyl radicals are sometimes unstable showing a prooxidant behavior (Bors et al. 1995).

The metal chelating activity of flavonoids results either (i) in the formation of metal-chelates which have lower catalytic efficiency in Fenton reaction than pure flavonoids (decreased number of metal binding sites is usually associated with lower catalytic activity) or (ii) metal flavonoid chelates can intercalate into DNA of cancer cells, triggering cell death (Simunkova et al. 2021).

Although resveratrol is a non-flavonoid polyphenol and stilbenoid and has various health-beneficial effects. Resveratrol has been associated with the so-called “French paradox”, which has been reported in 1992, based on epidemiological data from the French population known for the low incidence of cardiovascular diseases despite the consumption of a high-fat diet (Catalgol et al. 2012). A low incidence of cardiovascular diseases has been linked to the consumption of red wine containing a high concentration of resveratrol, a multipurpose molecule possessing various beneficial and health-protecting properties, involving antioxidant, and antitumor properties. In addition, resveratrol interferes with nuclear factor kappa B and apoptotic pathways.

#### Ergothioneine

**Fig. 14:**
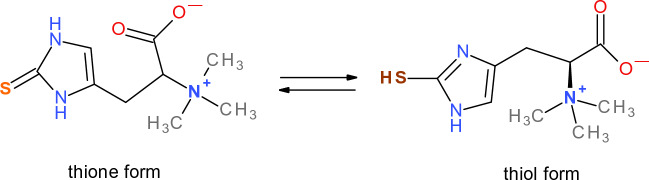
Structure of ergothioneine (ERG)

Ergothioneine (ERG) is a sulfur-histidine betaine amino acid possessing antioxidant properties (Fig. 14) (Borodina et al. 2020). ERG has been discovered early 20 century and is found in many fungi and actinobacteria. Since animals and plants are unable to synthesize ERG, the only route to acquire it, is from the diet or soil. ERG occurs in two tautomeric thione and thiol forms, the thione form being more abundant at physiological pH (Borodina et al. 2020). This molecule is unusually resistant against autooxidation by molecular oxygen, documented also by its thiol-disulfide couple standard redox potential, being − 0.06 V. ERG reacts fast with hydroxyl radicals, and its reaction with hydrogen peroxide and superoxide radical anion is somewhat slower (Fahey 2001).

ERG acts in erythrocytes, its abundance is dependent on diet, age, and health status including chronic diseases. Intake of ERG decreased biomarkers of oxidative stress such as 8-iso-PGF2α a sensitive marker of lipid peroxidation. ERG is a very good cytoprotecting agent against various cellular insults (Borodina et al. 2020).

Inflammation is an important component of oxidative stress, however, treatment based on the application of antioxidants, e.g. ascorbate, is not very effective, probably due to its possible prooxidant behavior under certain conditions (e.g. presence of free redox metals, Cu, Fe). ERG contains several functional groups (–COO^−^, –SH), through which can ERG chelate metal ions.

ERG has been found to be an effective antioxidant in cardiovascular diseases (Servillo et al. 2017), chronic pulmonary diseases (Rahman 2012), lung inflammatory diseases (Repine and Elkins 2012), and other pathologies. Intake of ERG has been linked with longevity (Beelman et al. 2019).

A positive correlation between ERG and its antidepressant properties has been reported (Nakamichi et al. 2016) Food-derived hydrophilic antioxidant ergothioneine is distributed to the brain and exerts antidepressant (Nakamichi et al. 2016) and neuroprotective (Yang et al. 2012) activities in mice. ERG ameliorates oxidative stress at the sites of injuries and has been classified as an “adaptive antioxidant” because it does not interfere with the physiological function of ROS (Halliwell et al. 2016).

#### Selenium

Selenium (Se) is a trace mineral found naturally in various foods and nutritional supplements. Se is an essential component of many proteins, called selenoproteins (Zhang et al. 2020). Selenoproteins are involved in the proper functioning of reproduction systems and the thyroid gland. In addition, Se protects cells against ROS-induced damage and infections. Selenoproteins are known for their antioxidant function, in particular against peroxides. Cochrane Reviews reported a 30% reduced risk of cancer within the group with a high intake of Se (Vinceti et al. 2018). Selenoproteins protect cell membranes from ROS-induced damage and keep blood platelets in a healthy state, both factors are responsible for the healthy functioning of the cardiovascular system. Observational studies revealed that both high and low intake of Se is associated with an increased risk of cardiovascular disease (Rayman 2012).

Since the thyroid gland contains a high amount of selenium, it has been expected that supplementation by Se may help to improve thyroid function. However, the results were not straightforward (Winther et al. 2015). The thyroid gland contains iodine, this element and Se have a synergistic relationship for a healthy thyroid gland and therefore both these elements in proper amounts are needed. Supplements containing Se help to boost the immune system, Se is sometimes combined with vitamins C and E (Shakoor et al. 2021).

#### Melatonin

**Fig. 15:**
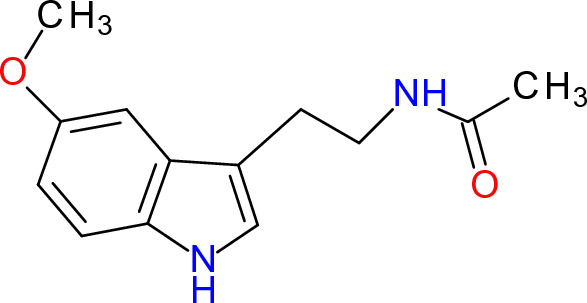
Structure of melatonin

Melatonin (Fig. 15) is a hormone produced by the pineal gland in response to dark. Melatonin is termed the “hormone of darkness”. Melatonin acts together with vitamin D as “light sensors” to meet the requirements for light and dark (Minich et al. 2022). The pineal gland produces approximately 0.5 mg of melatonin per day, depending on age, lifestyle, physical exercise, and use of medications, and its production declines with age. Melatonin is found in various tissues other than the pineal gland, involving the pancreas, liver, brain, lens, thyroid, and others (Mahmood 2019). Practically all body fluids, including breast milk, contain melatonin.

Recently obtained results indicate that mitochondria play an important role in the production, metabolism, and activity of melatonin (Tan et al. 2016; Tan and Reiter 2019). It has been shown that mitochondria stimulate melatonin production by cellular needs rather than by the circadian (light/dark) cycle. Melatonin occurs in mitochondria in higher abundance compared to blood. This is due to the higher requirements for an antioxidant pool in mitochondria containing electron transport chain (Tan et al. 2015).

Many studies, both in vitro and in vivo reported melatonin as a very good scavenger of free radicals, especially in cancers, neurodegenerative disorders, and psychiatric diagnosis. Melatonin is very efficient also in inflammatory diseases. A molecule of melatonin has a high capacity to scavenge several oxygen or nitrogen-derived radicals at one time and interact with both, receptor-dependent and -independent processes. Melatonin interferes with inflammatory processes by interacting with cyclooxygenase (COX2), which is a key mediator of inflammatory processes and promotes apoptosis in malignant cells (Favero et al. 2017; Chitimus et al. 2020).

Melatonin can activate antioxidant enzymes such as SOD, Catalase, and Glutathione peroxidase to boost the immune system or suppress the activity of pro-oxidant enzymes, such as xanthine oxidase. Since the production of melatonin with age is gradually decreasing, such an event may to some extent correlate with the development of many chronic diseases (Hardeland 2012). Thus, long-term supplementation of an aging population with adequate doses of melatonin may help to improve the health status of the targeted group of elderly people. Interestingly, in 2020–2021, during the COVID-19 pandemic period, melatonin was the most sought-after supplement to optimize levels of cytokines linked with disturbed sleeping (Minich et al. 2022). Melatonin and vitamin D act synergistically in skin protection (Wacker and Holick 2013).

## Measurement of antioxidant enzymes: superoxide dismutase, catalase, and glutathione peroxidase levels

The protocols to evaluate the levels of antioxidant enzymes are of key importance to know whether alterations in antioxidant enzymes are directly linked with the disease’s incidence and development or just represent secondary symptoms occurring parallel with the diseases, or even consequence of the diseases.

### Superoxide dismutase activity assay

**Fig. 16:**
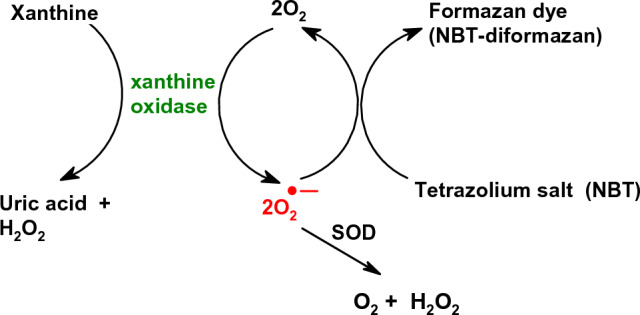
SOD Assay. Xanthine oxidase converts xanthine and O_2_ to uric acid and H_2_O_2_ and generates O_2_^·−^ which reduces a tetrazolium salt (NBT) to a colored Formazan (NBT-diformazan)

SOD activity is evaluated using the xanthine-xanthine oxidase (XO) used to generate superoxide radical anion. As an indicator of superoxide formation is used nitroblue tetrazolium (NBT). SOD competes with NBT for superoxide radicals and the extent of NBT reduction can be monitored by measuring absorbance at 550 nm. Inhibition of NBT is proportional to the amount of SOD (Fig. 16). Catalase is used to remove hydrogen peroxide formed in the course of the conversion of superoxide by SOD. The activity of enzymes is expressed in units per mg of protein or per cell or µg of DNA (Lowry et al. 1951; Burton 1956).

Evaluation of the total amount of SOD gives an overview of the overall SOD detoxifying capacity of the studied system. However, the determination of Mn-SOD and Cu, Zn-SOD will provide more information, since the location and response of different SODs differ. Cu, Zn-SOD is known to be increased as a response to certain therapies or in some diseases therefore determination of activities of different SODs is necessary (Crapo et al. 1992).

### Catalase assay

**Fig. 17:**
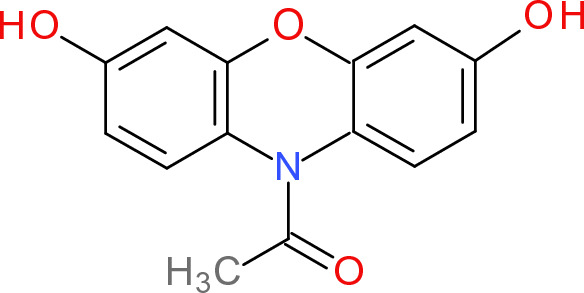
Structure of OxiRed™ (1-(3,7-Dihydroxy-10H-phenoxazin-10-yl)ethanone)

In the majority of cells, catalase decomposes a mild redox signaling hydrogen peroxide molecule to oxygen, preventing thus damage by the Fenton reaction. Catalase activity measurements can be performed on various biological samples, including cells, as well as biological tissues. Catalase reacts with hydrogen peroxide to form oxygen and water. Catalase assay is based on the reaction of unconverted hydrogen peroxide with the probe—1-(3,7-Dihydroxyphenoxazine-10-yl)ethenone (OxiRed™) (Fig. 17) to form a product detectable colorimetrically at 570 nm using a spectrophotometer or fluorometrically at 535/587 nm using a fluorescence spectrometer. Thus, the catalase activity in the studied system is conversely proportional to the obtained signal (Hadwan 2018).

Too high levels of hydrogen peroxide may lead to saturation of catalase active sites resulting in the inhibition of the enzyme. Catalase activity is defined in international units equal to the amount of enzyme necessary to decompose 1 µM of hydrogen peroxide per minute at pH 7.0 and 25 °C at a substrate (hydrogen peroxide) concentration of 65 mM.

### Glutathione peroxidase assay

**Fig. 18:**
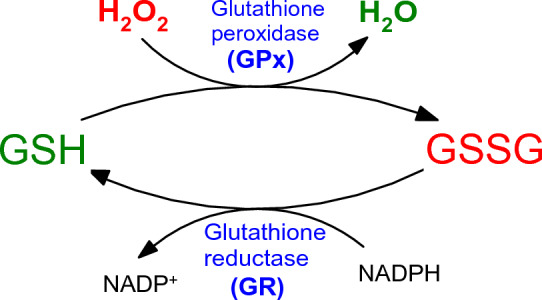
Action of glutathione peroxidase (GPx) resulting in the formation of oxidized glutathione (GSSG) and regeneration of reduced glutathione (GSH) from oxidized glutathione (GSSG) by glutathione reductase (GR). This reaction uses NADPH as the reducing cofactor and maintains a constant level of GSH

In the measurement of glutathione peroxidase (GPx) activity, GPx oxidizes reduced glutathione (GSH) to produce oxidized glutathione (GSSG) and simultaneously reduces cumene hydroperoxide (or tert-butyl-hydroperoxide) to water (Takebe et al. 2002). Glutathione reductase (GR) in the next reaction step reduces the GSSG back to its reduced form (GSH). This reaction uses NADPH as the reducing factor (Fig. 18). Decrease of NADPH (measured at the wavelength of 340 nm) is proportional to GPx activity.

One unit of GPx will cause the formation of 1 µM of NADP^+^ from NADPH at 25 °C at pH 8.0 per minute by the Glutathione reductase.

## Oxidative stress, oxidative eustress, and oxidative distress

The term oxidative stress in relation to biological systems appeared for the first time in the scientific literature in the early 70’s (Azzi 2022). The concept of oxidative stress in greater detail has been for the first time defined by Prof. Sies in 1985 (Sies 1985, 2018, 2020a). Oxidative stress was defined as “an imbalance between oxidants and antioxidants in favor of the oxidants, leading to a disruption of redox signaling and control and/or molecular damage” (Sies 2020b). The term oxidative stress can be extended to terms oxidative eustress and oxidative distress. Oxidative eustress means “good stress” and is characterized by low to mild levels of oxidants involved in the regulation of various biochemical transformations such as carboxylation, hydroxylation, peroxidation, or modulation of signal transduction pathways (Forman et al. 2010). Oxidative distress (“negative stress”) is a term used for the description of a condition responsible for damage to biological molecules, involving proteins, membranes, and DNA. However, the double-edged sword of oxidative distress may involve a desirable mechanism of apoptosis, especially in cancer cells, either by natural causes or by anticancer drugs (Simunkova et al. 2019a, b). Higher levels of oxidative stress (distress) are required in phagocytosis and thyroid hormone synthesis. Suppressed oxidative distress in those cases may have a negative impact on health.

Free radicals, or ROS, or oxidants are species occurring in all organelles or tissues, where they may act. With the advent of the oxidative stress concept, various disease states of an organism have been linked with the damaging action of ROS. It has been proposed that oxidative stress-related diseases can be “cured” by the enhanced supply of antioxidants. However, over the years, large-scale clinical trials proved only limited health benefits of taking antioxidants (Halliwell 2022). We can mention one example for all, the ATBC (α-Tocopherol, β-Carotene) trial described above.

Since the enhanced antioxidant status has not been shown to be effective in the alleviation of oxidative stress, a new approach based on the thiol redox pool has been proposed (Jones 2006). In view of this, a more complex definition of oxidative stress, based on the disruption of redox signaling has been proposed (Jones 2006). Redox signaling is substantiated by the major cellular GSH/GSSG ratio and thioredoxin (–SH_2_/–SS–) and Cys/Cys-SS ratios.

There are many assays, which can be applied in the “quantification” of oxidative stress, however, whether oxidative stress is a consequence or cause of the diseases is impossible to conclude. The quantification methods include antioxidant assays (e.g., Glutathione assay, Ascorbic acid assay, Antioxidant enzyme activity assay), DNA damage/repair (e.g., 8-OH Guanine, double-strand break assay), peroxidation of lipids (e.g., 4-hydroxynonenal assay, malondialdehyde assay), ROS assay (e.g., H_2_O_2_ assay, NO^·^ assay), protein modification (e.g., Advanced glycation end products, protein carbonyl assay), and Oxidase/Peroxidase activity (e.g., Monoamine oxidase assay, peroxidase/H_2_O_2_ assay).

All these assays have a common denominator based on the measurement of the extent of the damage caused by the action of oxidants. The concept of “total antioxidant capacity” has serious limitations and cannot be simply transferred to in vivo conditions (Sies 2007).

## Can oxidative stress be suppressed by the action of low molecular weight antioxidants?

Many antioxidants exhibit impressive antioxidant/radical scavenging activity in various in vitro tests. However, in many cases, the results from in vitro tests cannot be simply translated to in vivo conditions. There are many examples of efficient antioxidants under in vitro conditions that undergo a switch from antioxidant to prooxidant behavior, or partly or completely lose their antioxidant properties under in vivo conditions (Simunkova et al. 2021).

Many natural antioxidants taken from diet or from supplements have very limited bioavailability and their in vitro antioxidant activity and ability to regenerate themselves have not been confirmed in cells or tissues (Jomova et al. 2019). Typical examples are flavonoids which are due to the number of hydroxyl groups very good in vitro antioxidants, however, under in vivo environment they undergo structural changes and transformations and therefore their antioxidant capacity is significantly reduced. Various disease states are characterized by disturbed metal homeostasis, such as hemochromatosis (iron overload) and flavonoids in the presence of iron (or other redox metals) behave as prooxidants, increasing the total amount of ROS (Valko et al. 2005).

Another example with different in vitro and in vivo performances involves melatonin, a hormone secreted by the pineal gland that plays an important role in sleep–wake regulation. Many in vitro experiments under different experimental conditions confirmed the good antioxidant activity of melatonin, however, levels of melatonin in certain body organs might not be high enough to exhibit antioxidant properties (Sanchez-Barcelo et al. 2010).

Teas are associated with health-beneficial, and antioxidant properties mainly due to the presence of catechins. A tea polyphenol, Epigallocatechin 3-gallate (EGCG) is a highly abundant catechin in green teas. While EGCG is a good antioxidant in various radical scavenging assays, in plasma is its antioxidant activity limited (Rasaei et al. 2021).

Figure 9 documents that due to the favorable values of standard one-electron reduction potential of vitamins E and C, is vitamin C able to regenerate Vitamin E (α-Tocopherol, α-TOH) from its α-Tocopheryl radical (α-TO^·^) form. Vitamin E protects membrane lipids against peroxyl radicals. Since peroxidation of lipids is a prerequisite of atherosclerotic plaque formation, supplementation of risky patients with vitamin E and vitamin C may prevent the incidence of atherosclerosis. In a 3 years randomized trial of the effect of vitamins E and C in atherogenic prevention has been shown that the subjects with a predisposition to atherosclerosis supplemented with vitamin E have increased plasma concentration of this vitamin (Salonen et al. 2000). Contrary to expectations, additional supplementation with vitamin C did not increase the level of vitamin E, which documents, that the in vitro redox recycling of vitamin E by vitamin C does not work in humans. Further studies documented the oxidation of vitamin E after the attack of ROS, however, the oxidation of vitamin E has not provided protection against lipids or damage to other biomolecules associated with chronic diseases (Niki and Noguchi 2021).

The presented results indicate, that in man, supplementation with high doses of antioxidants does not always ameliorate the oxidative stress component of various disease states of an organism or oxidative stress is not the only cause of the diseases. In the next sections, we will discuss several chronic diseases and aging characterized by oxidative stress as a common denominator. Those cases, in which antioxidant therapy has been shown to have a positive effect, will also be discussed.

## Oxidative stress biomarkers

As discussed above, oxidative stress is characterized by an imbalance between oxidants and antioxidants in favor of oxidants and may result in irreversible chemical modifications. The process of validation of oxidative stress biomarkers has received in the past decade significant attention (Dalle-Donne et al. 2006). Of great importance are oxidative stress biomarkers in clinical practice where they may be helpful in establishing a diagnosis and proposing a treatment procedure (Frijhoff et al. 2015). The WHO defined a biomarker as “any substance, structure, or process that can be measured in the body or its products and influence or predict the incidence of outcome or disease” (WHO 2001). A reliable biomarker of oxidative stress must fulfill the following criteria: (a) clinical relevance (b) specificity for certain diseases (c) reflect the stage of the disease (d) quantification of the biomarkers should show a high degree of reproducibility.

Markers related to oxidative stress can be divided into several categories: (a) Markers of ROS formation, (b) Markers of Antioxidant protection, (c) Biomarkers of ROS-induced modifications, (d) Downstream functional markers of damage caused by ROS.

### Markers of ROS formation

#### Xanthine oxidase

Some of the ROS-producing enzymes such as xanthine oxidase (XO), Myeloperoxidase, NADPH oxidase, eNOS, etc. may generate ROS not only in intracellular space but under certain conditions also in circulation, especially when their relevant substrates are present.

**Fig. 19:**
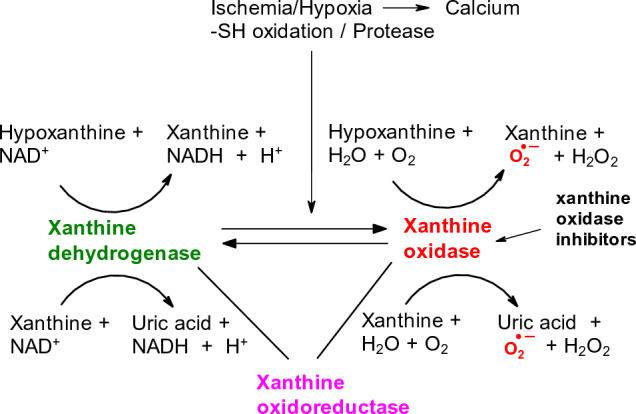
Xanthine dehydrogenase (XDH) can be transferred to xanthine oxidase (XO) either irreversibly, by partial proteolysis, or reversibly, during the oxidation of thiols. XO transfers hypoxanthine to xanthine and then to uric acid, as the substrate is used oxygen. XDH maintains the same transformation, however, with NAD^+^ as the substrate. XDH and XO are collectively referred to as Xanthine oxidoreductase (XOR)

Xanthine oxidase catalyzes the oxidation of xanthine to uric acid (antioxidant) accompanied by the formation of superoxide radical anion, O_2_^·−^, and hydrogen peroxide (McCord 1985). Superoxide radical anion, generated from the enzyme xanthine oxidase (XO) plays an important role in the pathogenesis of ischemia-induced tissue injury (Vickenson and George 2020). XO inhibitors primarily suppress the formation of uric acid (not only superoxide radical) that has not only antioxidant but also proinflammatory properties. Xanthine dehydrogenase (XDH) and Xanthine oxidase are two forms of the enzyme xanthine oxidoreductase (XOR). XO transfers hypoxanthine to xanthine or xanthine to uric acid accompanied by the formation of superoxide radicals. XO uses oxygen molecules as the source of electrons. Thus, XO is one of the most important ROS-generating enzymes and can be used as a sensitive biomarker of oxidative stress. Similarly to XO, XDH also transfers hypoxanthine to xanthine and xanthine to uric acid, however, XDH uses NAD^+^ as the substrate. XDH can be converted to XO by the oxidation of thiols (Fig. 19) (Enroth et al. 2000).

#### Myeloperoxidase

**Fig. 20:**
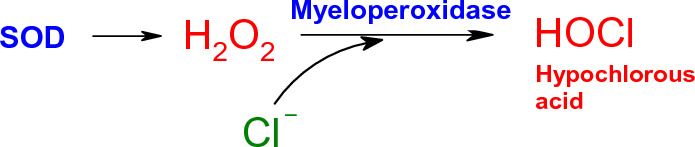
Myeloperoxidase catalyzes the reaction between hydrogen peroxide and chlorine ions to form hypochlorous acid

Myeloperoxidase (MPO) is a heme-containing peroxidase released mainly by neutrophils (Davies and Hawkins 2020). MPO converts hydrogen peroxide and chloride anions to hypochlorous acid and protects the organism against toxins and pathogens. Thus, the main role of MPO is to maintain the immune system and antimicrobial protection against various toxins and pathogens. MPO plays a role also in inflammatory diseases and has been suggested as a potential biomarker of inflammation. The only problem is a rather poor reproducibility in the quantitative evaluation of MPO as a biomarker across laboratories. MPO-derived oxidant 3-Cl-Tyr is occurring in high-density lipoprotein (HDL) and therefore is a good biomarker of cardiovascular diseases (Bergt et al. 2004) (Fig. 20).

### Biomarkers of ROS-induced chemical modifications

This class involves biomarkers resulting from the direct attack of ROS/RNS in biomolecules such as modifications of proteins.

#### Protein carbonyls

Protein side chains are irreversibly modified by direct attack of ROS. Protein carbonyls, [C=O], (aldehydes or ketones) are protein modifications, formed on side chains of proteins following the oxidation of proline, arginine, lysine, and threonine. These products are structurally stable and suitable for chemical detection (Stadtman 2004). An alternative pathway of protein carbonyl formation is through oxidative cleavage of proteins. Carbonyl groups can be incorporated into proteins by secondary reactions between cysteine or histidine with aldehydes (involving for example 4-hydroxy-2-nonenal or malondialdehyde) formed during the peroxidation of lipids. Protein carbonyls have been found to be elevated in Alzheimer’s diseases, diabetes, renal diseases, and other disorders (Gil et al. 2006). In addition, HIV and hepatocellular carcinoma patients have increased levels of protein carbonyls.

#### Advanced glycation end products (AGEs)

Heterogenous molecules originating from the nonenzymatic reaction of glucose or other saccharides with proteins or lipids are termed advanced glycation end products (AGEs) (Perrone et al. 2020). AGEs are induced by various factors such as a hypercaloric diet, smoking, and high-temperature cooked foods. AGEs are known to induce via oxidative stress several stress-induced transcription factors with the concomitant production of inflammatory cytokines. About 20 various AGEs have been detected in blood and tissues, as well as in foods. The most abundant non-fluorescence AGEs involve carboxymethyl-lysine (CML), and carboxyethyl-lysine (CEL). Fluorescence AGEs involve methylglyoxal-lysine and pentosidine (Singh et al. 2001). All these molecules share lysine residue in their structures. Accumulation of AGEs causes hyperglycemia and hyperlipidemia, inflammation, and induces oxidative stress.

#### Oxidized low-density lipoprotein (OxLDL)

Oxidized low-density lipoprotein is linked with the occurrence of atherosclerosis (Lara-Guzman et al. 2018). OxLDL is formed by various modifications of lipids and apolipoprotein B by the process of peroxidation of lipids. Atherosclerosis is further promoted via immunology and inflammatory mechanisms. OxLDL is evaluated in blood plasma by immunological methods using various antibodies (Liu and Frostegard 2018). However, the problem is the poor reproducibility of quantitative methods, and therefore the application of oxLDL as a marker of oxidative stress in the prediction of cardiovascular diseases is doubtful.

#### Products of lipid peroxidation—4-Hydroxynonenal, malondialdehyde

**Fig. 21:**
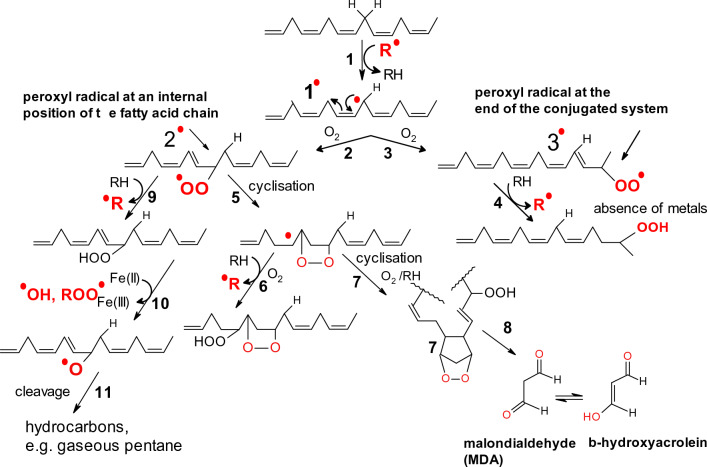
Pathways of lipid peroxidation: polyunsaturated fatty acids are susceptible to oxidation (reaction 1) resulting in the formation of carbon-centered radicals 1^·^. Carbon-centered radicals react with molecular oxygen to form peroxyl radicals at an internal position of fatty acid chain 2^·^ (reaction 2) or at the terminal group 3^·^ (reaction 3). Radical 2^·^ can react by cyclization (reaction 5) to form cyclic peroxide which is then by another cyclization reaction (reaction 7) via an intermediate product (7) transformed (reaction 8) to malondialdehyde (MDA). Peroxyl radicals in the internal position (2^·^) can alternatively react by hydrogen abstraction (reaction 9) to form lipid hydroperoxides (-OOH). Hydroperoxides can further react with the redox metals [e.g. Fe(II)] to form alkoxyl radicals (RO·) which, following cleavage (reaction 11) may form pentane in the gaseous state, another marker of lipid peroxidation process. Peroxyl radicals located at the end of the conjugated system (3^·^) are reduced in the absence of redox metals to stable hydroperoxides (-OOH) (reaction 4)

Polyunsaturated fatty acids (PUFAs) represent the main target of the lipid peroxidation process. Lipid hydroperoxides react fast with Fe^2+^ to form alkoxyl radicals (LO^·^), and reaction with Fe^3+^ results in the formation of lipid peroxyl radicals (LOO^·^), however, the latter reaction is slower (Valko et al. 2006). Both peroxyl radicals and alkoxyl radicals are further cyclized and dependent on the type of polyunsaturated fatty acid, degraded into reactive aldehydes such as 4-hydroxynonenal (4-HNE) or malondialdehyde (MDA) (Fig. 21) (Ramana et al. 2014). The direct assessment of MDA and 4-HNE is not very sensitive, since the major portion of these reactive aldehydes is attached to proteins or other biomolecules and therefore before analysis, should be released, otherwise, they remain undetected (Esterbauer et al. 1991).

#### F_2_-isoprostanes

Isoprostanes are specific prostaglandin-like molecules formed by the non-enzymatic ROS-mediated peroxidation of PUFAs, involving for example arachidonic acid (Milne et al. 2015). The oxidation of arachidonic acid proceeds by various reactions resulting in the formation of various products. The overall biosynthetic pathway of isoprostanes involves the formation of arachidonyl radicals and peroxyl radical isomers with endocyclization, forming bi-cycloendoperoxide regiomers reduced to F2-isoprostanes (F2-IsoPs). Increased amounts of F2-IsoPs are detected in cardiovascular diseases and other clinical conditions (Milne et al. 2015).

#### 3-Nitrotyrosine (Tyr-NO_2_)

Tyr-NO_2_ is a stable marker of oxidative and nitrosative stress in inflammatory disorders. The formation of Tyr-NO_2_ can be described by the following reactions (Souza et al. 2008)

26 $${\text{ONOO}}^{ - } { } + {\text{ CO}}_{2} { } \to { }^{ \cdot } {\text{NO}}_{2} { } + {\text{ CO}}_{3}^{ \cdot - }$$

27 $${\text{CO}}_{3}^{ \cdot - } { } + {\text{ Tyr }} \to {\text{ Tyr}}^{ \cdot } { } + {\text{ HCO}}_{3}^{ - }$$

28 $${\text{Tyr}}^{ \cdot } + \;^{ \cdot } {\text{NO}}_{2} \to 3{\text{NO}}_{2} {\text{Tyr}}$$

Despite the fact, that Tyr-NO_2_ is a stable marker of oxidative/nitrative stress in inflammatory diseases, further studies are necessary to carry out before it can be routinely used. Plasma levels of Tyr-NO_2_ sensitively reflect the health status of an organism following pharmacotherapy. Future research will explore if Tyr-NO_2_ may be an alternative marker for C-reactive protein (CRP) a well-established marker of acute inflammation, synthesized by upregulated inflammatory cytokines in the liver (Baumgartner et al. 2018).

#### Glutathione

Glutathione (GSH) is a tripeptide occurring in high (mM) concentration in the intracellular environment. Oxidized glutathione (GSSG) can be reduced back to GSH by Glutathione reductase (GR) or NADPH. Increased ROS formation depletes GSH levels or decreases GSH/GSSG ratio. In the cells, the GSH/GSS ratio is greater than 30, and in the serum is about 10 times lower (Hwang et al. 1992). Most studies evaluated the concentration of GSH in erythrocytes, where GSH concentration is high, however, whether it is a good measure of oxidative stress, is still under question (Francioso et al. 2021). Lower GSH levels do not necessarily have to be related to increased oxidative stress, but rather to suppressed cysteine pools as a result of a specific diet.

Various pathological states are characterized by decreased levels of GSH, an example being fluorescence detection of GSH within the cells in HIV patients. Evaluation of GSH is important also among patients with high levels of GSH derivatives or precursors (Atkuri et al. 2007).

#### Protein thiols and mixed disulfides

Protein glutathionylation has been discussed in Sect. 5.2.3. The level of glutathionylated proteins reflects increased oxidative stress (Valko et al. 2006). Glutathionylated proteins occur mainly in intracellular space because GSH is located in the cytoplasm and Cys residues of proteins are in a reduced form and able to form mixed disulfides. On the contrary, proteins in the extracellular environment occur in the form of disulfide bridges. Transthyretin is a transporter of thyroxine in cerebrospinal fluid and plasma and is identified as a glutathionylated protein (Liz et al. 2020). It has been proposed that glutathionylated hemoglobin detected in erythrocytes by Mass spectrometry (MS) is a good marker of oxidative stress under various conditions, such as renal diseases, diabetes, or hyperlipidemia (Ghezzi 2013). Elevated levels of cysteinylated albumin detected by MS have been associated with kidney diseases and diabetes, and in addition, hepatic diseases (Nagumo et al. 2014).

#### Oxidation of DNA and RNA

**Fig. 22:**
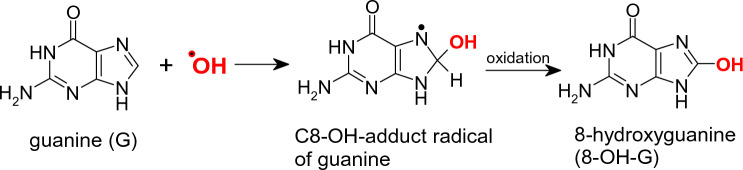
Reaction of hydroxyl radical with guanine

One of the main targets of oxidative attack is DNA (Cooke et al. 2003). To date, more than 100 DNA adducts with ROS have been detected and the methods of their quantification have been well established (Dizdaroglu et al. 2002). The oxidized nucleosides are detected in the urine and their content has been related to the total oxidative stress of an organism. The urinary markers are relevant to oxidative stress conditions of all organisms. One of the best-studied oxidative DNA markers is 8-OH-Gua (Fig. 22). The most sensitive method for the detection of oxidative DNA damage is the combination of GC–MS and HPLC electrochemical detection.

Severe oxidation of DNA is a precursor for the risk of some cancers, in particular, breast and lung cancers (Loft et al. 2013). Increased RNA oxidative damage is a prediction for diabetes complications and increased risk of breast cancer among patients suffering from diabetes 2.

#### Thioredoxin and peroxiredoxin

Oxidative stress may be caused by an impaired antioxidant defense system. There are several antioxidant enzymes well reflecting oxidative stress. Thioredoxin (TRX) is a small (12 kDa) multifunctional antioxidant redox protein containing two Cys residues possessing antioxidant function. Thioredoxin protects thiols in a reduced state against oxidation. It has been proposed that the secretion of TRX is a response to increased oxidative stress which can be inhibited by the antioxidants (Kondo et al. 2004). TRX has been found to be elevated in patients suffering from cancer and AIDS. It also has been reported that several other diseases correlate with plasma or serum levels of TRX (Qi et al. 2015).

Another antioxidant enzyme whose level is a good biomarker of oxidative stress is Peroxiredoxin (PRX). PRX is a family of enzymes that detoxify peroxides and peroxynitrites with high turnover. Oxidized PRX6 in cerebrospinal fluid has been found to correlate with oxidative stress following brain injury. PRX4 has been proposed as a suitable biomarker of oxidative stress in diabetes (Abbasi et al. 2014). PRX2 has been found to be secreted by inflammatory cells and is elevated in synovial fluids and serum in patients with rheumatoid arthritis (Szabo-Taylor et al. 2012).

Transcription factor Nrf2 is activated in response to ROS-induced oxidative stress and triggers the expression of its target genes through binding to antioxidant response elements (Ma 2013). In fact, Nrf2 regulates the expression of many antioxidant protection genes in the liver. Some of these genes encode the synthesis of enzymes participating in the synthesis of GSH and Trx. Nrf2 is a good biomarker of various cancers. Nrf2 has been found in high abundance in biopsies of rapidly dividing cancer cells with bad responses to chemotherapy. Most valuable results have been obtained for adenocarcinoma (Inoue et al. 2012), breast (Onodera et al. 2014), and lung (Yang et al. 2011) cancers. An overview of oxidative stress biomarkers is outlined in Table 2 (Frijhoff et al. 2015).

**Table 2:** Oxidative stress biomarkers and their relevance to human diseases

Oxidative stress biomarker	Disease	References
Malondialdehyde (MDA)	Alzheimer’s disease, atherosclerosis, cancer, amyotrophic lateral sclerosis, obesity	Esterbauer et al. (1991)
4-Hydroxynonenal (HNE)	Cardiovascular disease, atherosclerosis, Alzheimer’s disease, Parkinson’s disease, asthma,	Waeg et al. (1996)
F_2_-isoprostanes (F2-isoPs)	Cardiovascular disease, atherosclerosis, amyloidosis, obesity, multiple sclerosis, heart failure, asthma, diabetes, respiratory distress symptom, pancreatitis, rheumatoid disease, obesity, cystic fibrosis, Huntington’s disease	Davies and Robert (2011)
Advanced glycation end products (AGEs)	Atherosclerosis, Alzheimer’s disease, multiple sclerosis	Thome et al. (1996)
8-oxo-Guanine	cancer, amyloidosis	
Oxo-Low density lipoproteins (Oxo-LDL)	Cardiovascular disease, atherosclerosis, amyloidosis, obesity, Alzheimer’s disease, Parkinson’s disease,	Steinberg et al. (1989)
Acrolein	Cardiovascular disease, atherosclerosis, Alzheimer’s disease, ischemia/reperfusion,	Chen et al. (2014)
Cysteine/Cystine	Atherosclerosis, AIDS, cystic fibrosis, kidney failure, chronic obstructive pulmonary disease	Mills et al. (2000)
GSH/GSSG (reduced/oxidized glutathione)	Cardiovascular disease, Alzheimer’s disease, cancer, ischemia/reperfusion, obesity, rheumatoid arthritis, chronic obstructive pulmonary disease	Hwang et al. (1992)
NO_2_-Tyr	Cardiovascular disease, atherosclerosis, Alzheimer’s disease, cancer, multiple sclerosis, asthma, rheumatoid arthritis, obesity, cystic fibrosis	Herce-Pagliai et al. (1998)
3-Cl-Tyr	Cardiovascular diseases, atherosclerosis, heart failure, obesity, cystic fibrosis, kidney diseases, chronic obstructive pulmonary diseases	Kettle et al. (2014)

## Oxidative stress, diseases, aging, and antioxidant interventions

Oxidative stress has been related to many pathological states of an organism (Halliwell and Gutteridge 2015). The pathology of diseases, in connection with oxidative stress, can be caused either by the primary or secondary effect of oxidative stress, however, this classification is only provisional and does not cover all possible cases.

Cells subjected to various stresses, including oxidative stress are characterized by cell cycle arrest, termed cellular senescence, found in both in vivo and in vitro cells. Senescence is considered a key hallmark of aging. However, while senescence occurs throughout the lifespan, aging is a progressive decline in time (Zhang et al. 2022). One of the most critical aspects of senescence is the senescence-associated secretory phenotype (SASP). SASP are phenotypic changes such as the formation of bioactive secretome, triggered by a stress response—cellular senescence. Senescent cells can secrete soluble inflammatory cytokines, growth factors, interleukins, chemokines, degradative enzymes such as matrix metalloproteinase, and insoluble extracellular matrix components. ROS are known to interfere with various components of SASP.

Oxidative stress, cellular senescence, and SASP are involved in the incidence of chronic diseases involving cardiovascular diseases, cancer, metabolic, renal, lung, and neurological disorders (Alzheimer’s and Parkinson’s disease), and other diseases. With respect to antioxidant interventions, apart from vitamin E, other antioxidants such as carotenoids, ascorbic acid, and flavonoids have been shown to have a very mild effect on preventing oxidative damage (Halliwell 2020; Forman and Zhang 2021; Mangione et al. 2022). For each disease reported below, we discuss a given antioxidant that has shown promising results in the alleviation of oxidative damage. In this context, suitably selected natural, diet-derived antioxidants seem to be a viable way to decrease oxidative damage and prevent human disease and aging.

### Cardiovascular diseases

Cardiovascular diseases (CVD) have multifactorial origins and according to WHO are the major cause of death worldwide (Benjamin et al. 2017). One of the major causes of CVD is atherosclerosis and remodeling of blood vessels resulting in restrictions of blood flow. Cardiovascular diseases involve several other pathologies, the major being hypertension, vascular diseases, coronary artery diseases, heart failure, and other pathologies (Dubois-Deruy et al. 2020).

#### Cardiovascular diseases and oxidative stress

A central role in redox signaling of myocardial functioning is played by several kinases. One of the most important is H_2_O_2_-activated Ca/calmodulin-dependent kinase II (CAMKII) responsible for excitation–contraction coupling. In addition, JNKs (c-Jun N-terminal kinase) play an important role in insulin resistance, and p38 MAPK (p38 mitogen-activated protein kinase) has been shown to stimulate the uptake of glucose (Bennett and Lawan 2020). The activity of the protein kinase A (PKA) family depends on cellular levels of cyclic AMP (cAMP). PKA is a key regulator of cardiac functioning and morphology and is induced by various hormones, neurotransmitters, and stress signals. Increased activity of PKA promotes Ca^2+^ cycling and increases contraction of the myocardium.

Nuclear factor E2-related factor 2(Nrf2) is a transcription factor involved in oxidative stress regulation. Nrf2 translocates into the nucleus where it binds to the antioxidant response element (ARE), termed also as the electrophile response element. ARE is involved in the regulation of the expression of various antioxidant enzymes such as SOD, glutathione peroxidase, thioredoxin reductase, and other enzymes (Lismont et al. 2019).

Under normal conditions, NO^·^ has cytoprotecting properties and is an effective vasodilator. Nitric oxide at low levels supports ventricular function and oxygen consumption and at high concentrations induces inflammation and inhibits contractile function (Heusch et al. 2008). While exogenous nitric oxide triggers ischemic preconditioning, endogenous nitric oxide does not.

Increased oxidative stress causes modifications of all subcellular organelles, including oxidation of endoplasmic reticulum Ca^2+^-ATPase and contractile proteins (actin and myosin being the most important) resulting in contractile dysfunction (Steinberg 2013).

Various signs of increased oxidative stress have been reported in various cardiovascular-related diagnoses such as heart failure (Dai et al. 2012), ventricular hypertrophy (Dai et al. 2011), myocardial infarction (Merabet et al. 2012), diabetes (Anderson et al. 2009) and other conditions.

NADPH (NOX) are membrane-bound electron-transporting proteins responsible for ROS formation, primarily superoxide radical anion, O_2_^·−^, however, hydrogen peroxide can also be produced. Great progress in the past two decades resulted in the identification of the NOX family, consisting of NOX1, NOX2, NOX3, NOX4, NOX5, and dual oxidases DUOX1 and DUOX2 (Yoboue et al. 2018). In the cardiovascular system are expressed only NOX 1,2,4 and 5. NOX1,4 and 5 are expressed in smooth muscle cells and NOX2 and 4 are expressed in cardiomyocytes, fibroblasts, or endothelial cells. Vascular NOXs are regulated by a peptide-hormone, Angiotensin II (AngII) that stimulates the formation of NOX-dependent ROS which in turn activate Angiotensin II receptor type 1 (AT1) with a self-strengthening effect (Santillo et al. 2015). In addition, AngII regulates blood pressure and electrolytic homeostasis. NOX activity has been found to be increased in patients with metabolic syndrome and increased plasma levels of oxidized LDL and nitrotyrosine (Fortuno et al. 2006).

It has been reported that the myocardial infarction increases NOX2-derived ROS that activated apoptotic pathways such as Apoptosis signal-regulating kinase 1-p38 pathway ((ASK-1)-p38MAPK) and Ca^2+^/calmodulin-dependent protein kinase II (CAMKII). CAMKII is a serine/threonine-specific protein kinase regulated by Ca^2+^ (Erickson et al. 2008).

Endothelial dysfunction is characterized by suppressed production and utilization of nitric oxide associated with proinflammatory and prothrombotic properties. Endothelial dysfunction precedes the development of atherosclerosis and hypertension, the main risk factors for myocardial infarction and heart failure (Scioli et al. 2020).

ROS disrupts the vasoprotective nitric oxide signal transduction pathways resulting in the uncoupling of NO synthases, enzymes producing nitric oxide in the process of converting arginine into citrulline in the presence of NADPH and oxygen as necessary cofactors.

In total three, neuronal (nNOS or NOS1), inducible (iNOS or NOS2), and endothelial (eNOS or NOS3) isoforms of nitric oxide synthase (NOs) have been identified (Forstermann and Sessa 2012). All three forms occur in a variety of cells and tissues. NOS1 and NOS3 exhibit Ca-dependent activity and are expressed in striated muscle and endothelial cells. NOS2 isoform is expressed in the heart only under pathological conditions including inflammation. Uncoupling of NOs results in a switch from nitric oxide to superoxide radical and peroxynitrite and suppressed bioavailability of nitric oxide and vasoconstriction (Santillo et al. 2015). Peroxynitrites promote the progress of atherosclerosis and decrease the positive effect of nitric oxide on DNA and lipid oxidation, platelet aggregation, and proliferation of vascular smooth muscle cells.

Experiments using laboratory animals revealed that heart failure is also accompanied by increased superoxide radical formation by NOs in vessels leading to cardiomyopathy (Umar and Laarse 2010; Munzel et al. 2015).

A significant amount of ROS formed during mitochondrial dysfunction significantly contributes to the incidence of cardiovascular diseases (Bhatti et al. 2017). In a murine model of myocardial infarction, four weeks of coronary blockage resulted in mitochondrial ROS formation, mainly hydroxyl radicals (^·^OH), decrease in mitochondrial DNA. In addition, stimulated AngII produces ROS which in turn causes mitochondrial dysfunction, mitochondrial DNA lesions, heart failure, fibrosis, and cardiac hypertrophy (Dai et al. 2016). ROS produced by mitochondria oxidize proteins from complex I and II of the electron transport chain and negatively affect mitochondrial respiration.

Increased formation of ROS in mitochondria is a typical phenomenon in diabetic patients with cardiovascular diseases, typically with left ventricular hypertrophy and diastolic dysfunction (Jeong et al. 2016).

Mitochondrial dysfunction, oxidative stress, and cardiac hypertrophy have been observed in laboratory animals on a high-fat and sucrose diet. Increased oxidative stress induced by a high-fat diet has been observed at the expense of hydrogen peroxide formation. In patients with very high BMI has been observed increased apoptosis in right atrial cardiomyocytes (Nieman et al. 2011).

#### Therapeutic strategies against cardiovascular diseases

As it has been discussed above, oxidative stress is a serious condition in cardiovascular diseases. The most effective agents for ameliorating the onset and progression of cardiovascular diseases are antioxidants. For the last several decades the link between cardiovascular diseases and antioxidants has been investigated using cell cultures, animal models, and clinical trials (Dludla et al. 2022).

##### Vitamin E

As mentioned above, atherosclerosis is characterized by the thickening and hardening of the arteries and chronic inflammation during all phases of the disease (Libby et al. 2002). Vitamin E has been shown to have anti-inflammatory properties (Devaraj et al. 1996; Mandal et al. 2022) Monocytes are circulating leukocytes important in inflammation, tissue remodeling, and the immune system. Vitamin E has been reported to decrease monocyte recruitment as a result of suppressed formation of foam cells and reduce expression of scavenger receptors (SRs) on macrophages (Zapolska-Downar et al. 2000).

The non-antioxidant activity of vitamin E covers the increased bioavailability of nitric oxide, which slows the progression of atherosclerosis (Murohara et al. 2002). Vitamin E inhibits the protein kinase C pathway, NF-kappa B, and IkappaB degradation, all activated by oxidized low-density lipoprotein (LDL) (Goya et al. 2006). In addition, vitamin E is able to induce the expression of intercellular adhesion molecule (ICAM-1) by oxidized LDL in endothelial cells (Cominacini et al. 1997). C-reactive protein (CRP) is a protein synthesized in the liver. CRP is a good marker of inflammation and has been found to be reduced by vitamin E (Patrick et al. 2001).

Vitamin E has been primarily associated with the protection of biological membranes against lipid peroxidation since vitamin E is an efficient scavenger of peroxyl radicals and prevents this deteriorating process and consequently slows down atherosclerotic plaque formation (Reaven et al. 1993).

The two most important clinical trials on vitamin E are the Cambridge Heart Antioxidant Study (CHAOS) and Heart Outcomes Prevention Evaluation Study (HOPE). CHAOS trial demonstrated that α-tocopherol is able to reduce the incidence of myocardial infarction, however, failed to reduce the mortality in cardiovascular diseases (Stephens et al. 1996). Contrary to CHAOS, the HOPE trial failed (Yusuf et al. 2000) and scientists are unable to provide a rational explanation for this discrepancy. Therefore regular intake of vitamin E as a preventive agent against cardiovascular diseases is not recommended.

##### Vitamin C

Vitamin C can act effectively either alone or in combination with other vitamins. In addition to its antioxidant properties, vitamin C has some non-antioxidant properties. Vitamin C has been found to reduce the cholesterol level in patients with hypercholesterolemia (Rossig et al. 2001) and has been found to be effective against inflammatory conditions. Apoptosis mediated by inflammatory cytokines and oxidized low-density lipoproteins (LDL) can be attenuated by vitamin C (Rayment et al. 2003). Vitamin C can reduce apoptosis in vascular smooth muscle cells and prevent the instability of plaques (Lehr et al. 1994). Vitamin C has the ability to prevent LDL oxidation and consequently slows down the peroxidation of lipids (Maeda et al. 2000). Vitamin C has anti-inflammatory properties by preventing adhesion and aggregation of leukocytes to the endothelium (Padayatty et al. 2003).

Flow-mediated dilatation (FMD) is a term describing any vasodilatation (widening of blood vessels) of an artery following an increase in luminal blood flow (Thijssen et al. 2011).

Vitamin C has been found to have a beneficial effect on endothelial-derived nitric oxide-dependent flow-mediated dilation (Siow et al. 1999). Clinical trials evaluating the beneficial effect of vitamin C on cardiovascular disorders involve studies on endothelial dysfunction. Hyperglycemic patients experiencing vasodilation have a positive effect following intravenous administration (24 mg/min for 10 min) or oral dose (6 g during 2 days) (Levine et al. 1996).

Oral administration of 2 g of vitamin C in healthy populations helped to reduce aggregation platelet and arterial stiffness, however, the exact mechanism has not been fully explored (Wilkinson et al. 1999).

##### Polyphenols

Several epidemiological trials advocate the beneficial effect of polyphenols on the cardiovascular system (Condezo-Hoyos et al. 2021). Polyphenols contain hydroxyl groups capable of scavenging ROS and chelating redox-active metals resulting in the suppressed ROS (hydroxyl radicals) via Fenton chemistry or other mechanisms (Simunkova et al. 2019a, b).

Anthocyanins are water-soluble polyphenolic compounds responsible for the red–orange to blue-violet color of vegetables. Anthocyanins have profound anti-inflammatory, radical scavenging, and antioxidant properties (Castaneda-Ovando et al. 2009). Regular intake of anthocyanins (cca 200 mg per day) may decrease the risk of the development of cardiovascular diseases via suppressed oxidative stress and effective radical scavenging, reduced inflammation, and inhibition of endothelial dysfunction (Hertog et al. 1993). Delphinidin, an anthocyanin, a primary plant pigment, containing 6 hydroxyl groups in its structure possesses ROS scavenging properties and is able to prevent apoptosis of endothelial cells under in vitro conditions via interference with NO-signaling pathways (Martin et al. 2003). Delphinidin is also able to maintain homeostasis of various essential metals, predominantly calcium (Martin et al. 2003).

Flavonoids can prevent the formation of damaging radicals, such as hydroxyl radicals formed via the Fenton reaction by the chelation of redox-active metals (copper, iron, manganese) (Simunkova et al. 2021). Anthocyanins can improve the activity of antioxidant enzymes such as SOD, catalase (Chiang et al. 2006) and glutathione peroxidase (GPx), and glutathione in rats injected with isoproterenol (Karthikeyan et al. 2009).

Increased consumption of blueberries containing high content of anthocyanins improved endothelial functions and optimized blood pressure in rats fed with a high-fat diet (Rodriguez-Mateos et al. 2013). It has been reported that individuals supplemented with freeze-dried blueberries after consumption of a high-fat meal contained 19 of 25 anthocyanins present in the blueberries in human blood serum. The total amount of anthocyanins in the serum was directly proportional to increased oxygen radical absorbance capacity (ORAC) (Mazza et al. 2002). These results indicate that anthocyanins can suppress superoxide formation by NADPH oxidase (Wallace 2011).

Intake of strawberries for 30 days can decrease levels of LDL cholesterol and triglycerides and provide partial protection against cardiovascular diseases (Alvarez-Suarez et al. 2014). Another clinical trial involved 66 obese patients with metabolic syndrome who have included in their diet blueberries for two months. Analysis of serum revealed reduced levels of malondialdehyde (MDA), oxidized LDL, and optimized blood pressure indicating the beneficial effect of anthocyanins on cardiovascular diseases (Basu et al. 2010).

Catechins are a major group of flavanols contained in green tea. Various types of studies from epidemiological to in vitro postulated a tight relationship between consumption of green tea and cardiovascular health. Catechins have the ability to scavenge ROS directly or indirectly via chelation of redox-active metal ions, inactivate enzymes exhibiting prooxidant activity (iNOS), increase the activity of antioxidant enzymes (GPX, catalase, SOD), and inactivate transcription factors involving NF-κB (Babu and Liu 2008).

In a clinical trial, it has been reported that subjects drinking three cups of green tea for a period of four months have restored endothelial dysfunction and reduced 8-iso-prostoglandin-F2α, a useful oxidative stress marker. Long-term intake of catechins has been found to increase the bioavailability of Nitric oxide and decrease malondialdehyde (MDA) (Oyama et al. 2010).

##### Resveratrol

Resveratrol (3,5,4′-trihydroxy-trans-stilbene) is a phenolic compound belonging to the Stilbenoid group of naturally occurring compounds. Resveratrol contains three separated hydroxyl groups, thus its main antioxidant activity is related to ROS scavenging ability. Resveratrol has also been found to activate eNOS which may prevent hypertension in obese mice (Huang et al. 2018). Activation of eNOS by resveratrol may prevent hypertension and suppress phenylephrine-induced protein synthesis responsible for hypertrophy in cardiac myocytes in rats (Chan et al. 2008). Administrated resveratrol positively affects the synthesis of sirtuins, SIRT1 (a family of proteins NAD + -dependent histone deacetylases playing an important role in the regulation of metabolic pathways, see below) activating then superoxide dismutase 2 (Mn-SOD) expression in cardiomyocytes and improved cardiac functions (Tanno et al. 2010). Mice supplemented with resveratrol exhibit prolonged survival, less frequently occurring cardiac fibrosis, and improved diastolic function (Sung et al. 2015).

##### Vitamins B, D, and folic acid

Hyperhomocysteinemia is characterized by enhanced levels of non-proteinogenic α-amino acid homocysteine, an intermediate in methionine metabolism. This condition occurs approximately in about 5% of the population, however, among patients with cardiovascular diseases this condition occurs in up to 45% of patients and represents a serious risk of heart attack, endothelial dysfunction, arterial diseases, extracranial carotid artery disease, a common cause of the transient ischemic attack, termed also “mini-stroke”. Supplementation with vitamin B12, B6, and folic acid resulted in the reduction of homocysteine and oxidative stress (Stroes et al. 2000).

Vitamin D alone but more convincingly in combination with vitamin E decreased oxidative stress in cardiomyocytes, reduced inflammatory markers, and suppressed apoptosis, hypertrophy, and other ailments in obese rats (Farhangi et al. 2017). Vitamin E alone is able to reduce levels of peroxyl radicals and restore cardiac function (Wallert et al. 2019).

Taken together, to obtain more convincing evidence of the beneficial effect of antioxidants on cardiovascular diseases, the evaluation of more representative biomarkers and more clinical trials involving precisely selected target groups have to be conducted.

### Cancer

Results accumulated in the past several decades explored the dual role of ROS in cancer. ROS can either (i) promote protumorigenic signaling supporting the proliferation and survival of cancer cells or (ii) conversely promote antitumorigenic signaling and oxidative stress-induced cancer cell death (Reczek and Chandel 2017).

Compare to healthy cells, cancer cells are characterized by increased formation of ROS and therefore to avoid ROS-induced cell death, cancer cells restore their ROS homeostasis by increasing their antioxidant capacity (Valko et al. 2006). Altered redox homeostasis of cancer cells makes them susceptible to ROS-targeting therapies.

#### ROS-promoting pro-tumorigenic signaling

It has been demonstrated that mammalian cells express three Ras proteins, H-Ras, K-Ras, and N-Ras that promote tumorigenesis following mutational activation (Pylayeva-Gupta et al. 2011). Oncogenic mutations in *Ras* lead to enhanced ROS formation from NADPH oxidase 4 (NOX4) to increase proliferation. ROS are known to hyperactivate PI3K/Akt/mTOR (Phosphoinositide 3-Kinases/Protein kinase B/mammalian Target Of Rapamycin), an intracellular pathway playing an important role in cell cycle regulation, specifically proliferation, cancer, and longevity (King et al. 2015). Hyperactivated PI3K phosphorylates and activates Akt, compartmentalized in the plasma membrane. This pathway is antagonized by several factors, including Phosphatase and Tensin homolog (PTEN) and Protein tyrosine phosphatase 1B (PTP1B). Hyperactivation of PI3K/Akt/mTOR oxidizes and inactivates the phosphatases PTEN and PTP1B (Salmeen et al. 2003). Inactivated PTEN has been found in various human cancers such as prostate cancer, breast cancer, and brain cancer (glioblastoma) (Roa et al. 2015). Activated Akt is even known to further enhance ROS production and thus promote the proliferation and survival of cancer cells. Thus, dysregulation of the PI3K/Akt pathway by excessive ROS production in various cancers is well established and is a critical target in anticancer therapy.

Mitogen-activated protein kinase (MAPK) represents signaling pathways involved in the regulation of various cellular processes such as proliferation, differentiation, stress response, and apoptosis. MAPK pathways activate and phosphorylate downstream proteins (Guo et al. 2020). ROS can oxidize MAPK and hyper-activate MAPK/ERK pro-proliferative signaling (Son et al. 2011).

ERK1 and ERK2 are extracellular, serine-threonine Regulated-Kinases, that regulate cellular signaling of both normal and pathological conditions. Hyperactivation of ERK plays an important role in cancer progression.

The most important signaling cascade of all MAPK signal transduction pathways in the development and survival of tumors is the Ras/Raf/MAPK (MEK)/ERK pathway. ROS-induced dysfunction in the Ras-ERK pathway appears to be a major cause of the incidence of many cancers (García-Gómez et al. 2018). Activated mutations within this pathway represent the most frequent oncogenic factor for many cancers (Khotskaya et al. 2017). Mutations in *ras* and predominantly in *K-ras* (*K*irsten *Ra*t *S*arcoma) are a key trigger for the incidence of cca 30% of all cancers and approximately 10% of all patients suffering cancer. Mutations in *raf* and predominantly in *B-raf* are responsible for 8% of all cancer types and mutations in MEK have a rather low incidence, less than 1% (Sanchez-Vega et al. 2018). Interestingly, the breakdown of mitochondrial respiratory, resulting in the reduction of ROS formation has been shown to suppress tumorigenesis (Weinberg et al 2010).

NF-κB (Nuclear factor kappa B) and Nrf2 (Nuclear factor erythroid 2–related factor 2) play an important role in the regulation of the response to oxidative stress and inflammation (Guo et al. 2020). These transcription factors upregulate the expression of antioxidants in order to suppress cell death mediated by ROS.

Recent results confirmed that Nrf2 has an inconsistent role in cancer. The basal activation of Nrf2 promotes the proliferation of cancer cells via metabolic reprogramming, promotes inflammation-induced cancer, suppresses apoptosis of cancer cells, and recovery of cancer stem cell capacity. In addition, experimental evidence confirmed that Nrf2 works against chemotherapy and radiotherapy (Wu et al. 2019). Overactivated Nrf2 is observed in many cancer cells characterized by high proliferative capacity. The aberrant activation of Nrf2 in cancer cells leads to increased expression of Glucose-6-phosphate dehydrogenase (G6PD), transketolase (TKT), 6-phosphogluconate dehydrogenase (PGD), and other metabolic enzymes promoting glutamine and glucose metabolism in pentose phosphate pathway (PPP) and increasing synthesis of amino acids participating in metabolic reprogramming required for cell proliferation (Mitsuishi et al. 2012).

Nrf2 is important for cell cycle regulation and its deficiency is manifested as G2/M-phase arrest (Reddy et al. 2008). Experiments with Nrf2-deficient mice revealed that elevated Nrf2 promoted phosphorylation of AKT, confirming thus the interference between Nrf2 and PI3K/AKT pathways resulting in increased anabolic pathways in metabolic reprogramming (Mitsuishi et al. 2012). Recently reported results confirmed that Nrf2 plays an important role in maintaining the proliferation of pancreatic cancer cells by regulating mRNA translation (Chio et al. 2016).

Cancer cells are characterized by the ability to escape from the cell apoptosis. Due to the high levels of ROS, cancer cells have expressed high levels of ROS-scavenging enzymes which maintain resistance to ROS-driven cell death. Excessive activation of Nrf2 upregulate antioxidant enzymes necessary for the synthesis of GSH, which counterbalance increased ROS formation and thereby does not enable ROS-mediated cell death. Nrf2 interferes with the escape from cell death by interaction with other signaling pathways such as suppression of proapoptotic Jun N‐terminal kinases (JNKs) (Elsby et al. 2003) and upregulation of antiapoptotic protein B‐cell lymphoma 2 (Bcl‐2) (Niture et al. 2012).

Angiogenesis is constantly activated in cancer. Nrf2 promotes angiogenesis, one of the main features of inflammation-induced carcinogenesis. Heme oxygenase-1 (HO-1) overexpression could be a critical event for angiogenesis. HO-1 expression enhanced the production of vascular endothelial growth factor (VEGF) which mediates angiogenesis manifested by increased proliferation (Meng et al. 2010).

#### ROS-triggered cell death

High levels of ROS have been shown to trigger cell cycle arrest and cancer cell death (Moon et al. 2010). ROS-activation of Apoptosis Signal-regulating Kinase/c-Jun N-terminal kinase/p38 (ASK1-JNK/p38) and Mitogen-Activated Protein Kinase (MAPK), results in cell death. This is proposed to be mediated by TRX1 oxidation by hydrogen peroxide which causes dissociation of ASK1 thereby triggering anti-apoptotic factors via downstream activation of MAPK kinase MKK3/MKK6/p38 and MKK4/MKK7/JNK pathways (Wagner et al. 2009). p38 MAPK has been shown to inhibit oncogenic *Hras* (encode Hras, or Harvey Rat sarcoma virus or transforming protein p21) malignant transformation and ROS- activation of p38 signaling may trigger cell cycle arrest, suppress cell growth and division of cancer cells (Dolado et al. 2007). In addition, higher levels of ROS have been shown to activate death receptors via downregulation of the FLIP—a protein that regulates apoptosis and functions as a key link between cell death and survival pathways (Wang et al. 2008).

Interestingly, buthionine sulfoximine, a chemotherapy-enhancing supplement can inactivate mitogenic signaling pathways driven by Akt in pancreatic cells (Chio et al. 2016). All these results advocate ROS species as tumor-suppressive agents and the role of antioxidants in cell growth and survival of cancer cells (Chio and Tuveson 2017).

In conclusion, the altered redox state of cancer cells represents a potential target for ROS-manipulating anticancer therapies. This topic is discussed below.

#### Anticancer activity of antioxidants 

Cancer progression involves cellular transformation, proliferation, adhesion, migration, and angiogenesis (Zhang et al. 2012). Cancer cells often experience resistance to chemotherapeutical agents and experiments on cellular models proved that antioxidants can increase the sensibility and efficacy of chemotherapy (Guestini et al. 2016).

Antioxidants have been shown to inhibit the kinases such as phosphoinositide-3-kinase (PI3K) and Akt network important in oncogenic survival. In addition, antioxidants may interfere with regulators of cell proliferation such as extracellular signal-regulated kinase 1/2 (ERK1/2), cyclin-dependent kinases regulating the cell cycle (Kim et al. 2006), and transcription factors involving nuclear factor NF-κB.

Antioxidants can inhibit the expression of tumor suppressor proteins p53, a highly mutated transcription factor in cancer, and phosphatase and tensin homolog (PTEN), p21, and p27 (Singh et al. 2017).

A diet enriched with fruits and vegetables or antioxidants in the form of supplements lowered the risk of incidence and mortality in patients suffering from cancer (Genkinger et al. 2004). A low-fat diet accompanied by a high intake of antioxidants has been studied in breast cancer patients. Attention has been devoted to the following variables: tumor recurrence, tumor-specific mortality, and overall mortality. The results of this trial revealed that body mass index (BMI) correlates with overall mortality and a low-fat diet accompanied by a diet rich in vegetables and fruits is linked with a better prognosis (Hauner et al. 2011).

The United States Preventive Services Task Force (USPSTF) reported that the accumulated data on nutritional supplements do not provide unambiguous conclusions for cancer prevention (Moyer 2014). Data obtained from several trials and meta-analysis studies on beta-carotene and vitamin E (ATBC trial) indicate that while vitamin E is not effective in cancer prevention, beta-carotene may even increase the incidence of lung cancer among smokers (see also above).

##### Polyphenols

One of the major sources of polyphenols is tea. Catechins are major constituents of tea of which the most abundant is epigallocatechin-3-gallate (EGCG) capable of inhibiting the growth and development of cancer cells. EGCG has been shown to inhibit the activity of the NF-κB transcription factor in various human cancer cells (Peng et al. 2006). Chemopreventive properties of EGCG have been documented by inhibition of human gastric adenosquamous carcinoma mediated by the inhibition of matrix metalloproteinase induction (Onoda et al. 2011). EGCG as a chemopreventive agent can modulate angiogenesis and apoptosis by downregulation of various signaling pathways such as Vascular Endothelial Growth Factor (VEGF), Epidermal Growth Factor Receptor (EGFR) and cell cycle regulatory proteins such as Mitogen-activated protein kinases (MAPK) (Shankar et al. 2007).

Black tea polyphenols can prevent cancer metastasis by regulating pathways important in the migration of tumor cells involving Matrix Metalloproteinase-2, -7, and -9 (MMP-2, -7, -9) (Patel et al. 2014).

Flavonoids contain several hydroxyl groups in their structure. Due to the high number of hydroxyl groups are able to scavenge ROS formed by the enzymes such as NADPH oxidase, cyclooxygenase, xanthine oxidase, and other enzymes.

One of the most frequently studied flavonoids is quercetin (Jomova et al. 2017). Quercetin exhibits prooxidant properties, especially in the presence of redox-active metals (Jomova et al. 2017). Quercetin can increase the total amount of cellular ROS by redox cycling reactions resulting in the transformation of quercetin to quercetin radical (Q^·^) (Jeong et al. 2009). Quercetin is also able to deplete glutathione and inhibit thioredoxin reductase both important antioxidant systems (Gibellini et al. 2010).

The anticancer activity of quercetin in colorectal cancer has been associated with the activation of cyclooxygenase-2 (COX2) (Raja et al. 2017). Overexpressed COX2 and prooxidant properties of quercetin increasing amount of ROS have been found to be able to mediate apoptosis in a human colorectal adenocarcinoma cell line with epithelial morphology (HC29).

Quercetin has also been found to increase the amount of formed ROS and mediate apoptosis in metastatic SW620 colon cancer cells (Zhang et al. 2017). The main obstacle in using quercetin as a chemopreventive agent is its rather low bioavailability which may be overcome by the application of liposomal formulations.

Luteolin, a flavonoid containing four hydroxyl groups has anticancer properties mediated by oxidative stress suppression. Luteolin has been found to inactivate mTOR signaling in bladder T24 cancer cells, resulting in cell cycle arrest and apoptosis (Iida et al. 2020). Luteolin is able to induce apoptosis in colorectal cancer cells by activating the c-Jun N-terminal kinase (JNK) pathway and Mitogen-activated protein kinase (MAPK) cascades (Kang et al. 2017) and abolishes metastatic potential of squamous cancer cells by inhibition of a non-receptor tyrosine kinase, Src, and pro-metastatic Signal transducer and activator of transcription 3 (STAT3) transcription factor (Fan et al. 2019; Slika et al. 2022).

Apigenin has three hydroxyl groups in its structure and can exert both antioxidant and prooxidant properties. The anticancer effect of apigenin in skin cancer has been attributed to its antioxidant properties via modulation of Mitogen-activated protein kinases (MAPK) and Activator protein-1 (AP-1) in the major cell types of the epidermis, keratinocytes (Hwang et al. 2011). Conversely, apigenin can behave as a prooxidant in various cancer cell types, mainly via inhibition of superoxide dismutase activity and glutathione depletion (Shi et al. 2015). Apigetrin (Apigenin-7-O-glucoside) is more stable than apigenin and has the ability to prevent the progression of the cell cycle and trigger apoptosis in cancers of the gastrointestinal tract (Sun et al. 2018). This effect is achieved by the increased ROS formation (prooxidant effect), resulting in caspase 8 cleavage, inhibition of Janus kinase 2 (JAK2), and triggering regulator of apoptosis BAX, known as bcl-2-like protein 4. Prooxidant behavior of apigenin has been observed by elevated apoptosis in lung, prostate, liver, and bladder cancers (Slika et al. 2022).

Naringenin has three hydroxyl groups and due to its oxidative stress-suppressing properties, this flavonoid exhibits anticancer effects. The antioxidant-protecting effect of naringenin has been reported against liver cancer induced by N-nitrosodiethylamine (Arul and Subramanian 2013). Naringenin halts the formation of the malondialdehyde-DNA complex observed in lung cancer. The prooxidant behavior of naringenin has anticancer potential, for example via the inhibitory effect of glutathione reductase (Yen et al. 2003). Prooxidant properties of naringenin may cause cytotoxicity to various cancer cell lines, apoptosis, and activation of one of the Mitogen-activated protein kinases, ASK1 resulting in apoptosis by ROS-mediated activation of ERK1/2 (Park et al. 2018). Naringenin has properties suitable for its use as adjuvant therapy in cancer treatment. Naringenin used along with chemotherapy allows to overcome resistance to chemotherapy and increase its efficiency.

In conclusion, the majority of flavonoids exhibit antioxidant properties reflected by health-protective functions. Anticancer activity of flavonoids depends on the cell line, bioavailability, dose of flavonoids, level of oxidative stress of the cell, presence of redox-active metals, and other factors. Flavonoids interfere with various signaling pathways important in DNA damage, inflammation, angiogenesis, and metastasis. Since flavonoids can halt cell cycles of cancer cells via several mechanisms, flavonoids are potential candidates for efficient anticancer therapy, or as pharmaceuticals able to overcome resistance to chemotherapy (Luo et al. 2022).

##### Melatonin, carotenoids, vitamins C and E

In addition to polyphenols, other antioxidants such as vitamins C and E, carotenoid lycopene, and melatonin have potential in cancer prevention. Melatonin (see also above) has been shown to reduce the growth of carcinoma cells in aged mice via p21 and apoptosis regulator Bax overexpression, a member of the Blc-2 (B-cell lymphoma 2) family (Xu et al. 2013). In Hepatoblastoma cells HepG2 melatonin was able to trigger apoptosis and suppressed metastasis through Metalloproteinase inhibitor TIMP-1, a sensitive marker of colon metastasis (Nemeth et al. 2011).

One of the biologically most active carotenoids against oxidative damage, lycopene, contains 11 conjugated double bonds and has been reported to have anticancer activities via regulation of oxidative enzymes such as cyclooxygenase-2 (COX-2), 5-lipoxygenase (5-LOX), inhibitory effect on tumor growth and progression, triggering apoptosis, modulation of proteins involved in cell cycle regulation, reduction of the incidence of breast, liver, pancreatic and other cancers (Tang et al. 2012; Kim and Kim 2015).

In the stage of cancer progression, administration of high doses of vitamin C, especially in combination with redox-active metals (copper, iron) may increase the cellular prooxidant state triggering cytotoxicity of cancer cells (Pawlowska et al. 2019).

Vitamin E is known to increase the sensitivity of cancer cells against chemotherapy. Recently has been reported that γ-tocopherol and δ-tocopherol are efficient ROS and RNS scavengers (Sunil Kumar et al. 2017). Inhibition of the NF-κB pathway has been shown to be important in the anticancer activity of a family of unsaturated forms of vitamin E, tocotrienols. In addition, tocotrienols have antiangiogenic properties, blocking tumors from growing their own blood vessels.

N-acetyl cysteine (NAC) is an antioxidant agent with a very good absorbing capacity and the ability to restore glutathione (GSH) (Pedre et al. 2021). NAC is able to reduce cysteine conjugates and has the potential to act as an anticancer agent, however, caution should be paid since NAC has been reported to enhance the metastatic potential of melanoma cells in mice (Piskounova et al. 2015).

#### Antioxidant strategies in cancer treatment

Since oxidative stress is an important component in cancer incidence and progression, oxidative stress modulation is a promising anticancer strategy. In connection with this, antioxidants and weak prooxidants were evaluated in preclinical and clinical testing (Valko et al. 2007). Cancer cells produce significantly higher amounts of ROS than normal cells and therefore administration of prooxidant agents capable of further increase of ROS may represent a potential anticancer approach. For example, exogenous hydrogen peroxide can suppress the survival of a human breast cancer cell line (MCF-7) confirming that prooxidants can enhance ROS-mediated cell death (Bajor et al. 2018). On the other hand, weak prooxidants may trigger the synthesis of antioxidant enzymes and boost the antioxidant capacity of the cell.

Very approximately, therapeutic antioxidant strategies can be classified as (i) application of low molecular weight antioxidants and vitamins including activators of Nuclear factor erythroid 2–related factor 2 (Nrf2), playing an important role in the expression of antioxidant enzymes and (ii) targeting ROS with antioxidant enzymes involving SOD mimetics or NOX inhibitors (Batinic-Haberle et al. 2018).

### Alzheimer’s disease

Alzheimer’s disease (AD) is a multifactorial disease characterized by the degeneration of the cells in the brain (Knopman et al. 2021). This is the main cause of dementia, characterized by cognitive decline. AD has a multifactorial origin; two main hypotheses have been proposed to be responsible for AD, amyloid and cholinergic hypotheses (Butterfield and Halliwell 2019). Research in the past two decades revealed that disturbed homeostasis of redox-active and -inactive metals may also play a role in disease progression. In addition, several risk factors such as high age, head injuries, genetics, and other factors may play a role in the incidence of the disease.

Neuropathology of AD involves the accumulation of senile plaques (protein fragments of amyloid-β, Aβ) and neurofibrillary tangles (NTFs, deposits of phosphorylated protein tau) in the brain. In addition, the degeneration of neuronal cells also occurs.

The nature of the cholinergic hypothesis is based on the suppressed synthesis of the neurotransmitter acetylcholine and therefore the therapeutic intervention is based on the restoration of the physiological level of acetylcholine by inhibiting the activity of enzyme acetylcholinesterase (AChE) (Bartus et al. 1982; Sharma 2019).

Free (unbound) redox metals such as iron and copper can catalyze the formation of ROS, e.g. via the Fenton reaction or by other mechanisms.

At present, two categories of drugs have been approved for the treatment of AD. These involve cholinesterase enzyme inhibitors (natural, synthetic, and hybrids) and N-methyl D-aspartate (NMDA) antagonists.

Various antioxidants of natural and synthetic origin have been found to ameliorate oxidative stress in several models of AD.

#### Alzheimer’s disease and oxidative stress

Another hallmark of AD is the disrupted homeostasis of redox-active (iron and copper) and non-redox (zinc) metals (Jomova et al. 2022). The application of three advanced experimental techniques (proton-induced X-ray emission, Rutherford backscattering spectrometry, and ion microscopy) revealed an increased level of metals in the human tissues containing Aβ plaques compared to surrounding tissues. The levels of copper and zinc have been found to be triple and iron nearly double in comparison with the surrounding tissues (Rajendran et al. 2009). Amyloid-β has a high affinity for charged metals such as copper, zinc, and iron which may, in turn, promote the aggregation of this peptide (Mamelak 2007). Increased levels of redox metals act catalytically and contribute to the formation of damaging ROS and the occurrence of oxidative stress in the brain via the Fenton reaction (Valko et al. 2005)

29 $$\text A\beta \text{Cu}^{{2 + }}  + \text H_{2} \text O_{2}  \to \text A\beta \text{Cu}^{ + }  + 2 \text H^{ + }  + \text O_{2}^{{ \cdot  - }}  $$

30 $$ \text A\beta \text{Cu}^{{2 + }}  + \text O_{2}^{{ \cdot  - }}  \leftrightarrow \text A\beta \text{Cu}^{ + }  + \text O_{2}  $$

31 $$ \text A\beta \text{Cu}^{ + }  + \text H_{2} \text O_{2}  \to \text A\beta \text{Cu}^{{2 + }}  + \;^{ \cdot } \text {OH} + \text {OH}^{ - }  $$

Increased level of oxidative damage in Alzheimer’s brain is documented by the increased protein carbonyls, abundant predominantly in the brain regions rich in Aβ peptide (Hensley et al 1995). Increased peroxidation of lipids in AD or preclinical AD brains or cerebrospinal fluids is proven by enhanced levels of protein-conjugated 4-hydroxy-2-nonenal (HNE), F_4_, and F_2_—isoprostanes (Halliwell 2006). In addition, damage caused by peroxynitrite (ONOO^−^) in AD patients is documented by the increased level of 3-nitrotyrosine (3-NT or 3-NO_2_Tyr) (Smith et al. 1997). A well-known marker of oxidative DNA damage, 8-OH-deoxyguanosine (8-OH-Gua), (see also above) of both nuclear and mitochondrial DNA, has also been found to be increased, which confirms damage caused by hydroxyl radicals (Abolhassani et al. 2017). Increased oxidative/nitrosative stress in the AD brain is also documented by highly abundant oxidized, nitrated, and glycated proteins in amyloid plaques and neurofibrillary tangles.

Amyloid-β plaques can stimulate the NMDA-mediated influx of calcium ions (Zhou et al. 2013). Amyloid-β aggregation is accompanied by ROS formation, including peroxyl radicals, responsible for the lipid peroxidation process and the formation of 4-hydroxynonenal (HNE). It has been proposed that damage caused by ROS and RNS including HNE causes impaired glucose metabolism and electrochemical gradients across membranes which in turn affects calcium homeostasis (Butterfield and Halliwell 2019). Oxidative damage can impair the transcription of the most important genes and lead to the occurrence of mutations. In addition, RNA damage can impair protein translation and impaired synthesis of key proteins.

It appears that the toxicity of amyloid-β depends on the length of the residues. Large amyloid-β42 have low solubility in the lipid bilayer of neuronal cells and exhibit lower toxicity than short oligomers of amyloid-β42, e.g. dimers or trimers, which are toxic to synapses (Yang et al. 2017).

As already mentioned above, the utilization of glucose and oxidative stress occurrence are interconnected. Poor glucose utilization which in turn is responsible for impaired ATP production, frequently leads to suppressed activity of the enzymes important for glucose metabolism. Redox proteomics approach uses mainly mass spectrometry to identify both oxidatively and nitrosatively modified proteins in the brain tissues from advanced-stage or pre-clinical AD patients (Di Domenico et al. 2017). With the help of proteomics, oxidative modifications to glycolytic enzymes, ATP synthase, α-enolase, aconitase (enzyme containing iron-sulfur core), creatine kinase (maintains ATP levels in neurons), and other enzymes have been identified (Butterfield and Boyd-Kimball 2018).

Impaired ATP production in patients suffering from AD has serious consequences, such as disturbed electrochemical gradients across membranes, disturbed functioning of voltage-gated ion channels affecting action potentials and neurotransmission, and increased concentration of intracellular calcium levels. Increased intracellular calcium inhibits enzymes responsible for the distribution of phospholipids in plasma membranes and the release of cytochrome C and apoptosis-inducing factors, triggering neuronal apoptotic death, suppressing neurotransmission, and cognitive dysfunction (Bezprozvanny and Mattson 2018).

#### Activation of mTOR in Alzheimer’s disease

Insulin resistance or impaired insulin sensitivity is typical in AD and can be developed by activation of the mTOR (mammalian target of rapamycin) (Arnold et al. 2018). mTOR is composed of two structurally and functionally distinct forms, mTORC_1_ and mTORC_2_. mTOR is involved in cell proliferation and apoptosis, autophagy, and arthritis by involving various signaling pathways. mTOR signaling pathways are associated with cancer, insulin resistance, and other disorders.

Inhibited autophagy by mTOR activation in Alzheimer’s diseases results in damaged cellular organelles such as mitochondria and accumulation of aggregated misfolded proteins which can cause inhibition of normal cellular processes and finally neuronal cell death (Butterfield and Halliwell 2019). These pathological manifestations have been found in the early stages of AD development as well as preclinical AD.

Studies related to mTOR activation may clarify whether type 2 diabetes is a risk factor for the onset of AD (Tramutola et al. 2015). Another deleterious pathology associated with mTOR activation is insulin resistance which can lead to suppressed glucose metabolism and insufficient ATP production (Di Domenico et al. 2018).

#### NRF2 and Alzheimer’s disease

NRF2 plays an important role in maintaining redox homeostasis, inflammatory response, and cytoprotection against neurodegeneration (Osama et al. 2020). A declined expression of NRF2 and its corresponding genes has been observed in AD brains. Recent studies explored that NRF2 plays a key role in the early stages of AD pathogenesis, involving Amyloid-β. The neuroprotective activity of NRF2 involves antioxidant protection, important is an interference of NRF2 with amyloid-β. Exploration and studies of NRF2 activators against amyloid-β may result in the development of new effective drugs against AD.

#### Antioxidant interventions in Alzheimer’s disease

The extent of oxidative damage observed in Alzheimer’s tissues documents that antioxidants may be helpful in ameliorating oxidative damage (Butterfield and Halliwell 2019). In line with this, various experiments using cell cultures, transgenic mice, and preclinical models revealed protective effects of antioxidants (Polidori and Nelles 2014; Butterfield et al. 1999; Pritam et al. 2022).

##### Vitamin E

is effective in scavenging peroxyl radicals. The biologically most efficient forms of vitamin E are α-tocopherol, which is effective in trapping peroxyl radicals and protecting membranes, and γ-tocopherol, very efficient in deactivating nitrogen-based radicals (RNS) (Pei et al. 2015). Vitamin E has the capacity to delay neuronal death caused by inflammation. Several meta-analyses revealed that patients suffering from AD have decreased levels of vitamin E in plasma (Fata et al. 2014; Dong et al. 2018) and Ginko Biloba and vitamin E extracts improved cognitive functions of the brain (Kandiah et al. 2019). Vitamin E has been shown to reduce the neurotoxicity of the microtubule‐associated protein tau in Drosophila (Cowan et al. 2021).

Despite the promising results of some studies, clinical trials, involving vitamin E therapy in AD patients provided inconsistent results (Winblad et al. 2016; Polidori and Nelles 2014; Martins et al. 2018). This could be due to the limited utilization capacity in the transport and delivery of vitamin E into the brain (Spector and Johanson 2007).

##### Glutathione

 is effective in scavenging lipid peroxidation products such as 4-hydroxynonenal and acrolein as well as in chelating redox metals. Recently has been reviewed that cholesterol-mediated mitochondrial glutathione depletion is accompanied by increased Amyloid-β-induced oxidative stress in mitochondria (Montserrat et al. 2020). It has also been reported that glutathione plays a role in the redox pathway of mitochondrial dynamics regulation in axons (Mariet et al. 2012). Despite these promising works, the exact role of glutathione in AD requires further studies.

##### Vitamin C

The level of vitamin C is higher in the central nervous system than in plasma and positively affects cognitive functions and memory. Vitamin C has been found to be lowered in patients with AD and therefore supplementation of patients with C can have beneficial consequences.

##### Flavonoids

Curcumin is a flavonoid polyphenol with antioxidant, metal chelating (e.g. Zinc), anti-inflammatory and antiaggregating properties (Reddy et al. 2018). Metal-chelating properties of curcumin confirmed protection against Amyloid-β aggregation and oxidative stress suppression, most probably by inhibiting the Fenton reaction (Baum and Ng 2004). Curcumin can reduce oxidative damage, inflammation, and cognitive disturbances in rats exposed to Amyloid-β toxicity (Wei et al. 2006). Curcumin possesses anti-amyloidogenic properties, protects cells against inflammation and oxidative stress, and upregulates Nrf2 (Li et al. 2021). Curcumin can restore glutathione levels and reduce the level of oxidative damage in mice models of AD (Nishinaka et al. 2007). Some studies reported that health beneficial properties of curcumin were difficult to determine, mainly because of its pharmacokinetic and pharmacodynamic properties (Serafini et al. 2017). Despite the promising antioxidant activity and ability of curcumin to enhance the clearance of Amyloid-β obtained from preclinical studies (Nelson et al. 2017), the real value of curcumin in Alzheimer’s therapy remains unclear.

Some flavonoids such as quercetin or resveratrol can partly cross BBB (Schaffer and Halliwell 2012). Quercetin and other flavonoids are known to behave as mild prooxidants resulting in the upregulation of protective genes, such antioxidant enzymes, glutathione, and cytoprotective enzyme Heme Oxygenase HO-1 mediated by activation of the Nuclear factor erythroid 2-related factor 2 (NRF2), a transcription factor upregulating antioxidant response, expression of antioxidant enzymes, and cytoprotective proteins (Ma and He 2012). The mechanism is rather complicated. Xenobiotics usually trigger oxidative stress and may form electrophilic agents preferentially interacting with domains of DNA or proteins or lipids containing high electron density. Enhanced oxidative or electrophilic stress may activate protein kinases able to phosphorylate NRF2 in the cytoplasm which then migrates into the nucleus and interacts with antioxidant response element. The importance of the NRF2 system in Alzheimer’s disease has been documented by the fact that impairment of NRF2 in mouse models resulted in deteriorated cognitive abilities (Perluigi et al. 2006). With regard to flavonoids, the future task is to transfer successful results from animal experiments to humans (Nelson et al. 2017).

When using flavonoids in ameliorating the level of oxidative stress in Alzheimer’s brain, one should be careful since some flavonoids can enhance oxidative stress and behave as prooxidants. For example, quercetin, kaempferol, and catechins have shown under in vitro conditions ability to destabilize amyloid-β and inhibit its formation. It also has been explored that quercetin can interfere with the Nrf2 signaling pathway (Dubey and Singh 2023).

A general feature of many flavonoids is the number of hydroxyl groups and their ability to chelate redox metal ions. Chelated metals have significantly reduced catalytic activity to decompose hydrogen peroxide and form hydroxyl radicals (Fenton reaction). Epigallocatechin gallate (EGCG) is a highly abundant polyphenol in green tea. EGCG has been documented to inhibit oxidative stress and neuroinflammation in AD (Liao et al. 2021).

Resveratrol is a polyphenol found in plants and has been documented to suppress oxidative stress and malondialdehyde levels and restore the level of GSH (Pritam et al. 2022). Resveratrol has also anti-amyloidogenic properties substantiated by the reduced levels of intracellular amyloid-β peptide (Gomes et al. 2018). Resveratrol can also trigger syntheses of antioxidant enzymes such as catalase, SOD, and glutathione peroxidase and protect the mitochondrial membrane from disruption (Arbo et al. 2020). Resveratrol has anti-inflammatory properties and is also able to suppress oxidative stress by compensating for disturbed metal homeostasis in the AD brain (Gomes et al. 2018).

##### Melatonin

Melatonin is an antioxidant with neuroprotective properties and has been found to be lowered in patients with Alzheimer’s disease (Dubey and Singh 2023). In addition to well-known antioxidant properties, melatonin exhibits an anti-amyloidogenic effect. Melatonin stimulates enzymes involved in the synthesis of glutathione.

##### Carotenoids

A number of carotenoids such as lycopene, astaxanthin, and other carotenoids have been shown to have neuroprotective properties through upregulation of Nrf2. Carotenoids are good scavengers of ROS including singlet oxygen and therefore can alleviate oxidative stress in patients with AD.

##### Caffeine

Caffeine is an alkaloid with antioxidant properties and the ability to increase SOD levels in human neuroblastoma cells with amyloid-β. It also has anti-inflammatory properties and phosphorylation-suppressing properties of tau protein in the hippocampus (Machado et al. 2021). Caffeine can inhibit Acetylcholinesterase, slowing down the progression of AD and improving cognitive outcomes.

In conclusion, although various antioxidants have been shown to be effective in ameliorating symptoms of AD, available data from pre-clinical studies are not very convincing to be applied in further studies. In agreement with this, ambiguous results from clinical trials on humans make it very difficult to design a suitable AD treatment. From a practical point of view is very important to optimize the dose size and the critical age of the potential patients at which to start supplementation with antioxidants. Optimization of the dose of antioxidants is important primarily from the point of view of the conditions, e.g. presence of redox-active metals, affecting a switch from antioxidant to prooxidant.

### Parkinson’s disease

Parkinson’s disease (PD) is the second most common neurodegenerative disease and affects 1% of the population over 60 years of age. About 90% of all cases are sporadic, and the remaining 10% of PD cases are inherited. Clinically is PD manifested by motor symptoms, including rigidity, bradykinesia, and tremor (Schapira et al. 2017). Motor symptoms are primarily caused by the death of neurons of the substantia nigra (Damier et al. 1999).

The pathological hallmarks of PD involve depletion of striatal dopamine and axonal degeneration associated with loss of the dopaminergic neurons. Another characteristic sign of the disease is the presence of intracellular inclusions called Lewy bodies, consisting of aggregated α-synuclein, a presynaptic neuronal protein, contributing to Parkinson’s pathogenesis in several ways. Aggregated and deposited α-synuclein is directly associated with the disruption of cellular homeostasis and neuronal death (Jenner 2003). The toxicity of α-synuclein is also responsible for impaired endoplasmic reticulum affecting also closely associated and functionally related Golgi apparatus, aggregation of anti-apoptotic proteins, and formation of pores in cellular membranes (Cooper et al. 2006). Experimental studies using rodents and invertebrates revealed that interventions to suppress aggregation of α-synuclein reduced neurodegeneration and improved motor functions (Lashuel et al. 2013).

The brain is an organ characterized by high, about 20% of the total oxygen consumption of the body. Therefore the brain is a major source of radicals derived from oxygen (Johnson et al. 2012). ROS are formed in the brain from several sites, the most important being neurons and glia. The main ROS flux comes from the mitochondrial electron transport chain, other sources are NADPH oxidase and flavoenzymes, as well as nitric oxide.

Analysis of oxidative stress markers revealed that dopaminergic loss of neurons is mediated by ROS derived from the metabolism of the neurotransmitter dopamine associated with low levels of glutathione and high levels of essential metals calcium and iron in substantia nigra pars compacta. In addition, the brain is rich in polyunsaturated fatty acids and a significant level of damage originates from lipid peroxidation and oxidative products such as carcinogenic 4-hydroxynonenal and potentially mutagenic malondialdehyde (Liu et al. 2008; Dias et al. 2013).

ROS-induced damage of lipids significantly affects membrane fluidity and permeability. Oxidative stress frequently affects cysteine and selenocysteine protein residues. DNA damage has been documented by the increased level of radical adducts with guanine (Su et al. 2019). ROS levels directly influence synaptic efficiency, characterized as the most important origin of neuronal dysfunction in PD.

α-Synuclein has been reported to cause dysfunction of mitochondrial complex I in PD which in turn increases levels of ROS and consequently ROS-induced oxidative stress (Winklhofer and Haass 2010). The onset of PD is associated with severely decreased levels of reduced glutathione (GSH) in the substantia nigra. GSH depletion has been confirmed to precede the dysfunction of mitochondrial complex I and lowered dopamine levels. Boosting mitochondrial GSH (mGSH) in the brain may have beneficial and neuroprotective effects in humans.

More than half a decrease in mitochondrial I activity has been observed in patients with sporadic PD and parkin mutations. Reduced activity of complex IV was observed only in sporadic PD patients (Moon and Paek 2015). Increased levels of oxidized proteins and nitrated tyrosine residues within the α-synuclein have been found in Lewy bodies in patients with PD. It has been reported that in glutathione-depleted dopaminergic cells, prevention of mitochondrial complex I inhibition can be achieved by the application of nitric oxide synthase inhibitors (NOS) reducing thus the formation of nitric oxide (Chinta et al. 2007).

#### Role of dopamine in Parkinson’s disease

The above-described biochemical disturbances result in dopamine oxidation to dopamine-quinones or 6-hydroxydopamine, both involved in neurodegeneration (Hastings 2009). Dopamine-derived quinones (DA-Q) can undergo intramolecular cyclization and oxidation to form leukochrome, aminochrome, and finally, neuromelanin, a pigment involved in neuroinflammation and neurodegeneration (Zecca et al. 2008). Aminochrome is a highly reactive molecule capable of forming adducts with α-synuclein and ROS such as superoxide radical anion and depleting NADPH (Norris et al. 2005).

Observation of cysteinyl adducts of dopamine in substantia nigra postmortem samples confirmed severe oxidation process occurring in PD brain. (Spencer et al. 1998). Dopamine transport, utilization, and storage may be involved in increased ROS formation followed by cellular dysfunction. Newly synthesized dopamine is sequestered in vesicles via active transport with the involvement of vesicular monoamine transporter 2 (VMAT2), which is a regulator of cytoplasmic levels of dopamine at physiological levels preventing ROS-induced oxidative stress.

#### Role of iron and calcium in Parkinson’s disease

Similarly to other neurodegenerative diseases, redox metals play important roles also in PD. Increased levels of iron have been confirmed in the PD midbrain, which in turn results in iron-induced oxidative stress and neurodegeneration (Chinta and Andresen 2008). Chelated iron exhibits suppressed catalytic activity (lower number of free Fe binding sites, lower catalytic activity) in ROS formation (e.g., by Fenton reaction, see above) and slowing down PD progression. Clioquinol is an antifungal drug able to chelate iron forming Fe-clioquinol complex. Chelated iron prevents ROS-induced damage to dopaminergic neurons (Kaur et al. 2003).

Ferritin is an intracellular iron protein that stores and releases free iron (in ferrous form) in a controlled way. It has been declared, that neurotoxic 6-hydroxydopamine (and also superoxide radical) is able to release “free iron” from ferritin which in turn may participate in Fenton chemistry, forming ROS and initiating lipid peroxidation (Monteiro and Winterbourn 1989).

Calcium is another essential metal involved in the pathogenesis of PD. Intracellular regulation of Ca^2+^ is maintained by the action of ATP-dependent pumps, which trigger mitochondrial activity and accompanying enhanced ROS formation (Surmeier et al. 2011).

Compare to other brain regions, dopaminergic neurons in the substantia nigra pars compacta are highly susceptible to the regulation of Ca^2+^ homeostasis. For example, nigral neurons are less abundant with the L-type Ca^2+^ channels and are characterized by low oscillations of Ca^2+^, more expressed calcium-binding proteins calbindins, and therefore exhibit lower susceptibility to damage in PD (Khaliq and Bean 2010). These results suggest that the severe fluctuations in calcium signaling increase the susceptibility of substantia nigra neurons to damage and cell death.

#### Parkinson’s disease-related proteins and oxidative stress

The most important genes associated with familial PD involve α-synuclein (discussed above), DJ-1, Parkin, PINK-1, and others. The discovery of these genes resulted in the clarification of molecular pathways and the involvement of oxidative stress in the pathogenesis of PD.

DJ-1 is a neuroprotective protein involved in the regulation of antioxidant, anti-inflammatory and antiapoptotic pathways (Kahle et al. 2009). DJ-1 is expressed in neurons and glial cells and acts as a ROS scavenger and transcriptional co-activator and molecular chaperon (Yokota et al. 2003). DJ-1 exhibits protective activity against hydrogen peroxide, 6-hydroxydopamine, and rotenone. Generally, the protective effect of DJ-1 against the deleterious effects of ROS is achieved by several mechanisms. DJ-1 exposed to oxidative stress undergoes oxidation of Cysteine106 and translocates from cytoplasm to mitochondria or to the nucleus (Junn et al. 2009). In fact, oxidation of Cystein106 to cysteine sulfinic acid is necessary for triggering the cytoprotective activity of DJ-1; oxidation at different residues leaves the protein inactive.

One of the most important PD-associated genes is PINK1 (PTEN-induced putative kinase 1), encoding PTEN-induced serine/threonine kinase 1, and PRKN, encoding an E3 ubiquitin ligase Parkin. PINK1 mutations disturb kinase functions and neuroprotection. Mitochondria are key targets for PINK1 functioning, an important regulator of mitochondrial respiration, phosphorylation of chaperons, and other functions (Yonova-Doing et al. 2012).

Experimental studies employing cell lines revealed that PINK1 deficit leads to swelling, shortening (Dagda et al. 2009), and fragmentation of mitochondria as a result of inhibited Complex I enzyme activity (Gegg et al. 2009). Knocked down PINK1 in SHSY5Y cells results in inhibition of mDNA synthesis, disruption of key indicators of mitochondrial activity, mitochondrial membrane potential, and suppressed production of ATP (Gegg et al. 2009). Neuronal cells with overexpressed wild-type PINK1 protect against an indolocarbazole alkaloid Staurosporine-triggered release of mitochondrial cytochrome c and apoptosis. Mutated PINK1 is characterized by severe oxidative stress-induced cell death (Chien et al. 2013; Dias et al. 2013).

Parkin (encoded by the PRKN gene) plays a role in the degradation of unwanted proteins. Under normal conditions, Parkin is localized in the cytosol, however, oxidative stress causes its translocation to mitochondria. In the cytoplasm and nucleus, Parkin functions as an E3 ubiquitin ligase. Loss of Parkin activity due to the mutations has been related to autosomal recessive early onset of Parkinson’s disease (Zhang et al. 2000). Various studies confirmed an association between Parkin and oxidative stress. In vitro study employing the SH-SY5Y cell line is one of the most frequently studied models for PD. This cell line with overexpressing wild-type and mutant Parkin exhibits decreased levels of ROS and resistance to apoptosis induced by 6-hydroxydopamine (Jiang et al. 2004). Conversely, expressed mutant Parkin results in increased levels of carbonyls, peroxidation of lipids, and nitrated proteins (Hyun et al. 2002).

#### Antioxidants and Parkinson’s disease

##### Coenzyme CoQ10

 (known also as ubiquinone) is occurring in almost all cells. It is an important component of oxidative phosphorylation in mitochondria. Its location close to the membranes protects them from free radicals-induced damage and prevents peroxidation of lipids and DNA damage (Crane 2001). The dietary intake and bioavailability of coenzyme Q10 are low and therefore it has been recommended to be administrated by supplements (Duarte-Jurado et al. 2021).

Significantly decreased levels of CoQ10 have been detected in the cortex of post-mortem brain tissues of Parkinson’s brains. In addition, the level of the oxidized form of ubiquinone has been found to be increased compared to healthy subjects (Sohmiya et al. 2004). In vitro PD models confirmed the protective effect of CoQ10, showing significantly reduced loss of dopaminergic cells (Gille et al. 2004). These findings have been confirmed in primates supplied daily by cca 19 mg/kg for 10 days (Horvath et al. 2003).

In the past two decades, several clinical trials targeted at the potential beneficial effects of CoQ10 in patients with PD have been conducted. Oral supplementation of 200 mg (which is considered a safe dose) for three months did not bring any positive outcomes (Strijks et al. 1997). In the next trial, Parkinson’s patients in the early stages of the development of the disease, supplemented with 1200 mg per day (a safe and well-tolerated dose) delayed the progression of deterioration of the disease (Shults et al. 2002).

##### Glutathione

Post-mortem brains of Parkinson’s patients revealed the altered ratio between the reduced and oxidized glutathione (GSH/GSSG ratio) and the reduction of glutathione in the substantia nigra (Sian et al. 1994).

Both in vitro and in vivo experiments confirmed that restoring levels of glutathione in PD patients represents a promising strategy to slow down the progression of the disease (Pradhan et al. 2020). PD patients were administrated with glutathione (two 600 mg doses twice a day for 30 days) intravenously (to avoid glutathione degradation in the gastrointestinal tract) (Sechi et al. 1996). GSH therapy improved the performance of patients, with nearly half a decline in disability of patients which lasted for about three months after interrupted therapy. Based on these findings, it has been proposed that GSH slows down the disease progression.

In another study, PD patients with badly controlled motor symptoms were intravenously administrated 1400 mg of glutathione three times a week for four weeks. The outcome was a minor symptomatic improvement and well-tolerated glutathione (Hauser et al. 2009). Regardless of the limited success shown in these two studies, further investigations with more subjects and optimized doses to observe more significant improvements in patients are necessary to conduct.

##### N-acetylcysteine

N-Acetylcysteine has been used for many years in clinical praxis to restore hepatic levels of glutathione after acetaminophen overdose (Smilkstein et al. 1988). N-Acetylcysteine is an efficient antioxidant with good ROS, RNS, and thiyl radical scavenging activity and the detoxification of semiquinones, heavy metals, hypochlorous acid (HOCl), and other oxidants. NAC has the potential to restore GSH levels and improve symptoms in PD, however, its bioavailability is rather low, reaching cca 7%. In a trial with patients suffering from PD was glutathione (GSH) level quantitatively monitored by Magnetic Resonance Imaging (MRI) following intravenously administrated 150 mg/kg of NAC. In all studied subjects the level of GSH increased, however, returned back to its original level after 120 min (Holmay et al. 2013).

Individuals were taking 3000 mg of NAC every day for four weeks. The results have shown that the level of GSH/GSSG increased, however, the biomarkers of oxidative damage (peroxidation of lipids) remained unchanged (Coles et al. 2018).

In a very recent study, 42 patients with PD were intravenously infused with NAC (50 mg/kg) plus taking NAC orally (500 mg twice a day) for 3 months. Dopamine transporter binding was evaluated by using DaTscan imaging (DTscan is a radiopharmaceutical used in a brain scan (SPECT), to visualize striatal dopamine transporters). Improvement in dopamine transporter binding (cca 3.5–8.3%) suggests that NAC has positive clinical effects and improved symptoms in patients with PD (Monti et al. 2019).

##### Carnosine

Carnosine is a dipeptide (β-alanyine-L-histidine) with metal-chelating (contains several nitrogen donor atoms), antioxidant, reducing, and radical scavenging properties, mainly peroxyl radicals causing damage to biological membranes (Kohen et al. 1988).

Carnosine is known to restore intracellular ATP depleted by 6-hydroxydopamine (6-OHDA) in a PD model. Accelerated senescence in animal models has been decelerated by carnosine administration accompanied by decreased activities of Monoamine oxidase (MAO, a flavin-containing mitochondrial enzyme) and Na^+^/K^+^-ATP-ase in synaptosome (Yuneva et al. 2002).

Parkinson’s patients with trembling manifestations taking basic treatment with Levodopa and dopamine-receptor agonists plus oral carnosine supplementation showed enhanced efficiency of basic therapy against PD, manifested by restored levels of superoxide dismutase, typically lowered in patients (Boldyrev et al. 2008).

##### Vitamins C and E

The brain is an organ with the highest abundance of vitamin C, however, different brain regions have different abundance. Patients suffering from neurological disorders such as PD have been reported to have decreased plasma levels of vitamin C; the level of vitamin C can be considered a biomarker of PD progression (Foy et al. 1999). Thus vitamin C supplementation may be a potential therapeutic agent to improve symptoms of PD. Unfortunately, clinical trials using vitamin C were not reproducible.

Dietary intake of vitamin C has been connected with a reduced risk of PD, however, two large cohort studies did not confirm this expectation (Hughes et al. 2016).

Concurrent intake of high doses of vitamin C and vitamin E (α-tocopherol) delayed the onset of treatment for more than 2 years in newly diagnosed PD patients (Fahn 1992).

Vitamin C has been shown to enhance levodopa bioavailability and kinetics of absorption in patients with PD (Nagayama et al. 2004). However, vitamin C does not reduce the incidence of PD.

Preclinical studies with vitamin E provided controversial results. Systematic administration of vitamin E did not show a protective effect in laboratory animals including the inability to attenuate dopamine depletion (Martinovits et al. 1986; Gong et al. 1991). Taken together, vitamin E did not provide any significant protection against the onset and progression of PD.

### Oxidative stress and mental and psychiatric disorders

The brain is rich in lipids and has a high partial pressure of oxygen, therefore similarly to neurological diseases, oxidative stress has been implicated in various mental and psychiatric disorders, including behavioral, emotional, and cognitive abnormalities, such as anxiety, depression, schizophrenia, bipolar disorder and other diseases (Ng et al. 2008; Salim 2014). Although many detailed studies have declared an association between mental/psychiatric diseases and oxidative stress, the causal relationship between them is problematic to reliably determine. In fact, it is difficult to determine, if oxidative stress is a cause or a consequence of the disease’s state.

### Down syndrome

Down syndrome is due to trisomy 21, the most common chromosomal abnormality occurring in humans. Of 500 genes on chromosome 21, 18 genes influence gene expression, and more than 20 genes are involved in metabolic functioning (Sturgeon and Gardiner 2011). Several genes of chromosome 21 are directly implicated in oxidative stress, involving Cu, Zn-SOD (SOD1), ETS2 (ETS Proto-Oncogene 2) transcription factor, stress-inducible factor, protein S100, Down syndrome critical region gene 1 (DSCR1), amyloid precursor protein (APP), the β-site APP cleaving enzyme 1 (BACE1) (Antonarakis et al. 2004; Helguera et al. 2005).

Oxidative stress in Down syndrome significantly contributes to the cognitive defects of individuals with Down syndrome. Oxidative stress negatively affects brain development which in the later stages accelerates aging and the development of Alzheimer’s disease. At about 40 years of age, practically all patients with Down syndrome exhibit neuropathological changes typical for Alzheimer’s disease including β-amyloid plaques and neurofibrillary tangles (Mann and Esiri 1989). The first signs of β-amyloid plaques appear already in childhood at the age of eight or earlier (Lott and Head 2005). The peak of the rate of β-amyloid deposition reaches between 35 and 45 years of age.

The neuropathology of Down syndrome is tightly linked with oxidative stress. Cortical neurons are the main blocks responsible for cognition. Analysis of such cortical neurons from fetuses, confirmed significantly increased levels of ROS responsible for apoptosis (Busciglio and Yanker 1995). The analysis confirmed the occurrence of glycation end products and that the process of glycoxidation is triggered very early in prenatal life (Head et al. 2001).

Interestingly, it has been observed that the deposited β-amyloids within plaques have signs of oxidative modifications of Aβ in the brains of individuals with Down syndrome. As already discussed above, mitochondria contain enzymes for ATP production, apoptotic signaling, ROS formation, and other processes (Santos et al. 2010). Dysfunction of mitochondria and increased levels of oxidative stress in the brain, neuronal cell cultures, urine, and erythrocytes have been confirmed in individuals with Down syndrome (Jovanovic et al. 1998).

The role of uric acid in individuals with Down syndrome is controversial. Whereas one study employing children with Down syndrome confirmed increased levels of uric acid and antioxidant capacity, in another study increased levels of uric acid were associated with increased oxidative stress markers, as documented by elevated allantoin, a major metabolic intermediate produced from uric acid (Tiano et al. 2008). Thus the exact role of the mutual link between uric acid and oxidative stress requires further study.

#### Antioxidant therapy and Down syndrome

Since oxidative stress is one of the most important neuropathological processes contributing to cognitive deficits in Down syndrome. Thus therapeutic interventions reducing signs of oxidative stress appear to be a promising strategy to improve cognitive performance in patients with Down syndrome (Lott 2012). Several antioxidants have been shown to be effective in preclinical studies, however, clinical studies did not confirm the effectiveness of antioxidant therapy in cognitive improvement. Although supplementation with antioxidants in patients with Down syndrome improves oxidative stress biomarkers and the overall antioxidant status of an organism. However, no improvements in cognitive performance or retardation of cognitive decline have been observed (Rueda Revilla et al. 2020). Below are briefly discussed some trials monitoring the effectiveness of antioxidant therapy in patients suffering from Down syndrome.

In vitro and in vivo Down syndrome models revealed that supplementation of mice with low molecular weight antioxidants such as α-tocopherol reduced the level of oxidative stress, attenuated degeneration of cholinergic neurons, and improved working memory (Lockrow et al. 2009). Similarly, the hormone melatonin is a potent ROS scavenger and regenerator of damaged molecules (Tan et al. 2015). Long-term administration of melatonin to mice reduces cholinergic degeneration and the number of senescence cells in the hippocampus (Parisotto et al. 2016).

Based on these promising studies, various trials using antioxidants in adults with Down syndrome have been conducted. In one of the trials lasting 2 years, antioxidant supplementation with vitamin C, vitamin E, and α-lipoic acid in subjects with Down syndrome did not prevent cognitive decline nor improved cognitive function (Lott et al. 2011).

Children and teenagers (7–15 years of age) with Down syndrome supplemented with vitamins E and C or α-lipoic acid explored suppressed signs of oxidative damage. Vitamins C and E were able to suppress lipid peroxidation in the blood (Parisotto et al. 2014). Oxidative damage has been found to be suppressed even 6 months after the discontinued treatment. From this follows that longer treatment with higher doses of vitamins C and E is beneficial in reducing oxidative damage to lipids, however, without cognitive improvements.

Attention has also been paid to an essential micronutrient selenium possessing antioxidant properties mainly via regulation of the activity of antioxidant enzymes such as glutathione peroxidase (GPx). Unexpectedly, supplementation of children (from 6 months to 16 years of age) with selenium over a period of 6 months caused the reduced effect of GPx in erythrocytes (Anneren et al. 1989). A positive outcome of this trial was that selenium supplementation was well tolerated and no side effects were observed.

Based on the various trials attempting to monitor the beneficial effect of antioxidant supplementation in patients suffering from Down syndrome, it can be concluded that although vitamins were able to improve oxidative stress markers, cognitive functions were not improved. From this follows that the increased level of oxidative stress is not a key factor responsible for intellectual disability.

### Depression

Depression is one of the most commonly occurring civilization disorders in the twenty-first century (Cecerska-Heryc et al. 2022). There are several types of depression sharing several common symptoms such as sadness, emptiness, and irritability. Depression also causes cognitive decline. Depression has a very high rate of suicides (Bains and Abdijadid 2021).

It is believed that depression is caused by disturbed levels of neurotransmitters, mainly serotonin, norepinephrine, and dopamine. People prone to commit suicide have lower levels of serotonin than normal people. Since serotonin modulates other neurotransmitters, serotonin fluctuations may have serious consequences resulting in disturbed neurotransmission. For example, lower serotonin levels may affect concentrations of norepinephrine (noradrenaline) to lower levels which in turn may trigger depression (Balmus et al. 2016).

Recently, it has been proposed that depression is caused by the concerted action of neuroregulatory systems with secondary changes. In the development of depression are involved gamma-aminobutyric acid (GABA), glycine, and glutamate (excitatory neurotransmitters). GABA has been found to be lowered in the plasma and cerebrospinal fluid in patients with depression (Siwek et al. 2013). Glutamate, as one of the major excitatory neurotransmitters responsible for memory, learning, and synaptic plasticity is regulated by the glutamatergic system. Under stress conditions, the glutamatergic system releases unusually high amounts of glutamate into the synapses, which in turn is responsible for neurotoxicity, hyperactivity, and eventually cell death. High concentrations of glutamate are known to induce ROS formation by certain enzymes, for example, xanthine oxidase, resulting in oxidative damage. The high amount of released glutamate causes decreased concentration of gamma-aminobutyric acid (GABA) confirmed by MRI localized spectroscopy of brain metabolites. A lower concentration of GABA correlates with the acute phase of depression.

Depression is accompanied by observation of oxidative DNA damage, namely 8-OH-Gua (see above) confirmed by the analysis of plasma, urine, and leucocytes of patients with depression (Black et al. 2015).

#### Depression and antioxidants

Several studies reported correlations between consumption of flavonoids and depression. A study from Brazil reported that women suffering from depression consumed smaller amounts of polyphenols than healthy women (de Oliveira et al. 2019). In another study, a group of 50 volunteers for 8 weeks consumed dark chocolate and blueberries (Kontogianni et al. 2020). Increased intake of flavonoids resulted in reduced symptoms of depression.

One of the most abundant flavonoids in fruits and vegetables is quercetin (Jomova et al. 2017). Quercetin can cross the blood–brain barrier and regulate neurotransmitters. Experiments using mice have shown that quercetin is able to make a protection against anxiety and depression as well as improve memory by regulating mutually interacting cholinergic and serotonergic systems (Samad et al. 2018).

Intake of quercetin combined with physical activity exerts an antidepressant effect by suppressing inflammatory pathways and regulating the brain-derived neurotrophic factors (BDNF). BDNF plays an important role in the survival and growth of neurons and acts as a modulator of neurotransmission (Sadighparvar et al. 2020).

Food of rats fortified with quercetin and curcumin with added carrageenan, a sulfated polysaccharide extracted from seaweeds, resulted in modulation of expression of heme oxygenase-1 (HO-1) mRNA and Tumor Necrosis Factor-α (TNF-α), showing anti-inflammatory activity and oxidative stress suppressing effect of these substances (Heeba et al. 2014; Winiarska-Mieczan et al. 2023).

Curcumin is a biologically active compound derived from Curcuma longa. Curcumin has been considered a potent antidepressant affecting monoaminergic systems including dopaminergic, serotonergic, noradrenergic, and histaminergic circuitries. Curcumin reduced the expression of inflammatory cytokines IL-1 and TNF, vascular cell adhesion molecule-1 (VCAM-1) an important cell adhesion molecule involved in inflammation and inflammatory leukotrienes and prostaglandins (Lamanna-Rama et al. 2022).

Curcumin is known to suppress the synthesis of various enzymes such as Mitogen-activated protein kinases (MAPK), cyclooxygenase (COX), lipoxygenase (LOX), Nuclear factor kappa B (NF-κB), and others (Panaro et al. 2020).

An approximately 100 g increase in intake of fruit or vegetables reduces the risk of depression by about 3% (Saghafian et al. 2018). A diet containing high content of phenols, anthocyanins, and vitamin C improves symptoms of depression mainly by reducing the level of oxidative damage (biomarkers) in the brain, increasing levels and activity of antioxidant enzymes such as SOD and GPx, and increasing the level of expression of glutathione (Dong et al. 2021).

Vitamin C deficiency and its association with psychiatric effects have been known for many years. Vitamin C deficiency is responsible for adverse mood and cognitive defects. This is due to the importance of vitamin C in modulating the synthesis of neurotransmitters (Plevin and Galletly 2020).

A cohort of 129 women aged 60–90 years of age diagnosed with depression were divided into two groups, based on the serum level of vitamin C: the low-level group (serum level of vitamin C < 23 µM, *n* = 27) and the group with higher serum level of vitamin C (> 23 µM, *n* = 102). Depression score was significantly higher in the group with low serum levels of vitamin C, showing the importance of plasma level of vitamin C in depression attenuation (Marazzi et al. 1990).

### Schizophrenia

Schizophrenia is a serious psychiatric illness, whose etiology is not satisfactorily described. Among other pathologies, impaired neurotransmission, autoimmune dysfunction, abnormal neuronal development, and oxidative stress have been proposed (Kessler 1997; Salim 2014).

The importance of oxidative stress in schizophrenia has been proposed already in the middle of the twentieth century (Hoffer et al. 1954). Since diagnosed patients exhibit increased levels of ROS damage and suppressed antioxidant protection, it appears rational, that the role of oxidative stress in schizophrenia has solid ground in the etiology of this disorder. In addition, the genetic examination found an association between polymorphism of genes and oxidative stress markers in schizophrenia (Cecerska-Heryć et al. 2022).

Dopamine is an inhibitory neurotransmitter and its abnormalities in the prefrontal and mesolimbic brain regions have been found in patients with schizophrenia. It has been proposed that dopamine may play a role in the pathogenesis of schizophrenia. However, recent results confirmed that glutamate, acetylcholine, serotonin, and GABA changes may also contribute to the pathology of the diseases.

Symptoms of schizophrenic patients are becoming more serious when oxidative stress markers exhibit a proportional increase (Patel et al. 2014). It has been proposed that redox imbalance may result in interneuron deficiency and schizophrenia phenotypes. The disturbances in interneurons may affect normal cognitive functions of the brain.

The white matter of the brain contains large amounts of lipids prone to peroxidation and therefore is susceptible to loss of the myelin sheath in brains. Peroxidation of lipids affects the membrane fluidity of neurons and consequently the transport of neurotransmitters (Barber and Raben 2019). Chronically ill patients of all subtypes of schizophrenia have increased levels of malondialdehyde and thiobarbituric acid reactive substances (TBARS) in blood cells (Khoubnasabjafari et al. 2015).

F2-isoprostanes are ROS-mediated products of arachidonic acids and are very sensitive markers of the lipid peroxidation process (Dietrich-Muszalska and Kontek 2010). Increased levels of isoprostanes, and predominantly 8-iso-prostaglandin (8-iso-PGF2α) have been detected in the urine of patients, making analysis of isoprostanes a valuable and reliable marker of the level of oxidative damage in schizophrenia patients (De Simone et al. 2023).

Several studies confirmed severe antioxidant deficits in chronic schizophrenia patients, for example, the aberrant activity of SOD (Zhang et al. 2003a; Wu et al. 2013). The activity of antioxidant enzymes in schizophrenia patients measured in erythrocytes provided contradictory results (Smaga et al. 2015). The enzyme activity depends on age and therefore older patients may exhibit lower enzyme activity. SOD is the first line of antioxidant defense and the data shows increased, decreased, or constant activity of this enzyme. For example, patients with schizophrenia taking antipsychotic drugs have increased levels of SOD activity (Padurariu et al. 2010). This denotes a negative correlation between positive symptoms (e.g. hallucinations) and SOD levels (Zhang et al. 2012). Significantly higher level of SOD has been observed among chronically ill patients taking antipsychotic drugs than in patients in the first episode. This observation indicates that the level of oxidative stress occurs mainly in the first episode characterized by fewer symptoms (Zhang et al. 2012).

Plasma glutathione peroxidase levels have been found to be lowered in schizophrenia patients (Gawryluk et al. 2011) as well as in psychotic children (Golse et al. 1977).

However, certain differences in antioxidant status between subtypes of schizophrenia such as most common paranoid schizophrenia and catatonic schizophrenia, disorganized schizophrenia, and other subtypes have been observed (Yao et al. 1999).

#### Antioxidant therapy in schizophrenia

Three groups of treated and untreated patients with schizophrenia and healthy controls were analyzed for the content of antioxidant enzymes and low molecular weight antioxidants. It was found that the enzyme glutathione peroxidase (GPx) was significantly lowered in both groups of patients. Untreated patients revealed decreased levels of catalase (CAT), glutathione (GSH), and albumin. Both groups of patients, treated and untreated showed significantly lowered non-enzymatic antioxidant capacity. Antioxidant status was most significantly impaired in untreated patients, confirming that the level of antioxidants in patients with schizophrenia is tightly related to the suppression of pathological manifestations of the disease (Goh et al. 2022).

Supplementation with high doses of vitamins C and E and fish oil has been found to improve clinical symptoms of patients with schizophrenia and partially restored levels of antioxidants (Pandya et al. 2013). In addition, ginkgo has been found to positively affect the symptoms, most probably via improving antioxidant status.

Curcumin has strong antioxidant, antimutagenic, anti-inflammatory, and antimicrobial functions and has been found to reduce the symptoms of patients with schizophrenia (Rabiee et al. 2023). Curcumin can pass the blood–brain barrier and prevent neurological and behavioral disorders, preserve lipid homeostasis and minimize the side effects of antipsychotic therapy.

A systematic review and meta-analysis of N-acetylcysteine in schizophrenia patients have been carried out (Kishi et al. 2023). Both subgroups, individuals in the acute phase and in the non-acute phase were supplied with N-acetylcysteine. Individuals with schizophrenia in the acute phase were most responsive to the treatment with N-acetylcysteine than those who were not in the acute phase. However, considerable heterogeneity across the subgroups has been noted.

Vitamin C is known to affect the dopaminergic system and its deficiency has been associated with schizophrenia (Plevin and Galletly 2020). Various studies reported that chronic psychiatric patients, for example with schizophrenia, require much higher doses of ascorbic acid to excrete the normal content of vitamin C (Sandyk and Kanofsky 1993). One month of supplementation with 70 mg of vitamin C resulted in the urinary content of vitamin C being significantly lower in schizophrenic patients than in neurotic patients (Suboticanec et al. 1986). Therefore patients with schizophrenia should regularly take higher amounts of vitamin C (minimum 2 g/day) to reach normal plasma levels of vitamin C.

The role of vitamins C and E in the management of schizophrenia has been studied by randomized control trials (Kouser et al. 2021). 105 patients were divided randomly into three groups, group 1 taking daily 500 mg of vitamin C, group 2 receiving 600 IU of vitamin E twice daily, and group 3 taking placebo on a daily basis. The results have shown that the supplementation of patients suffering from schizophrenia with vitamins C and E and antipsychotic drugs are effective in the management of schizophrenia and in the alleviating of symptoms typical for schizophrenia. Vitamin C supplementation in schizophrenic patients has been reported to normalize not only plasma levels of ascorbic acid but also malondialdehyde abnormalities (Dakhale et al. 2005). Other studies reported that supplementation of patients with schizophrenia with vitamins C and E and omega-3 fatty acids provided positive results in terms of extrapyramidal side effects (Sivrioglu et al. 2007).

Ginkgo Biloba is rich in antioxidants and has many health benefits. Ginko Biloba is frequently used to treat mental health conditions. Patients with schizophrenia on medical treatment were supplemented with extracts from Ginko Biloba (Zhang et al. 2001). The outcome was very promising as measured by a higher response rate and significant reduction score on the scale of assessment of positive symptoms. In addition, increased levels of superoxide dismutase have been reported among schizophrenia patients resistant to medical treatment (Zhang et al. 2003b).

### Bipolar disorder

Bipolar disorder (BD) or manic depression is a mental health disorder characterized by extreme swings in mood with emotional highs (mania or hypomania) and lows (depression) (Andreazza et al. 2008). The incidence of bipolar disorder is between 1 and 3% of the world’s population with average age between 20 and 30 years. There are two types of bipolar disorder, Type I involves severe episodes of mania and depression, and Type II includes episodes of hypomania and depression (Andreazza et al. 2008; Cecerska-Heryc et al. 2022).

Evaluation of oxidative stress markers in patients with bipolar disorder revealed their increased values in serum and erythrocytes (Akarsu et al. 2018). As expected, the highest markers of oxidative stress, including markers of oxidative DNA damage have been observed during the manic episode. Post-mortem analysis of the prefrontal cortex showed significantly increased levels of 8-OH-Gua (see also above) which may in turn affect the aggregation of proteins (Balmus et al. 2016). Interestingly, oxidative DNA damage and telomere shortening have been found to be increased in patients with bipolar disorder which in turn has been estimated to accelerate aging by about 10 years (Akarsu et al. 2018).

Plasma protein carbonyls is another oxidative stress marker that has been found to be increased during manic and depressive phases in patients with bipolar disorder (Andreazza et al. 2010; Yager et al. 2010).

It has been reported that DNA oxidative damage is related to increased telomere shortening in patients with BD; this translates into an approx. 10-year acceleration of aging (Akarsu et al. 2018). In a post-mortem study of the hippocampus, it was proved that damage to the genetic material in this region occurs in the cytoplasm of cells, so it affects RNA more than DNA (Palta et al. 2014). It has also been suggested that oxidative modifications of DNA and RNA may affect the heredity of BD (Brown et al. 2014).

It is well known that proteasomes (complex intracellular proteases) are able to mildly degrade oxidized proteins (Dunlop et al. 2009) and therefore protein oxidative damage can be considered temporary. The exact role of protein damage in the etiology of bipolar disorder is still under investigation (Brown et al. 2014).

Peroxidation of lipids in patients with BD has also been the subject of various studies. Quantitative analysis confirmed that increased levels of thiobarbituric acid reactive substances (TBARS), formed as a byproduct of lipid peroxidation have been shown to be elevated. In addition, elevated levels of malondialdehyde (MDA), 4-hydroxynonenal (HNE), and 8-iso-PGF2α have been found in the prefrontal cortex (de Souza et al. 2014; Siwek et al. 2013).

Reactive nitrogen species in patients with bipolar disorder have also been the subject of studies. Increased levels of nitric oxide and nitrite in plasma have been observed and the amount correlated well with the severity of the disease (Aydemir et al. 2014).

As it has been proved by MRI, nitric oxide has the ability to enhance the vesicular release of glutamate. In addition, studies have also revealed significantly increased plasma amounts of 3-NT (3 neurotrophic factor) in patients with bipolar disorder. 3-NT is known to induce inflammation and may contribute to increased oxidative stress (Andreazza et al. 2009).

Bipolar disorder is also characterized by mutations to mtDNA and mitochondrial dysfunction caused predominantly by excess ROS (Andreazza et al. 2018; Yager et al. 2010). Analysis of post-mortem tissues showed an impaired function of complex I of the MRC (mitochondrial respiratory chain) (Kuloglu et al. 2002).

#### Antioxidant therapy and bipolar disorder

Coenzyme Q10 (ubiquinone, see also above) is a substance produced naturally by the body and has been found to be effective in alleviating symptoms of bipolar disorder. In two studies, 29 patients suffering from geriatric bipolar disorder were taking a lower dose of 200 mg of coenzyme Q every day (Aiken 2018). The nutrient improved vitality and energy of patients.

Omega-3 fatty acids have been studied for their neuroprotective properties, namely α-linolenic, eicosapentaenoic, and docosahexaenoic acids. These acids have been found to improve bipolar symptoms (Gabriel et al. 2022).

In a recent study, it has been reported that patients with bipolar disorder have low levels of vitamin B9 (folate) (Hsieh et al. 2019). 3 mg of folate supplements were effective (along with sodium valproate) in the treatment of the acute phase of bipolar disorder. Vitamin B9 supplements together with the traditional treatment of bipolar disorder appear to be more effective than the placebo effect (Zheng et al. 2020).

Some studies reported a minimum effect of N-acetyl Cysteine supplementation. However, a review paper discussing six clinical trials reported that N-acetyl Cysteine is superior to placebo, although with a mild effect, but a large interval of confidence (Nery et al. 2021). In another trial, N-acetyl Cysteine was used twice a day (1 g each time) for 24 weeks. It has been reported that N-acetyl Cysteine significantly improved (based on the Montgomery Asberg Depression Rating Scale, MADRS) state of patients with bipolar disorder (Berk et al. 2008). An open-label trial revealed a solid suppression of Bipolar Depression Rating Scale (BDRS) scores following 2 months of treatment with N-Acetyl-Cysteine in patients with moderate depression (Berk et al. 2011). In future studies, researchers should optimize the dose of N-Acetyl Cysteine, length of the supplementation, chronicity of illness, and other factors.

Vitamin D is an important element for calcium absorption, healthy bones, and a properly working immune system. It has been found that deficiency of vitamin D is nearly 5 times more common in patients with bipolar disorder than in the healthy population (Gowda et al. 2015). Surprisingly, supplementation of patients with vitamin D did not improve the symptoms of bipolar disorder patients with low levels of vitamin D. Many participants in the study were after treatment still deficient in vitamin D. From this follows that vitamin D deficiency should be treated.

200 mg of Coenzyme Q10 every day for a period of 8 weeks, together with medical treatment explored some benefits in patients with bipolar depression (Mehrpooya et al. 2018). The beneficial effect of coenzyme Q10 has been assigned to its antioxidant and inflammatory properties.

About 80% of patients with bipolar disorder in remission have problems sleeping (Geoffroy et al. 2017). Although melatonin does not target directly bipolar symptoms, improvement in sleep is an important factor in preventing mood disturbances in patients with bipolar disorder.

Several studies investigated the potential effect of a six-carbon sugar, inositol. No significant improvements in depression scores between control and bipolar groups were found (Eden Evins et al. 2006).

A 4 months double-blind study to investigate mood stabilizing effectivity of omega-3 fatty acids (cca 10 g a day) versus olive oil (placebo) in patients with bipolar disorder has been conducted. The results have shown that patients taking omega-3 fatty acids have much longer periods of remission than the untreated group (Stoll et al. 1999).

Ethyl eicosapentaenoic acid (E-EPA or icosapent ethyl) is used to treat depression. A double-blind study of patients with bipolar depression taking 1 g or 2 g of ethyl eicosapentaenoic a day for a period of 3 months has shown significant improvements compare to placebo (Frangou et al. 2006). In another study with even 6 g of ethyl eicosapentaenoic acid a day in the treatment of bipolar disorder both depressive symptoms and manic symptoms were evaluated (Keck et al. 2006). No significant differences between the ethyl eicosapentaenoic acid and placebo groups have been found.

### Diabetes

The Greek physician Aretaeus, a pupil of Hippocrates used in the 1st Century anno domini for the first time, the term “diabetes”. The name of this condition arose from the sweet smell of urine of individuals with elevated glucose levels. Diabetes mellitus is responsible for nearly 7 million deaths in 2021 worldwide (Gambale et al. 2022).

There are two main types, Type 1 and Type 2 diabetes. Type 1 diabetes is a chronic autoimmune condition, where the immune system destroys the pancreatic beta cells producing insulin. Type 2 diabetes is a condition, characterized by high blood glucose and inadequate response to insulin, called insulin resistance (Gambale et al. 2022). The third type of diabetes is known to develop in pregnant women who have not been diagnosed previously as diabetic. Although this type of diabetes usually disappears after a certain time, mother and child have a higher predisposition for Type 2 diabetes later in their life (American Diabetes Association 2022). Diabetes 2 type is the most common form of diabetes affecting more than 90% of people with diabetes (Polonsky 2012).

Oxidative stress has been implicated in the pathophysiology of diabetes (Black 2022). The major source of ROS-induced oxidative stress in diabetes mellitus is mitochondria. Under normal conditions, about 1–2% of total consumed oxygen is converted to superoxide radical anion and hydrogen peroxide, however, under hyperglycemic conditions, the conversion of oxygen to superoxide radical increases up to 10%. In addition to increasing levels of superoxide radicals, oxidative stress in diabetes is caused by the alterations in concentration of enzymes such as catalase, SOD, and glutathione peroxidase (GSH-Px).

It appears that the redox imbalance is an important etiologic contributor to insulin resistance, beta-cell dysfunction, and impaired glucose tolerance, all resulting in the development of type 2 diabetes (Wright et al. 2006). A significant, five–ten-fold increase in ROS-induced oxidative stress is also due to the consumption of a large amount of fatty food, unhealthy lifestyle with low physical activity, and escape from physiological chain regulation (Turrens et al. 1982).

Patients with type 2 diabetes, following postprandial hyperglycemia (rapid increase in blood levels of glucose) exhibit significantly increased oxidative stress markers (Ceriello et al. 2002).

**Fig. 23:**
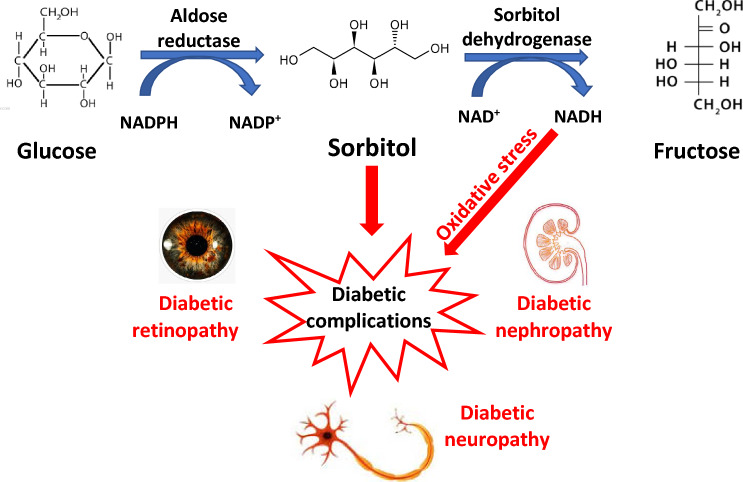
Polyol (Sorbitol pathway) it is estimated that about one-third of glucose in the body is metabolized by this pathway

Persistent hyperglycemia results in the activation of the Polyol (Sorbitol) pathway (see Fig. 23). The main function of this pathway is to convert glucose to fructose (e.g. for spermatocytes). The mechanism of this pathway requires a high concentration of glucose which is then converted to sorbitol by utilizing the enzyme Aldose reductase requiring coenzyme NADPH oxidized to NADP^+^ (Yan 2018). In the next step, sorbitol is converted to fructose by the enzyme Sorbitol dehydrogenase by utilizing NAD^+^ reduced to NADH. This Polyol (Sorbitol) pathway is activated only if the concentration of glucose in cells is high because the enzyme Aldose reductase has a relatively low affinity for glucose (and conversely high Michaelis Menten constant *K*_m_). If not converted to fructose, cellular accumulation of sorbitol may have various adverse health effects. The harmful effect of sorbitol is connected mainly with its high osmotic pressure and ability to absorb fluids in tissues. This problem is of particular importance in tissues with lower activity or concentration of Sorbitol dehydrogenase enzyme. The retina and lens of the eye have very low Sorbitol dehydrogenase activity and therefore accumulation of sorbitol may lead to diabetic retinopathy. Another organ with low activity of Sorbitol dehydrogenase is the kidney, resulting in the occurrence of Diabetic Nephropathy. Nerve cells have also low activity of this enzyme resulting in Diabetic Neuropathy.

#### Mitochondrial ROS sources in diabetes

As outlined above, the majority of molecular oxygen is processed by mitochondria involving four-electron reduction of dioxygen to water molecules. This is a finely tuned process consisting of several steps in which electrons are transferred from NADH and FADH2 donors through Complexes I-IV of the electron chain and protons into intermembrane space. The coenzymes NAD/NADH (NADH is Nicotinamide adenine dinucleotide) is an energy-rich coenzyme participating in the electron transport chain and Flavin adenine dinucleotide (FADH2) is a redox cofactor and utilized in the last part of respiration. These coenzymes can exist in oxidized (NAD^+^ and FAD) or reduced forms (NADH and FADH2). Under normal conditions, about 1–4% of oxygen is not reduced completely and is transferred to ROS, primarily to superoxide radical anion by one-electron reduction of oxygen (Iacobini et al. 2021; Houstis et al. 2006).

Hyperglycemia is associated with excessive ROS formation in mitochondria, as documented by increased DNA strand breaks. In response to this is activated *Poly (ADP-ribose) polymerase* (*PARP*), a family of proteins involved in various cellular processes such as DNA repair, genomic stability, and other processes. Activated PARP modifies glycolytic glyceraldehyde 3-phosphate dehydrogenase (GAPDH), an enzyme that catalyzes the process of glycolysis.

These results indicate that superoxide formation in mitochondria is necessary for the initiation of hyperglycemia-induced oxidative stress and oxidative damage (Du et al. 2003). In addition, several other sites of ROS production have been identified in mammalian mitochondria, however, their direct association with hyperglycemia is still under investigation (Wong et al. 2017). It has been reported, that in addition to mitochondrial sites of ROS formation, several non-mitochondrial sites may be triggered following hyperglycemia. These are for example reactions between reducing sugars and NADPH and oxidases uncoupled endothelial nitric oxide synthase.

Increased mitochondrial ROS formation has been associated with reduced mammalian target of rapamycin (mTOR). Chronic hyperglycemia results in the suppressed activity of mTOR associated with AMP-activated protein kinase-induced increase in ROS formation and enhanced expression of thioredoxin interacting protein (TXNIP) a potential sensor of glucose and oxidative stress (Akpoveso et al. 2023). Increased ROS formation is associated with a suppressed activation of protein kinase B AKT/mTOR resulting in apoptosis of β-cell (Chau et al. 2017). In fact, AKT/mTOR pathway plays a role in insulin signaling, and activation of this pathway is associated with enhanced survival of cardiomyocytes.

#### Non-mitochondrial ROS sources in diabetes

Regardless of whether excessive ROS formation is the primary initiator or accompanying factor in the pathogenesis of diabetes mellitus, oxidative stress plays a key role in the organ complications such as cardiovascular problems, neurological problems (stroke), diabetic retinopathy, nephropathy, and neuropathy; diabetic nephropathy being the most common, affecting 28% of the diabetic population (Jha et al. 2016; Kleinschnitz et al. 2010).

The other ROS-increasing conditions, such as inflammation, dyslipidemia, and upregulation of the renin-angiotensin system (blood pressure regulation by the hormone system) all contribute to increased markers of oxidative stress. Other ROS-generating pathways than mitochondria contributing to the pathogenesis of diabetes involve xanthine oxidase, endoplasmic reticulum stress, cytosolic ROS-producing enzyme NADPH oxidase (NOX), uncoupled Nitric oxide synthase (NOS), and myeloperoxidase (Iacobini et al. 2021).

The NOX enzymes together with mitochondrial superoxide represent the most important sources of ROS in diabetes mellitus and diabetic micro-/macro-vascular diseases (Forbes et al. 2008; Miric et al. 2016).

Whereas various enzymes produce ROS as by-products of their normal physiological activity, NOXs are the only enzyme family able to generate ROS as its primary function (Casas et al. 2015). The specific role of NOXs in diabetes is more complex than just increasing ROS production. The cardiovascular risk associated with hyperglycemia in diabetes is explained by the atherogenic process substantiated by the increased ROS formation. For example, NOX-5-derived superoxide radical formation is associated with cardiovascular diseases in humans (Guzik et al. 2008). It has been reported that NOX5-derived superoxide radical promotes diabetic nephropathy via increased renal hypertrophy, kidney fibrosis, and albuminuria (Jha et al. 2014). In contrast with this study, NOX4-derived hydrogen peroxide exhibited anti-atherosclerotic effects by smooth muscle cell regulation in diabetic mice (Di Marco et al. 2016; Cortinovis et al. 2022). NOX-4 has been found to be involved in inflammation in diabetes and its deletion resulted in the downregulation of Nuclear factor-kappa B (NF-κB) signaling, an important mediator of inflammation (Jha et al. 2016).

#### Antioxidant therapy in diabetes

Effective antioxidant therapy should protect the beta-cells against ROS-induced apoptosis and preserve their normal functioning. Antioxidants may to some extent alleviate diabetic-associated complications and restore insulin sensitivity.

Streptozotocin is a naturally occurring agent toxic to pancreatic beta cells. The role of vitamin E supplementation (intraperitoneally 500 mg/kg for 8 days) on peroxidation of lipids and overall antioxidant status (GSH, SOD, GPx) in blood and liver tissues in streptozotocin-induced diabetic rats has been studied. Streptozotocin is a naturally occurring agent toxic to pancreatic beta cells (Seven et al. 2004). Vitamin E has been shown to decrease the hepatic peroxide level in diabetic rats. Supplementation with E is known to improve the level of glucose under diabetic conditions. Orally administrated diabetic (intraperitoneal injection of streptozotocin 60 mg/kg) and control Wistar rats were supplemented with vitamin E (0.2, 0.4, 0.8 mg/kg for 3 weeks). It has been shown that vitamin E significantly reduced glucose levels in the blood compared to untreated rats. The beneficial effect of vitamin E on the hyperglycemic diabetic rats was found to be dose-dependent. In addition, the oral glucose tolerance test was improved following vitamin E supplementation of diabetic rats, thus vitamin E appears to play a role in the metabolism of glucose (Al Shamsi et al. 2004).

In another rat experiment, vitamin E has been shown to control hyperglycemia and lower glycated hemoglobin, and HbA1c, by suppressing the oxidative stress level (Ihara et al. 2000).

In another study patients suffering from diabetes 1 (less than 10 years) and healthy subjects were supplied with 1800 IU of vitamin E per day or placebo for 4 months (Bursell et al. 1999). The results have shown that the serum level of vitamin E was significantly increased in both type 1 diabetic and control subjects. Glycated hemoglobin A1c (HbA1c) was improved by the vitamin E supply. Retina blood flow was evaluated using fluorescein angiography and was significantly lower compared to that of non-diabetic subjects. After treatment with vitamin E retina blood flow significantly increased in the group of diabetic patients and reached the level of non-diabetic subjects. In addition, vitamin E treatment significantly normalized elevated creatinine clearance in patients with diabetes 1. It appears that vitamin E is effective in restoring retinal hemodynamic properties in type 1 diabetic patients without changes in glycemic control. The overall benefit of diabetes 1 patient taking vitamin E is reduced risk of diabetic retinopathy and nephropathy development.

The mechanism of normalizing retina blood flow by vitamin E was evaluated using animal studies and involves the diacylglycerol (DAG) activating protein kinase C (PKC) through binding to isozyme C1 domains (Koya et al. 1997; Bursell et al. 1997).

In another study, the effect of vitamin C and magnesium supplementation was evaluated in 56 diabetic outpatients (Eriksson and Kohvakka 1995). During 90 days treatment period were patients supplied with 600 mg/day of magnesium and 2 g/day of vitamin C. A slight decrease in blood pressure (both systolic and diastolic) was observed in insulin-dependent diabetic subjects taking magnesium. No improvements in blood pressure and glycemia have been observed in non-insulin-dependent diabetic subjects. Supplementation with vitamin C improved glycemic control in non-insulin-dependent diabetic patients as well as glycated hemoglobin (HA1c) and fasting blood glucose. In addition, supplementation with vitamin C improved the level of cholesterol and triglycerides showing the beneficial effect of vitamin C on glycemic control and lipids in non-insulin-dependent diabetic subjects.

The association between the consumption of vegetables and fruits and glucose tolerance was studied in 122 subjects (40–64 years of age) (Williams et al. 1999). After adjustment for age, gender, and family history it has been found that frequent consumption of fruit and vegetables during all year has been inversely correlated with the risk of developing non-insulin dependent diabetes mellitus. Consumption of vegetables only in the summer months had a significantly weaker inverse correlation with the risk of developing non-insulin-dependent diabetes mellitus.

Alpha lipoic acid (ALA) or thioctic acid is an antioxidant soluble in water and fat in the body and is readily absorbed in the gastrointestinal tract. Alpha lipoic acid has been shown to increase the utilization of glucose in various experimental models after acute and chronic administration (Jacob et al. 1996). Twenty patients with type II diabetes (non-insulin-dependent diabetes mellitus) received 500 mg of alpha lipoic acid in 500 ml 0.9% NaCl during the 10 days in the form of infusions. Administration of alpha lipoic acid resulted in a marked increase (about 30%) in insulin-mediated glucose disposal. No changes in fasting plasma levels of glucose or insulin have been observed. This was the first clinical trial showing that 10 days of administration of alpha lipoic acid is able to manage insulin-stimulated glucose disposal in non-insulin-dependent diabetes mellitus.

Packer et al. reported that alpha-lipoic acid is capable of scavenging ROS formed in the course of lipid peroxidation and preventing diabetic complications such as cataract formation, polyneuropathy, and vascular damage (Packer et al. 2001).

Experimental evidence supports selenate as an efficient insulin-mimetic (Stapleton 2000). For example, selenate has been shown to reduce the glucose level in streptozotocin-induced diabetes in laboratory animals. In addition, selenate has been shown to regulate fatty acid and glycogen synthesis. Selenium is also involved in the insulin signal transduction pathway affecting the expression of important metabolic enzymes.

Based on the discussed results, antioxidants such as vitamins E and C, lipoic acid, and selenium have hypoglycemic effects, however, supplementation of patients with these vitamins did not bring the desired effect. Although the intake of antioxidants minimizes complications related to diabetes, the mechanism behind the antioxidant action requires further studies (Rajendiran et al. 2018).

### Renal diseases

About 2–3 million people worldwide suffer from chronic kidney diseases (World Kidney Day 2015; Woo et al. 2012). Various cardio-related diagnoses, such as diabetes, hypertension, and dyslipidemia have been reported in patients suffering from chronic kidney diseases (Sarnak et al. 2003).

The kidney and the heart are one of the most energy-demanding organs in humans and this makes these organs vulnerable to ROS-induced damage (Sureshbabu et al. 2015). In fact, oxidative stress is a common denominator of kidney diseases and associated complications such as cardiovascular diseases and inflammation. The uncoupling of endothelial nitric oxide synthase (eNOS), oxidative stress mechanism is involved in uremia, a condition when the kidney does not work properly and waste products instead of urine are excreted into the bloodstream (Himmelfarb et al. 2002).

Oxidative stress in chronic kidney diseases is characterized by several distinct pathways: oxidative stress mediated by ROS (^·^OH), nitrosative stress mediated by RNS (ONOO^−^, ^·^NO_2_), chlorinated stress mediated by chlorinated species (HOCl), and carbonyl stress (Advanced glycation end products, AGEs) (Ling and Kuo 2018).

Direct involvement of oxidative stress in the pathophysiology of kidney diseases involves impaired renal function (Himmelfarb 2005). Since the physiological level of ROS plays an important role in normal kidney functioning, any changes in redox equilibrium in the renal cortex and medulla may have serious consequences. These involve changes in renal blood flow over renal blood flow over Na^+^ retention, inflammation, fibrotic disorders, and high level of proteins in urine (proteinuria) (Nistala et al. 2008). Deteriorated renal function correlates well with the altered markers of oxidative stress. These involve mitochondrial oxidized low-density lipoprotein (Garcia-Bello et al. 2014), homocysteine (Chien et al. 2015), and deficiency of reduced glutathione (GSH) and superoxide dismutase (SOD) (Garcia-Bello et al. 2014).

Toxins in urine have been found to increase oxidative stress markers and worsen renal functions such as retention solute, increased F2-isoprostanes, malondialdehyde, and asymmetric dimethylarginine (Tyagi et al. 2005).

The causative role of oxidative stress in kidney diseases has been confirmed in kidney damage and associated complications caused by type 1 and type 2 diabetes (diabetic nephropathy). Oxidative stress has been increased as a result of mitochondrial dysfunction, endothelial nitric oxide synthase (eNOS) uncoupling, resulting in endothelial dysfunction, the aberrant activity of NADPH oxidases (NOX), suppressed activity of antioxidant enzymes and low molecular weight antioxidants (Galvan et al. 2017).

Mitochondrial dysfunction has been documented by functional changes in the kidney mitochondria (Galvan et al. 2017). Altered functioning in kidney mitochondria has been reported also in non-diabetic patients with chronic kidney diseases (Granata et al. 2009) more particularly autosomal dominant polycystic kidney disease (ADPKD), the most common polycystic kidney disease and very common genetic kidney disease characterized by the formation of cysts in the kidneys (Ishimoto et al. 2017).

NADPH oxidases (NOX) are activated by different mechanisms. NOX4 is known to be activated by the indoxyl sulfate (IS), an aggressive uremic toxin which in turn results in increased ROS formation. Longstanding Angiotensin II receptor blockade (ATII-R, e.g. Azilsartan, Losartan) relaxes veins and arteries and lowers blood pressure, which in turn may prevent/treat/improve symptoms of patients who have kidney failure due to diabetes or chronic kidney diseases. This is at least partly due to suppressed oxidative stress as a result of decreased NOX expression and improved eNOS and SOD expression (Finch et al. 2011).

Zinc has anti-inflammatory and immune system-stimulating properties. Several studies reported the critical role of zinc in oxidative stress-related kidney diseases. For example, NOX-mediated ROS formation in the kidney has been associated with zinc deficiency (Li et al. 2017).

As already discussed, eNOS uncoupling, due to the various uremic toxins, including asymmetric dimethyl-arginine, is a ROS contributor to oxidative stress and ROS-derived damage in the kidney (Ueda et al. 2007).

Development of kidney disease is associated with the enzyme myeloperoxidase (MPO), abundant in neutrophils catalyzing the transformation of hydrogen peroxide (H_2_O_2_) to hypochlorous acid (HOCl), a powerful oxidizing cytotoxic agent, according to the reaction

32 $${\mathrm{H}}_{2}{\mathrm{O}}_{2} +\mathrm{ C}{\mathrm{l}}^{-}+{\mathrm{ H}}^{+} \to \mathrm{ HOCl }+ {\mathrm{H}}_{2}\mathrm{O}$$

HOCl has been found to be responsible for renal dysfunction among obese children. These children have shown a positive correlation between an isoprostane 8-iso-prostaglandin F2α (8-iso-PGF2α) and blood lipids, serum levels of MPO, and insulin resistance (Yu et al. 2013; Correia-Costa et al. 2016).

Patients with chronic kidney diseases exhibit increased activity of xanthine oxidase (XO). Hyperuricemia, a condition of abnormally high levels of uric acid in blood has been proposed to be associated with the abnormal levels of xanthine oxidase and chronic kidney diseases. Treatment using XO inhibitors showed a mild protective effect on renal function in patients with hypertension (Kohagura et al. 2016).

The main detoxification enzyme, SOD, is highly expressed in kidneys. Impaired functions of SOD have been reported in patients with chronic kidney diseases (Tbahriti et al. 2013). In addition to SOD, impaired activity of glutathione synthesis in patients with renal failure has been reported (Girelli et al. 1993).

Dialysis patients exhibit deficiency in blood selenium which has been partly attributed to dietary restrictions, however, the exact mechanism is not known (Zachara 2015).

Heme oxygenase-1 (HO-1) is a critical cytoprotective enzyme catalyzing the breakdown of heme to biliverdin and iron producing carbon monoxide (CO) as a byproduct. The role of HO-1 in kidney diseases has been studied very scarcely. Shorter HO-1 (GT)n alleles have been found to improve acute kidney injury and outcome after kidney transplantation in mouse models (Leaf et al. 2016), at least partly due to the increase in HO-1 promoter activity (Gill et al. 2018).

In conclusion, various endogenous and exogenous pathophysiological mechanisms in patients with chronic kidney diseases lead to enhanced activity of oxidative enzymes such as xanthine oxidase (XO), myeloperoxidase (MPO), and the NADPH oxidase enzymes (NOX). Abnormally regulated homeostasis of enzymes results in mitochondrial dysfunction, eNOS uncoupling, formation of secondary ROS, and disturbed metabolism of redox and non-redox active metals.

#### Antioxidant therapy in renal diseases

Carotenoids, flavonoids, alkaloids, and other natural products have been shown to be effective as preventive and therapeutic agents against oxidative mechanisms occurring in kidney diseases (Kanlaya and Thongboonkerd 2019).

The antioxidant and reno-protective mechanisms have been observed for the carotenoid molecule, crocin, by declining Interleukin-18 (IL-18), Nox-4, and p53 and improving diabetic nephropathy (Yaribeygi et al. 2018).

Flavonoid quercetin alleviates oxidative stress in diabetic nephropathy (Tong et al. 2017), natural alkaloid berberine can inhibit the progression of the disease [Wu et al. 2012), and naturally occurring cinnamaldehyde is able to suppress oxidative stress in kidney diseases (Zheng et al. 2011).

The Ginger (Zingiber officinale) can prevent diabetes-induced nephropathy through AMP-activated protein kinase (AMPK) (Tzeng et al. 2013).

The use of gold nanoparticles as drug carriers is an efficient way for the treatment of kidney diseases. For example, an extract from Ficus carica, a plant native to the Mediterranean region transported by gold nanoparticles has been shown to reduce toxicity caused by cis-platin-induced oxidative stress (El-Sayed et al. 2019).

The kidney is highly abundant in mitochondria and therefore represents a vulnerable site to ROS-induced damage. Supplementation with antioxidants in both chronic and acute stages of kidney diseases may have therapeutic potential.

Nicotinamide adenine dinucleotide phosphate oxidases (NOXs) are ROS sources coupled to the electron transfer process across biological membranes. NOXs have been found to be disturbed in diabetic nephropathy, ischemia, kidney tumors, hypoxia, and other conditions (Tsuchiya et al. 2018).

Various in vivo studies evaluated the antioxidant efficiency of naturally occurring antioxidants against acute and chronic kidney diseases. Most of the drugs obtained from natural products, such as allicin, an oily liquid occurring in garlic, hesperidin, a citrus flavonoid, resveratrol, a stilbenoid polyphenol found in grapes, betanin, a powerful antioxidant, 6-gingerol, isolated from ginger rhizomes, and α-bisabolol, an active ingredient of chamomile have been studied.

Allopurinol is a XO inhibitor and one of the most effective drugs used to suppress urate levels. N-acetyl-cysteine and N-acetylglucosamine are efficient antioxidants. All these three substances have been tested in animal models showing protective effects on kidneys and intestine and heart. N-acetyl cysteine was able to prevent acute kidney injury (acute renal failure) as documented by reduced biomarkers of oxidative stress and inflammation and increased antioxidant biomarkers (Ozturk et al. 2018).

Nrf2 (nuclear factor erythroid 2–related factor 2) is an important regulator of antioxidant defense against toxic substances. In a recent animal study, it has been confirmed that melatonin was able to attenuate kidney ischemia/reperfusion by the triggering SIRT1/Nrf2/HO-1 signaling pathway (Shi et al. 2019).

α-Lipoic acid is both water and fat-soluble and acts as a regenerative antioxidant in living systems. α-lipoic acid has been shown to have a protective anti-inflammatory effect in patients undergoing simultaneous kidney and pancreatic transplantation (Ambrosi et al. 2016). The preventive evaluation revealed that the serum inflammatory markers such as IL-8 and IL-6 were markedly reduced.

Hemodialysis therapy in patients is accompanied by increased markers of oxidative stress which in turn leads to increased hospitalization and mortality. 353 patients undergoing hemodialysis were receiving over 6 months of antioxidant therapy (600 mg/day of alpha-lipoic acid and 666 IU/day of mixed tocopherols). While treatment was well tolerated it did not improve plasma levels of oxidative stress markers such as F2 isoprostane, isofuran, and IL-6 (Himmelfarb et al. 2014).

Bardoxolone methyl (BARD) is an anti-inflammatory naturally occurring drug obtained from oleanolic acid. Bardoxolone is known to activate the Nrf2-Keap1 pathway, which in turn inhibits the proinflammatory cytokine NF-κB and maintains normal kidney function (Pergola et al. 2011). A double-blind, randomized, placebo-controlled trial employing 227 adults with chronic kidney diseases were taking 25 mg, 75 mg, or 150 mg of bardoxolone methyl every day for a period of 24 weeks or 52 weeks. Patients receiving bardoxolone methyl had significantly increased glomerular filtration rate, a test showing how well the kidneys are filtering. The most common side effects of bardoxolone methyl, muscle spasms were mild and predominantly dose-dependent. Thus, bardoxolone methyl is a promising substance for the treatment of chronic kidney diseases.

Coenzyme Q (ubiquinone) has been investigated in preventing renal injury in patients with renal lithiasis, a disease caused by calculi or stones in the kidneys or urinary tract (Carrasco et al. 2014). A significant improvement in glomerular filtration and a decrease in the albumin/creatinine ratio and β2-microglobulin levels have been observed in the group taking coenzyme Q10 for 1 week. Thus, the administration of coenzyme Q10 improves kidney function and inflammation parameters.

Curcumin has various beneficial pharmacological properties (Samadian et al. 2017). Several clinical trials of various models of chronic kidney diseases have been carried out using curcumin which resulted in the suppression of inflammation parameters at the level of IL-6 and TNF-α (Samadian et al. 2017). In addition, curcumin has been found to alleviate lipid peroxidation in subjects with nondiabetic chronic kidney disease proteinuria, characterized by the increased amount of proteins in urine. An increased antioxidant capacity manifested as enhanced antioxidant action on Nrf2 activation has been observed.

Gentamicin is an aminoglycoside antibiotic used to treat various bacterial infections. However, gentamicin is known to behave as a prooxidant enhancing the formation of hydroxyl radicals, superoxide anion radicals, and hydrogen peroxide (Galaly et al. 2014). Curcumin showed a protective antioxidant effect against gentamycin-induced oxidative stress documented by alleviated lipid peroxides, increased levels of superoxide dismutase and glutathione, increased expression of Bcl-2, a protein protecting against oxidative stress, suppressed proapoptotic Bax, and suppressed executioner caspase in apoptosis, caspase-3. In addition to the antioxidant effect, all these mechanisms have also renoprotective effects.

Although selenium is not involved in the direct scavenging of ROS/RNS, its basic role as a cofactor of glutathione peroxidase is peroxide (hydrogen peroxide) reduction (Huang et al. 2005). In a clinical trial, 291 patients requiring elective off-pump coronary bypass graft surgery were receiving N-acetylcysteine (600 mg), selenium (0.5 mg), and vitamin C (1500 mg) orally twice a day (Amini et al. 2018). This group of patients was examined for the development of acute kidney injury. Of 272 patients who completed the study, acute kidney injury was observed in 60 patients (22%). The results have shown that perioperative administration of N-acetylcysteine, selenium, and vitamin C is not effective in preventing acute kidney injury following bypass surgery.

### Lung diseases

Lung diseases are one of the most common conditions worldwide. The majority of lung diseases are caused by smoking, air pollution, infections, and genetic disorders. Lungs are part of a very complex system and lung diseases arise when any part of this system experience problems. Lung diseases affect the airways, alveoli, interstitium, blood vessels pleura, and chest wall (Zablocka-Slowinska et al. 2019). Most common lung diseases involve asthma, chronic obstructive pulmonary diseases (COPD), bronchitis, pneumonia, tuberculosis, cancer, acute respiratory distress syndrome in COVID-19, pneumothorax, and other conditions. In this subchapter, we will discuss COPD and lung cancer conditions and their connection with oxidative stress (Domej et al. 2014).

### Chronic obstructive pulmonary disease (COPD)

Chronic obstructive pulmonary disease affects approximately one-tenth of people older than 40 years of age worldwide. COPD is currently the third most common cause of death after cardiovascular diseases and stroke (GBD Chronic Respiratory Disease Collaborators 2020).

In high-income countries is cigarette smoking a main risk factor for COPD. In addition, outdoor pollution is another important risk factor for COPD, especially exposure to particulates, oxides of nitrogen (NO_x_), and ozone (O_3_) predominantly in large Asian cities (Adamkiewicz et al. 2020). Occupational exposure is another important risk factor, most importantly among firemen and farmers.

Oxidative stress appears to be a key factor in the pathophysiology of COPD via several distinct mechanisms (Kirkham and Barnes 2013; McGuinness and Sapey 2017). COPD is tightly linked with chronic inflammation which affects peripheral airways and pulmonary parenchyma. Enhanced inflammation is accompanied by an increased abundance of alveolar macrophages, neutrophils, and T lymphocytes. These cells secrete various proinflammatory mediators such as cytokines, chemokines, growth factors, and lipid mediators. The majority of patients with COPD have neutrophilic inflammation. Enhanced oxidative stress is known to enhance COPD-related inflammation in ex-smokers which in turn may activate proinflammatory transcription factors involving nuclear factor kappa B (NF-κB), DNA damage, cellular senescence, and formation of autoantibody. Systemic inflammation is found in COPD patients and negatively affects co-occurring disorders such as diabetes, cardiovascular diseases, and osteoporosis (Barnes 2016).

A well-known biomarker of oxidative DNA damage, 8-hydroxy-Gua, is increased in the peripheral lungs of patients with COPD, confirming thus the occurrence of oxidative stress in the lungs (Caramori et al. 2011). Contrary to normal smokers, COPD patients have defects in DNA repair, as documented by very low expression of the double-stranded repair protein Ku86 (Caramori et al. 2011). The impaired DNA repair and resulting oxidative stress in patients suffering from COPD explain a higher prevalence of lung cancer compared to smokers without airway obstruction (Adcock et al. 2011).

Autoimmunity, a failure of the body’s immune system plays a role in promoting COPD. Various autoantibodies against epithelial cells and against collagen have been reported in COPD patients which in turn increases disorder severity (Byrne et al. 2019).

### Lung cancer

Lung cancer is considered one of the most common cancers and the leading cause of cancer-related deaths (Bray et al. 2018). Lung cancer accounts for about 20% of all cancer deaths. From a histological point of view, there are two types of cancers, small-cell lung cancer (incidence of cca 15%) and non-small cell lung cancer (incidence of cca 85%), subdivided into adenocarcinoma, squamous cell carcinoma, and large-cell carcinoma (Inamura 2017). About 40% of lung cancers are adenocarcinomas, which form from mucous-producing cells, capable of spreading beyond lung tissue in about 20% of cases before they are diagnosed (Bray et al. 2018). The gas phase of tobacco smoke contains approximately 10^15^ ROS/inhalation. About 85% of lung cancers are associated with smoking, however, lung cancer occurs also among non-smokers and represents the seventh most common cause of cancer mortality which indicates that other factors such as genetic predisposition, diet, silica, asbestos, high energy radiation, concentration of radon and other factors play a role.

#### ROS, oxidative damage, inflammation, and lung cancer

Tobacco smoke contains more than 4500 chemical compounds which are present in both tar and gas phases. The most dangerous carcinogens in tar are the polycyclic aromatic hydrocarbons such as nitrosamine ketones derived from nicotine and benzopyrenes, organic compound butadiene and redox carcinogenic metal chromium, non-redox cadmium, and arsenic, and radioactive polonium (Sun et al. 2007). The tar phase contains very high radical concentration (cca 10^17^ radicals/gram). These radicals are relatively stable. Gas phase tobacco contains cca 10^15^ radicals, mostly shortly lived carbon and oxygen-based with a lifetime < 1 s. (Mousapasandi et al. 2021). The most abundant ROS in tar are semiquinone radicals (SQ^·−^) which react with oxygen forming superoxide radical

33 $${\text{SQ}}^{{ \cdot - }} + {\text{ O}}_{2} \to {\text{ SQ }} + {\text{ O}}_{2}^{{ \cdot - }}$$

which is further able to dismutase to form hydrogen peroxide and consequently react with iron, present in the tar, by the Fenton reaction, resulting in the formation of hydroxyl radicals.

Silica can be inhaled during the grinding of industrial minerals, hard-rock mining, or sand-blasting. Inhaled silica reaches the alveolar space and depending on the silica surface small particles are either cleared from the lungs by macrophages or activated macrophages which in turn produce ROS and RNS, such as superoxide radical and nitric oxide, respectively. Formed ROS and RNS can either damage epithelial cells in the lungs or may react together to form ONOO^−^ (peroxynitrite) (see above).

Asbestos fibers are used in roofing, insulation, plumbing, and other processes and are inhaled during processing, mining, and extraction. Inhaled asbestos is responsible for the accumulation of macrophages in the alveolar space. The mechanism of ROS/RNS formation is similar to that described for silica (Guidotti et al. 2004). The asbestos fibers contain redox-active iron on their surface which catalyzes the decomposition of hydrogen peroxide by means of the Fenton reaction or Haber–Weiss reaction producing hydroxyl radicals.

Exposure to radioactive radon can be a very serious cause of lung cancer. The decay of radon produces alpha particles forming ROS such as superoxide radical and hydrogen peroxide which in turn induce oxidative cellular damage, including elevated levels of 8-OH-dGuanine in lungs and other organs.

Inhaled carcinogenic agents promote lung tumorigenesis through ROS formation and consequently ROS-induced oxidative damage. The following lung cells, alveolar macrophages, endothelial cells, smooth muscle cells, lung fibroblasts, inflammatory cells, and peripheral monocytes/macrophages contain enzymes, such as membrane-bound NADPH oxidases, capable of ROS formation. Carcinogenic species participating in ROS formation may trigger overexpression of the phosphorylated epidermal growth factor receptor (EGFR) protein and its ligand amphiregulin (AREG) a widely expressed transmembrane tyrosine kinase which in turn promotes uncontrolled proliferation of lung adenocarcinoma cells (Kometani et al. 2009).

Exogenous sources, involving tobacco smoke, asbestos, radon, and others increase ROS formation which consequently activates transcription factors NF-κB and activator protein AP-1. These transcription factors are responsible for the gene transcription of proinflammatory cytokines, such as tumor necrosis factor (TNF-α) and interleukins IL-8 and IL-6 which in turn sequestrate leukocytes such as neutrophils and macrophages forming thus a part of an inflammatory pathway (Drost et al. 2005; Federico et al. 2007).

Leukocytes produce ROS such as superoxide radicals, hydroxyl radicals, and peroxynitrite to inactivate pathogens. Cyclic formation of ROS promotes chronic inflammation resulting in DNA damage, mutations, and loss of repair and inhibition of apoptosis, all contributing to lung cancer incidence.

Nearly half of lung cancer patients have mutations in their mitochondrial DNA (mt DNA). These mutations can be detected from exhaled breath condensate of patients (Yang et al. 2013). mtDNA mutations cause alterations in the mitochondrial electron transport chain which promotes aerobic glycolysis, a hallmark property necessary for tumor growth and metastasis in lung cancer. Preservation of the mtDNA integrity may represent a suitable therapeutic approach (Ishikawa et al. 2008).

Another study reported that mitochondrial ROS are necessary for the KRAS (Kirsten rat sarcoma virus)—induced anchored-independent growth of lung cancer. The main source of ROS formation is the Q_o_ site of mitochondrial complex III. K-ras is a part of the RAS/MAPK pathway (Weinberg et al. 2010).

ROS from different sources causes DNA damage which in turn enhances genomic instability in lung cancer (Azad et al. 2008). DNA damage can be prevented by tumor suppressor protein p53 encoded by the TP53 gene which is the most mutated gene in human cancers, playing a key role in DNA damage response and is considered as “Guardian of the Genome”. The most frequently occurring mutations in the tumor suppressor gene TP53 in lung cancer are Guanine → Thymine and Guanine → Adenine transversions (Srinivas et al. 2019; Gibbons et al. 2014). If the damage escapes repair mechanism, permanent DNA mutations may lead to genomic instability which is a first step to metabolic instability and suppressed synthesis of antioxidant enzymes.

#### Antioxidant therapy in chronic obstructive pulmonary disease

Smoking and chronic obstructive pulmonary disease are directly related and result in depleted sulfhydryls and antioxidant capacity in plasma (Rahman 2006). Depletion of vitamins C and E, carotenoids, and selenium has been observed in the serum of smokers and patients with chronic obstructive pulmonary disease (Tug et al. 2004). To restore the antioxidant defense system, supplementation with antioxidants is one of the possibilities.

A positive correlation between dietary intake of vitamin E and pulmonary function has been observed in a group of 2633 subjects. Vitamin E has been shown to have a protective effect against the development of chronic obstructive pulmonary diseases. Dietary polyphenols may also be an efficient therapy to prevent or inhibit oxidative stress which is a key factor in the development of chronic pulmonary obstructive disease (Rahman 2006).

The administration of N-acetyl cysteine has been used with variable success to increase the level of glutathione in the lungs of smokers with chronic pulmonary obstructive diseases. Patients, receiving 600 mg of N-acetyl cysteine twice a day for 12 months have suppressed plasma and bronchoalveolar lavage fluid oxidative biomarkers (Van Schooten et al. 2002). Similarly, supplementation with N-acetyl cysteine 600 mg every day for a period of 12 months suppressed the concentration of hydrogen peroxide in exhaled breath condensate compared to the placebo group (Kasielski and Nowak 2001).

N-acystelyn is a lysine salt of N-acetylcysteine with antioxidant and mucolytic properties. Contrary to acidic N-acetyl cysteine, N-acystelyn has neutral pH. It has been reported that N-acystelyn inhibits hydrogen peroxide/superoxide radicals and increases intracellular glutathione in alveolar epithelial cells in smokers with chronic obstructive pulmonary disease (Gillissen et al. 1997). N-acystelyn inhibits oxidative stress-mediated Interleukin-8, a chemokine produced in alveolar epithelial A439 cells, confirming thus the anti-inflammatory effect of N-acystelyn.

A manganese-containing complex, GC4419, belongs to a macrocyclic Mn(II) class of SOD mimetic compounds with dismutation rates comparable to the native enzyme Mn-SOD (Tuder et al. 2003). Pretreatment of guinea pigs with Mn-based mimetic complex reduces both smoking-induced expression of IL-8 and activation of NF-κB (Nishikawa et al. 1999).

Carbocisteine (or carbocysteine, S-carboxymethyl cysteine) is an antioxidant and mucolytic cysteine donor used in Asia and Europe to treat patients suffering from the chronic pulmonary obstructive disease (Yasuda et al. 2006). Several observational studies confirmed that one year of treatment with carbocisteine resulted in a decreased number of respiratory exacerbations and significantly improved quality of life (Esposito et al. 2016).

Erdosteine is a mucolytic antioxidant containing two blocked -SH groups that are converted to three metabolites. A placebo-controlled trial containing 124 patients with chronic pulmonary obstructive disease received 300 mg of erdosteine twice a day for 8 months (Moretti et al. 2004). The results demonstrated improved quality of life and suppressed respiratory exacerbations. These trials suggest that intake of thiol or thiol-related mucolytic substances (cca. 300–600 mg twice a day) with radical scavenging/antioxidant properties reduces the incidence of respiratory exacerbations and improves the quality of life.

The nuclear factor erythroid 2-related factor 2 (Nrf2) is an important transcription factor of the antioxidant response via regulating gene expression with the antioxidant-responsive. Agonists of Nrf2 significantly enhance antioxidant synthesis (Vézina and Cantin 2018).

Several exogenous and endogenous Nrf2 agonists have been evaluated. Selenium supplementation enhances the synthesis of electrophilic cyclopentenone 15d-PGJ2, a very potent Nrf2 agonist, significantly increasing the cellular antioxidant pool and anti-inflammatory properties (Greene et al. 2013). Due to the relative instability of 15d-PGJ2, analog molecules with related properties have been prepared and tested in preclinical studies, involving sulforaphane, chalcones, and 2-cyano-3,12-dioxooleana-1,9-dien-28-oic acid (CDDO).

Sulforaphane is a potent isothiocyanate agonist of Nrf2 occurring in vegetables. Trial with patients suffering from chronic pulmonary obstructive disease failed to observe any changes in alveolar macrophage and expression of antioxidant genes (Wise et al. 2016).

Chalcones (1,2-diphenyl-2-propen-1-one) are synthesized by various plants and represent a part of a healthy diet. Chalcones have been shown to reduce lung inflammation, however, the effect on chronic pulmonary obstructive disease is not known (Lv et al. 2017).

CDDO has been shown to protect mice against cigarette smoke exposure through the Nrf2 mechanism (Sussan et al. 2009). However, clinical studies using CDDO have not been reported.

#### Antioxidants and lung cancer

The relationship between lung cancer and antioxidants is very peculiar. Dietary antioxidants either in the natural form or in the form of supplements have been for a long time considered as healthy substances able to prevent or improve/treat various disease states of an organism.

The first shocking results about the effect of antioxidants in the prevention of lung cancer were published in mid 90’s (Middha et al. 2019). A total of 29,000 male smokers, aged 50–69 years for the period of 5–8 years were assigned to one of the four groups, according to treatment, (i) α-tocopherol alone (50 mg/day), (ii) β-carotene alone (20 mg/day), (iii) α-tocopherol and β-carotene and (iv) placebo. The results of the trial have shown that smokers receiving β-carotene had a higher incidence of lung cancer regardless of the tar or nicotine content of the cigarette smoke.

It has been believed that cancer patients supplementing their diet with antioxidants may inhibit cancer progression. However, various clinical trials disproved this approach (Klein et al. 2011). Antioxidants have been found to interfere with signaling pathways and are able to overcome barriers against tumor progression. Antioxidant supplementation of the diet has been found to accelerate tumor progression in immunodeficient mice with implanted patients’ lung cancer or malignant melanoma cells (Piskounova et al. 2015).

In the work, of Sayin and his group, (Sayin et al. 2014) diet supplemented with vitamin E and N-acetylcysteine resulted in increased tumor progression and suppressed survival in mouse models of KRAS and BRAF mutations in lung cancer. KRAS gene makes a protein K-ras, a member of the RAS/MAPK pathway, and the BRAF gene makes a protein B-raf, involved in signals important for cell growth. Mutated B-raf has been found in various cancers (Davies et al. 2002).

Although vitamin E and N-acetylcysteine are structurally different molecules, interestingly, in cellular systems they act almost synergistically in tumor transcription profiles and enhance the proliferation of tumor cells accompanied by reduced expression of tumor suppressor protein p53 and other changes in human and mouse lung cancer cells. Quantitatively, the effect of p53 inactivation is comparable with the effect of antioxidants accelerating the growth of tumors by disturbing the ROS-p53 axis (Sayin et al. 2014).

In another work, long-term administration of antioxidants, vitamin E, and N-acetylcysteine has been shown to promote KRAS-mediated lung cancer metastasis (Wiel et al. 2019). The antioxidants were responsible for suppressing levels of free heme and stabilization of transcription factor BACH1, important in heme homeostasis, maintenance of cell cycle, ROS formation, and other processes. Subsequently, BACH1 activates transcription factors involved in the triggering process of glycolysis playing an important role in tumor growth promotion, metastasis, and chemoresistance in mouse and human lung cancer cells.

Complementary and in agreement with these results is work by Lignitto and his group (Lignitto et al. 2019). Lung cancer cells are characterized by increased ROS production and ROS-induced oxidative stress is maintained by enhanced transcription of antioxidant enzymes. It has been reported that in response to oxidative stress, Nrf2 accumulated and stabilizes a pro-metastatic transcription factor BACH1 by inducing HO-1. Thus inhibition of HO-1 appears to be a promising therapy to prevent lung cancer metastasis. This recent important work suggests that oxidative stress suppressed by antioxidants can in turn increase the stability of BACH1 and promote its accumulation in lung cancer cells. Accumulated BACH1 is involved in the mechanism promoting metastasis.

Recently, it has been reported that tumor cells with metastatic cancer potential have the superiority of genes coding bulky glycocalyx (a glycoprotein and glycolipid covering cell membranes) supporting metastasis (Paszek et al. 2014).

### Cellular senescence and aging

Aging is a process of progressive physiological changes leading to senescence or deteriorated biological functions of humans and all living organisms over the entire adult life span (Maldonado et al. 2023).

### Cellular senescence

Cellular senescence was observed in primary cells as progressive irreversible cell cycle arrest controlled by various mechanisms such as telomere shortening, oxidative stress, DNA replication stress, activation by oncogenes, and other factors (Devaraj et al. 2021).

Cellular senescence was observed for the first time in the early sixties in human fibroblasts during the establishment of optimal conditions of cell cultures (Hayflick and Moorhead 1961). Based on this and other studies, cellular senescence was related to the process of aging. Whereas progressive decay of the organism resulted in proliferative exhaustion, tumor cells, conversely were immortal and did not exhibit signs of cell senescence. These observations have led to the conclusion, that cells have a cell-division counting mechanism responsible for cell senescence (Kregel and Zhang 2007). In the cell senescence process, two main proteins, a tumor suppressor protein and critical cell cycle regulator Retinoblastoma protein (Rb) and p53 tumor suppressor protein are activated; their inactivation prevented senescence (Kulju and Lehman 1995). One of the best-documented mechanisms responsible for cell senescence is a pathway involving p53 (Herbig et al. 2004). In addition, oxidative stress is another important factor responsible for cell senescence. The suppressed activity of antioxidant enzymes and increased levels of hydrogen peroxide have been found to trigger premature senescence in human fibroblasts (Blander et al. 2003). Another important factor responsible for cell senescence is the high concentration of oxygen. Contrary to this, normal conditions including physiological concentration of oxygen resulted in a normal proliferation life span (Parrinello et al. 2003). Based on these observations, ROS-induced oxidative stress is involved in various steps of senescence signaling pathways, activating thus p53. Whereas significantly elevated p53 is directly associated with high ROS levels and the mechanism of apoptosis, a mild increase in p53 expression is associated with cell senescence accompanied by a small increase in oxidative stress (Macip et al. 2003). ROS released by the activation of Ras oncogene proteins (H-Ras, K-Ras4A, 4B, and N-Ras), regulators of signal transduction, can also trigger cell senescence through the activation of p53 (Lee et al. 1999). ROS can also promote telomere shortening (Bar-Or et al. 2001; Kregel and Zhang 2007).

### Aging

In 1935, Clive McCay and coworkers published a seminal paper in which they reported that rats on a restricted diet lived about 33% longer than it was known before (McCay et al. 1935). This work resulted in further studies on many species including also primates. In most of the studies animal’s intake of calories has been reduced by half of their normal levels. The results have shown that the lifespan has been extended between 50 and 300%, depending on the species (Grabski 2020; Flanagan et al. 2020).

The idea of caloric restriction originates from the rate of living theory that together with free radical theory represent two “classic” theories of the aging process. The rate of living theory came from the fact, that larger animals have longer lifespans than smaller animals. This was explained by the concept that larger animals have slower metabolic rates and longer lifespans than smaller animals (Sohal and Forster 2014).

Caloric restriction idea explains longer lifespan by suppressed metabolic rate as a result of significantly reduced intake of food (Heilbronn and Ravussin 2003), which in turn results in a decreased rate of free radical damage. This idea has been evidenced by animal experiments showing decreased ROS formation under caloric restriction (Heilbronn and Ravussin 2003). The slower rate of ROS formation means a lower level of ROS-induced damage to DNA, proteins, and membranes. From this follows that caloric restriction may slow down the aging process and allows the organism to live longer.

To determine quantitatively the effect of oxidative stress on aging, alterations in lifespan depending on the level of oxidative stress were studied in animals (Leyane et al. 2022; Iakovou and Kourti 2022). Alterations in ROS formation (oxidant load) have been studied either using a caloric restriction approach or alterations in mitochondrial antioxidant capacity via dietary supplementations. Caloric restriction is typically a 10–50% reduction of overall energy intake. It has been documented that caloric restriction modulates mitochondrial activity and suppressed oxidative damage through the sirtuin regulation and catalysis. Sirtuins are a family of proteins involved in the regulation of cell health (Zietara et al. 2023). They represent a group of seven proteins functioning in the presence of nicotinamide adenine dinucleotide (NAD+), a coenzyme occurring in all living cells vital to hundreds of biological processes. Sirtuins play an important role in antioxidant defense mechanisms and are known to prevent DNA damage, inflammatory reactions, and the formation of cancer cells (Watroba and Szukiewicz 2021).

Sirtuins are localized in cytoplasm (SIRT1, SIRT2, SIRT3, SIRT5), cell nuclei (SIRT1, SIRT2, SIRT3, SIRT6, SIRT7) and mitochondria (SIRT3, SIRT4, SIRT5), SIRT7 is the only sirtuin localized in the nucleolus. SIRT1 is localized in the nucleus, derived from *Saccharomyces cerevisiae* (yeast), and is the best described of all sirtuins (Selepe et al. 2022).

Short-term caloric restriction in mice increases expression of all seven sirtuin (SIRT1-SIRT7) proteins (Maldonado et al. 2021) and overexpression of telomerase reverse transcriptase (TERT) which reduces telomere erosion from various tissues such as muscles, kidney, and lungs (Vera et al. 2013).

It was also reported that a 20% reduction of calories in mice diet resulted in increased expression of SIRT1, decreased levels of superoxide radical anion, and increased expression of superoxide dismutase (Yzydorczyk et al. 2019). In addition, the concerted action of sirtuins has been found to stabilize telomers and alleviate telomere-associated disorders (Amano et al. 2019).

Reduced intake of energy compared to normal needs by 30% in obese rats increased the expression of SIRT3 in the liver and muscles (Tauriainen et al. 2011).

Being overweight significantly promotes an inflammation-related increase of oxidative stress of an organism. The major protective benefit of caloric restriction against cancer development is the expression of SIRT2 (Ahmed et al. 2020).

Forkhead box (FOX) proteins belong to FoxO family (forkhead boxO), and involve FOXO1 (FKHR), FOXO3A (FKHRL1), FOXO4 (AFX1), and FOXO6. They are transcriptional regulators controlling various biological functions involving activation/repression of gene expression and interaction with other factors such as p53 (Myatt and Lam 2007). Forkhead proteins present in mitochondria interact with sirtuins and affect the biological processes responsible for aging (Jerome et al. 2022). FoxO proteins participate in the differentiation and promotion of programmed cell death and regulate the level of oxidative stress and metabolism of lipids and sugars.

Alterations in the affinity of FOXO to DNA and modifications of transcriptional activity play a significant role not only in the pathology of chronic diseases such as cancer or diabetes but also affect the lifespan of organisms by activation of longevity genes such as SIRT6 responsible for the synthesis of antioxidants and HSP chaperones (Merksamer et al. 2013).

The interaction between sirtuins, FOXO, and p53 is very complex and appears to play a role in the survival of living organisms. The SIRT1-mediated deacetylation of p53, FOXO3, and other targets has a significant impact on apoptosis, inflammation, and mitochondrial function (Poulose and Raju 2015). SIRT1 can bind to FOXO4 and increase its activity by acetylation process. The activity of FOXO4 can be stimulated by FOXO accumulated in the nucleus which in turn increases the expression of genes responsible for the synthesis of antioxidant enzyme Mn-SOD. The activity of FOXO4 can be enhanced by resveratrol or conversely inhibited by nicotinamide, an inhibitor of SIRT1 (Xia et al. 2013).

In conclusion, the role of sirtuins as enzymes playing roles in longevity is still in the early stages of progression. A great pharmacological potential of sirtuins lies in the fact, that may simultaneously act as suppressors or promotors of cancer diseases. Based on many published studies and some facts outlined above, it is clear that aging and age-related diseases have a multifactorial and multilayered origin (Luo et al. 2020), however, one of the key factors is certainly oxidative damage. Due to this multifactorial origin, organ-focused interventions or single-component therapy approaches will not provide the desired effect. It is almost certain that one component is not enough to provide sufficient physiological effects due to the complexity of heterogenous pathways of organ deterioration especially in aging individuals. Clinical interventions related to oxidative stress should carefully consider the target population. Important is to choose a target group that will benefit the most from the studied substance. For example, vitamin E supplementation can partially improve cardiovascular diseases in the elderly population, especially those with diabetes (Vardi et al. 2013). Another important factor that should be considered is a reliable and proper biomarker. The reliable biomarker should be stable, quantitatively well analyzable, and reflect the redox state of an organism. The biomarker should sensitively reflect the level of oxidative damage from normal physiological functions to the disease state of the organism.

It is believed that further research will be devoted to the specification of key biomarkers of oxidative stress in the aging process and chronic disorders with the aim to find a direct relationship and causality between the level of these two conditions and oxidative stress/oxidative damage.

#### Antioxidants and aging

As discussed in the previous chapter, the interest in sirtuins has its origin in the impact on life extension. The aim is to find the effective and selective modulators of their activity (Wang et al. 2022). Sirtuins are involved in various metabolic pathways and alterations in their activity may affect different targets differently. For example, sirtinol, a potent non-specific inhibitor of SIRT2 and SIRT1 may induce inflammation in human monocytic leukemia, but at the same time sensitize prostate and cervical cancer cells to camptothecin (or its more water-soluble derivatives) and cis-Pt treatment (Lin and Fang 2013; Kumari et al. 2015).

**Fig. 24:**
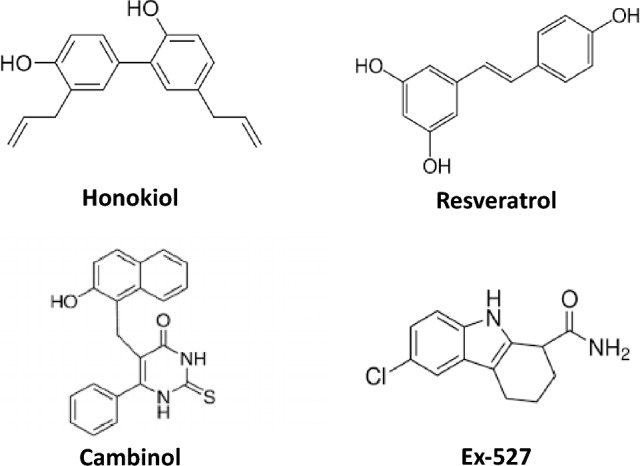
Structures of selected sirtuin modulators

Resveratrol is a polyphenol belonging to the stilbenoids group, present in more than 80 plant species (Fig. 24**)** (Salehi et al. 2018). Resveratrol together with structurally related polyphenols quercetin and fisetin are able to activate SIRT1. However, the problem is the bioavailability of polyphenols, and therefore their use in clinical praxis has serious limitations. To overcome problems with the bioavailability of flavonoids, various synthetic activators/inhibitors of sirtuins that mimic the health-beneficial properties of resveratrol and various polyphenols have been prepared and studied (Alcain and Villalba 2009). Several of them (SRT1720, SRT1460, SRT2183) are currently being evaluated in clinical trials (Zietara et al. 2023).

Resveratrol has been found to inhibit retrotransposition of Long Interspersed Nuclear Element-1 (LINE-1), comprising cca 17% of the human genome (Lander et al. 2001), which in turn is related to SIRT6 and SIRT7. Resveratrol has been reported to increase SIRT6 (Okudaira et al. 2022). Recent studies indicate that resveratrol interferes with various molecular pathways related to central nervous system and aging. Resveratrol beneficially affects epigenetic modifications (tags) regulating patterns of gene expression. Prevention of cognitive decline and cellular neurodegeneration has been related to the antioxidant properties of resveratrol (Grinan-Ferre et al. 2021).

ROS scavenging mechanisms interfere with the pathways responsible for antiapoptotic and anti-inflammatory properties (Miguel et al. 2021). Decreased neuroinflammation influenced by the effect of resveratrol in rats is documented by the decreased levels of proinflammatory cytokines such as TNF-α.

Honokiol (HKL) (Fig. 24) is a multifunctional polyphenol extracted from the bark and leaves of the genus *Magnolia* (Fried and Arbiser 2009). Honokiol has the ability to cross the blood–brain barrier as well as the blood-cerebrospinal fluid barrier which points to the significant bioavailability of this polyphenol. SOD1-G93A transgenic mice are characterized by expressed G93A mutant form of hSOD1 suitable for neuromuscular disorder studies. Honokiol has been shown to extend the lifespan of SOD1-G93A transgenic mice and improve their motor function and the viability of neuroblastoma/spinal cord NSC-34 hybrid cell lines and the morphology of mitochondria in SOD1-G93A cells (Zhou et al. 2023). Honokiol can promote cell viability and endoplasmic reticulum stress modulation by SIRT1 expression (Khatoon et al. 2022).

Cambinol (Fig. 24) is a β-naphthol derivative possessing anticancer and sirtuin 1 and sirtuin 2 inhibiting properties. Cambinol inhibits NAD-dependent deacetylases implicated in the regulation of many biological processes including metabolic control (Lugrin et al. 2013).

Ex-527 is an indole matrix for the synthesis of sirtuin inhibitor analogs. It has been reported that Ex-527 acts as a SIRT1 and necroptosis (an emerging mode of program cell death) inhibitor in animal models (Wang et al. 2012; Nikseresht et al. 2019). Ex-527 has also been reported to protect the organism against kidney damage caused by diabetes (diabetic nephropathy). This effect is achieved by the reduction of glucose levels and an increase in SIRT1 activities accompanied by the simultaneous increase in SIRT3 activity in the kidneys (Kundu et al. 2020).

Ex-527 (Fig. 24) is a potent and selective Sirt1 inhibitor. In the study of anticancer properties of sirtuin inhibitors, it has been found, that sirtuins play a central role in platelet aging. This suggests that the process of sirtuins inhibition involves the restriction of platelet life span and management of thrombosis (Kumari et al. 2015).

Ergothioneine (see also above) is highly abundant in mushrooms and their consumption has been found to positively influence cognitive impairment in elderly people, confirming the importance of ergothioneine in healthy aging (Feng et al. 2019).

Ergothioneine is a naturally occurring amino acid resistant to autooxidation occurring in solution in the thione form (Cheah et al. 2019). Ergothioneine plays an important role in aging-related signaling pathways. Ergothioneine has been found to play a role in survival signaling pathways, predominantly by stimulating phosphorylation of AKT/PKB signaling pathway in neuronal cells under mild, hydrogen peroxide-induced oxidative stress (Colognato et al. 2006).

Ergothioneine has also been shown to exhibit anti-aging properties in UVA-irradiated human fibroblast cells through activation of the P13K-AKT pathway (Hseu et al. 2020).

The knockdown of the ergothioneine transporter in C. elegans (a transparent 1 mm long nematode model organism) reduces their lifespan and increases their susceptibility to ROS-induced damage (Cheah et al. 2013).

The family of Forkhead box (FOX) transcription factors consists of a conserved group of transcriptional regulators important in various developmental functions. A recent study reported that ergothioneine, even at very low concentrations (at the nanomolar level) exhibits concentration-dependent cytoprotective properties on erythroid cells via triggering FOXO transcription factor (Bernardo 2021).

It is known that direct or indirect inhibition of mTOR signaling is important in the extension of lifetime. Contrary to these studies, it has been reported that ergothioneine activates the mTOR pathway which in turn may activate neurotrophic factor signaling, the regulator of neuronal differentiation (Ishimoto et al. 2018). This indicates that both positive and negative signals may activate mTOR.

Treatment with ergothioneine stabilizes the content of glutathione in the brain of aging flies (Pan et al. 2022). This points to the importance of ergothioneine in health span increase by stabilizing reduced form of glutathione (GSH).

Taken together, ergothioneine is an agent suppressing the level of oxidative stress, modulating gene expression, and interfering with signal pathways, promoting thus longevity. The structure of ergothioneine allows the chelation of redox metals which escaped from homeostasis and the upregulation of antioxidants via the Nrf2 pathway which in turn results in the prevention of the aging process. Considering that the main aim is to increase healthy aging rather than lifespan, ergothioneine appears to be a suitable candidate for further clinical testing (Apparoo et al. 2020).

## Conclusions and outlook

A great number of physiological functions in living systems are mediated by electron transfer (redox) reactions shifted from thermodynamic equilibrium. Transfer of electrons is involved in cell bioenergetics, oxidative phosphorylation, DNA damage, enzymatic catalysis, metabolism of drugs, and other processes. The direct consequence of the electron transfer reactions in living systems is the transient presence of free radicals (ROS, RNS).

The role of ROS/RNS in biological systems is usually associated with the term oxidative stress, a phenomenon responsible for oxidative damage in biological systems. However, oxidative stress is a “double edge sword” for living systems, as it is important for physiological signaling mediated by physiological concentrations of ROS (from ~ 10^−12^ M to ~ 10^−6^ M), termed “oxidative eustress”, while it can be responsible for oxidative damage to biomolecules, a process termed “oxidative (di)stress”, mediated by increased ROS concentration (> 10^−6^ M) (Murphy et al. 2022; Azzi 2022).

Oxidative stress (distress) is a common denominator of a wide range of diseases including cancer, neurological disorders such as Alzheimer’s disease, lung diseases, cardiovascular diseases and other disorders collectively characterized by multiple mechanisms by which ROS cause cellular damage. One of the major mechanisms through which oxidative stress causes damage to biomolecules is their direct oxidation by ROS such as hydroxyl radical or peroxynitrite. Another important mechanism of oxidative stress-mediated damage is disturbed redox signalling. Diabetes mellitus is an example where both these mechanisms occur simultaneously.

The historically important question, which has been the subject of frequent discussions, is whether oxidative stress is the primary cause or secondary consequence of the disease. Oxidative stress as the primary cause has been observed in atherosclerosis and radiation-induced diseases such as lung injury. The primary cause of oxidative stress has also been observed in herbicide paraquat poisoning, characterized by a redox cycling mechanism involving superoxide radical anion formation triggered by exogenous NADPH in pulmonary endothelial cells (Tampo et al. 1999). Oxidative stress as a second line of attack occurs after the onset of initial pathology, documented, for example, by the formation of superoxide radicals and hydrogen peroxide by NADPH oxidases, for example, in the response to injury-mediated inflammation. Oxidative stress is responsible for disturbed signalling pathways which in turn affects, for example, molecular mechanisms of apoptosis induction, impairment of mitochondrial function, protein modification, and other mechanisms. Oxidative stress as a secondary factor in disease progression has been observed in chronic pulmonary disease, type 2 diabetes, Alzheimer’s disease, cancer, and other disorders. All these disorders have in common the occurrence of oxidative stress via multiple pathways. For example, cancer cells produce a high amount of ROS and therefore they have to cope with enhanced oxidative stress.

Organisms have evolved a complex and sophisticated system of defense against oxidative stress. The defense system not only boosts the capacity to detoxify oxidants but also repairs oxidative damage. The primary and most efficient system is represented by antioxidant enzymes. Several transcription factors such as activator protein (AP-1), nuclear factor kappa B (NF-κB), and predominantly the nuclear factor erythroid 2–related factor 2 (Nrf2) are involved in the induction of antioxidant enzymes.

The antioxidant efficiency of antioxidant enzymes is significantly higher than the group of low molecular weight antioxidants. ROS, such as hydroxyl radicals, peroxynitrite, and other radicals react very rapidly with DNA, proteins, and membrane lipids to be scavenged by exogenous small molecular antioxidants. The rate constants of ROS removal by low molecular weight antioxidants within the cells are negligible compared to the scavenging activity of intracellular enzymes. The most effective strategy to prevent oxidative damage by ROS (e.g., hydroxyl radicals) is to prevent their formation.

The first line of antioxidant defense against ROS is very efficient and is predominantly represented by the intracellular antioxidant pool, consisting of antioxidant enzymes, including Cu,Zn-SOD (SOD1), catalase, glutathione peroxidase (GPX), peroxiredoxins (PRDx). The extracellular matrix of tissues contains the major extracellular antioxidant enzyme SOD (EC-SOD or SOD3). In addition to small-molecular antioxidants, extracellular antioxidant defense against ROS is also mediated by SOD mimetic compounds, capable of scavenging superoxide radicals and preventing the formation of damaging peroxynitrite, formed by the reaction between superoxide and nitric oxide. However, the kinetics of superoxide radical removal by SOD mimetic compounds is much less effective than by SOD enzymes. Some metallodrugs have been synthesized and tested for their combined, SOD and catalase scavenging activities.

The second line of antioxidant defense involves the synthesis of a thioredoxin redox system in which reduced thioredoxin (TRX) catalyzes the reduction of disulfides (–S–S–) and is itself oxidized and subsequently reduced by thioredoxin reductase at the expense of NADPH. In addition, glutathione reductase (GR) in the presence of NADPH restores reduced glutathione (GSH) from its oxidized GSSG form. Both the first and second lines of antioxidant defense play also a role in redox signaling and maintenance of redox homeostasis. 

The third line of antioxidant defense is represented by the removal of oxidized biomolecules (Sies 1993b). Proteins are one of the most abundant biomolecules and are a frequent target of ROS attacks. Maintenance of cellular functions is mediated by the two proteolytic systems, proteasomal and lysosomal systems involved in the removal of irreversibly damaged proteins and amino acid recycling for protein synthesis.

All three above-discussed lines of antioxidant defense mediated by their concerted action maintain the antioxidant status of an organism.

The mosaic of the antioxidant network in living systems also includes low-molecular-weight antioxidants derived from the diet, probably the most important being vitamins C and E, carotenoids, and flavonoids (polyphenols). The last few decades have been associated with great expectations regarding clinical studies of the effect of low molecular antioxidants on human health. Unfortunately, the outcomes are questionable. Apart from vitamin E, the evidence, that other low molecular weight antioxidants show in vivo effects is not very convincing. Megadose supplementation of vitamins has been shown to be ineffective at suppressing oxidative damage in the human body. Paradoxically promising appears to be the use of certain antioxidants (e.g. flavonoids) showing weak prooxidant properties, that may boost cellular antioxidant systems, involving antioxidant enzymes and synthesis of glutathione and thus act as preventive anticancer agents. A promising antioxidant appears to be ergothioneine, however, double-blind placebo-controlled trials have to be conducted.

Another promising use of low molecular antioxidants is their potential ability to activate certain signaling pathways. The antioxidant status of an organism can be enhanced by the NRF2 activators which appear to represent a promising therapeutic approach. Activation of NRF2 results predominantly in the induction of antioxidant enzymes. NRF2 signaling can be activated by flavonoids and antioxidants contained in tea, vegetables, and fruit extracts. However, there are several concerns associated with the therapeutic applications of NRF2 activators. Many of these activators are Lewis acids capable of accepting electron pairs and/or donating protons. Such substances are quickly metabolized and therefore their bioavailability is rather low. Michael adducts of nucleophiles with electrophilic compounds are one way to overcome this problem. Another concern about the NRF2 activation is a lack of specificity. NRF2 activators are not restricted to a specific organ or tissue and therefore may exhibit various side effects and disruption of physiological mechanisms.

Since major antioxidant protection of living systems is maintained by the antioxidant enzymes, a significant effort is being devoted to the development of therapeutic strategies centered on the design, in vitro, and clinical studies of enzyme mimetic compounds. Frequently studied are metal-based SOD and SOD-catalase mimetic compounds of which several are currently undergoing clinical trials. Since redox metal manganese may exist in several oxidation states, attention has been focused on Mn-cyclic polyamines, Mn-salen complexes, and Mn- and Fe-based metalloporphyrins. A general feature of all these compounds is that they can effectively accommodate superoxide radical anions on the metal center. These compounds appear to be effective in extracellular space, where the concentration of antioxidant enzymes is usually much lower than in the intracellular environment. However, these compounds may show a lower degree of specificity and reduce other ROS/RNS species. In addition to SOD-mimetic compounds, Salen-based organic compounds, possessing hydrogen peroxide scavenging activity have been prepared and tested as Glutathione peroxidase mimetics.

## Data Availability

Data sharing is not applicable to this article as no new data were created or analyzed in this review.
